# VC-resist glioblastoma cell state: vessel co-option as a key driver of chemoradiation resistance

**DOI:** 10.1038/s41467-024-47985-z

**Published:** 2024-04-29

**Authors:** Cathy Pichol-Thievend, Oceane Anezo, Aafrin M. Pettiwala, Guillaume Bourmeau, Remi Montagne, Anne-Marie Lyne, Pierre-Olivier Guichet, Pauline Deshors, Alberto Ballestín, Benjamin Blanchard, Juliette Reveilles, Vidhya M. Ravi, Kevin Joseph, Dieter H. Heiland, Boris Julien, Sophie Leboucher, Laetitia Besse, Patricia Legoix, Florent Dingli, Stephane Liva, Damarys Loew, Elisa Giani, Valentino Ribecco, Charita Furumaya, Laura Marcos-Kovandzic, Konstantin Masliantsev, Thomas Daubon, Lin Wang, Aaron A. Diaz, Oliver Schnell, Jürgen Beck, Nicolas Servant, Lucie Karayan-Tapon, Florence M. G. Cavalli, Giorgio Seano

**Affiliations:** 1 Institut Curie, INSERM U1021, CNRS UMR3347, Tumor Microenvironment Lab, Paris-Saclay University, 91400 Orsay, France; 2 Institut Curie, PSL University, 75005 Paris, France; 3 INSERM U900, 75005 Paris, France; 4 MINES ParisTeach, CBIO-Centre for Computational Biology, PSL Research University, 75006 Paris, France; 5 Université de Poitiers, CHU Poitiers, ProDiCeT, F-86000 Poitiers, France; 6 CHU Poitiers, Laboratoire de Cancérologie Biologique, F-86000 Poitiers, France; 7 Department of Neurosurgery, Medical Center - University of Freiburg, Freiburg, Germany; 8 Histology Facility, Institut Curie, 91400 Orsay, France; 9 Institut Curie, PSL University, Université Paris-Saclay, CNRS UMS2016, INSERM US43, Multimodal Imaging Center, 91400 Orsay, France; 10 Institut Curie, PSL University, ICGex Next-Generation Sequencing Platform, 75005 Paris, France; 11 Institut Curie, PSL University, CurieCoreTech Spectrométrie de Masse Protéomique, 75005 Paris, France; 12 Department of Biomedical Sciences, Humanitas University, 20072 Pieve Emanuele, Italy; 13 Université Bordeaux, CNRS, IBGC, UMR5095, Bordeaux, France; 14 Department of Computational and Quantitative Medicine, Hematologic Malignancies Research Institute and Beckman Research Institute, City of Hope, Duarte, CA USA; 15 Department of Neurological Surgery, University of California, San Francisco, San Francisco, CA USA

**Keywords:** CNS cancer, Cancer microenvironment, Tumour heterogeneity, Cancer

## Abstract

Glioblastoma (GBM) is a highly lethal type of cancer. GBM recurrence following chemoradiation is typically attributed to the regrowth of invasive and resistant cells. Therefore, there is a pressing need to gain a deeper understanding of the mechanisms underlying GBM resistance to chemoradiation and its ability to infiltrate. Using a combination of transcriptomic, proteomic, and phosphoproteomic analyses, longitudinal imaging, organotypic cultures, functional assays, animal studies, and clinical data analyses, we demonstrate that chemoradiation and brain vasculature induce cell transition to a functional state named VC-Resist (vessel co-opting and resistant cell state). This cell state is midway along the transcriptomic axis between proneural and mesenchymal GBM cells and is closer to the AC/MES1-like state. VC-Resist GBM cells are highly vessel co-opting, allowing significant infiltration into the surrounding brain tissue and homing to the perivascular niche, which in turn induces even more VC-Resist transition. The molecular and functional characteristics of this FGFR1-YAP1-dependent GBM cell state, including resistance to DNA damage, enrichment in the G2M phase, and induction of senescence/stemness pathways, contribute to its enhanced resistance to chemoradiation. These findings demonstrate how vessel co-option, perivascular niche, and GBM cell plasticity jointly drive resistance to therapy during GBM recurrence.

## Abstract (web summary)

In patient with glioblastoma, a major cause of resistance to chemotherapy and radiotherapy is the high degree to intratumoral heterogeneity and cell plasticity. Here, the authors demonstrate that chemoradiation induces the reprograming of glioblastoma cells into an invasive and vessel co-opting state, termed VC-Resist, capable of promoting resistance to therapy.

## Introduction

Glioblastoma (GBM) is the most common malignant primary brain cancer of the central nervous system in adults^1,2^. Although GBM is a relatively rare tumor, it is one of the biggest challenges in translational science for two reasons: the very high mortality rate and lack of therapeutic improvement over the last 20 years^3,4^. The current standard treatment regimen for patients with GBM consists of maximal safe surgical resection, followed by radiotherapy and temozolomide (TMZ) chemotherapy^5^. The two most clinically relevant challenges faced by patients with GBM are chemoradiation resistance and extensive infiltration of the peritumor regions. Indeed, chemoradiation is insufficient to prevent regrowth of infiltrative therapy-resistant cells that are not removed by resection.

This chemoradiation resistance is partially due to tumor cell-intrinsic mechanisms such as GBM heterogeneity and plasticity^6^. Indeed, GBM is characterized by several levels of heterogeneity. The first level is intertumoral heterogeneity with three major GBM subtypes: proneural (PN), classical (CL), and mesenchymal (MES)^7,8^. Furthermore, different subtypes have been shown to coexist within the tumor tissue of a single GBM patient, representing intratumoral heterogeneity^9,10^. Moreover, single cell heterogeneity at both the transcriptional and epigenetic levels adds another layer of complexity^11,12^, unraveling functional cell states such as oligodendrocyte-progenitor-like (OPC-like), neural-progenitor-like (NPC-like), mesenchymal-like (MES-like) and astrocyte-like (AC-like) cells that partly determine the subtypes^11^. The transitions between these cell states, also called cell plasticity, occur in GBM cells and are believed to be important determinants of chemoradiation resistance and tumor development^11,13–16^. Except for recent reports^17–19^, little is known about how therapeutic stress and microenvironment dynamically modulate the plasticity of these cellular states or others.

The recurrence and re-growth of therapy-resistant GBM cells are also due to the typically high GBM infiltration of the peritumor brain regions. Among the distinct invasion strategies used by GBM cells, vessel co-option is remarkable because it may be a link between chemoradiation resistance, tumor cell plasticity and infiltration far from the tumor bulk. Vessel co-option is the active movement of tumor cells towards blood vessels and at the invasive front of GBM it gives rise to perivascular satellitosis, one of the GBM hallmarks^20–23^. Moreover, the perivascular niche is a reservoir of protective factors that may induce GBM cells survival, resistance to therapy, progression and dissemination^21,24,25^. However, whether and how infiltrative vessel co-option and perivascular niche are relevant during GBM therapy remains unclear.

In our study, we demonstrate that chemoradiation therapy can cause GBM cells to undergo a reprogramming into a vessel co-opting and invasive cell state, which we have designated as VC-Resist (acronym for vessel co-opting resistant). This cell state – basally present in naïve cell populations but also induced by therapy – is intermediate in the proneural-mesenchymal axis, partially reversible, senescent- and stem-like, slow-cycling, resistant to therapy and characterized by FGFR1 upregulation, as well as YAP1 and DNA-damage repair (DDR) machinery activation. Additionally, this vessel co-opting cell state is extrinsically induced by blood vessels, leading to a local increase in its resistance to treatment.

## Results

### Tracking cell state transitions reveals that γ-irradiation induces GBM reprogramming

**Fig. 1:**
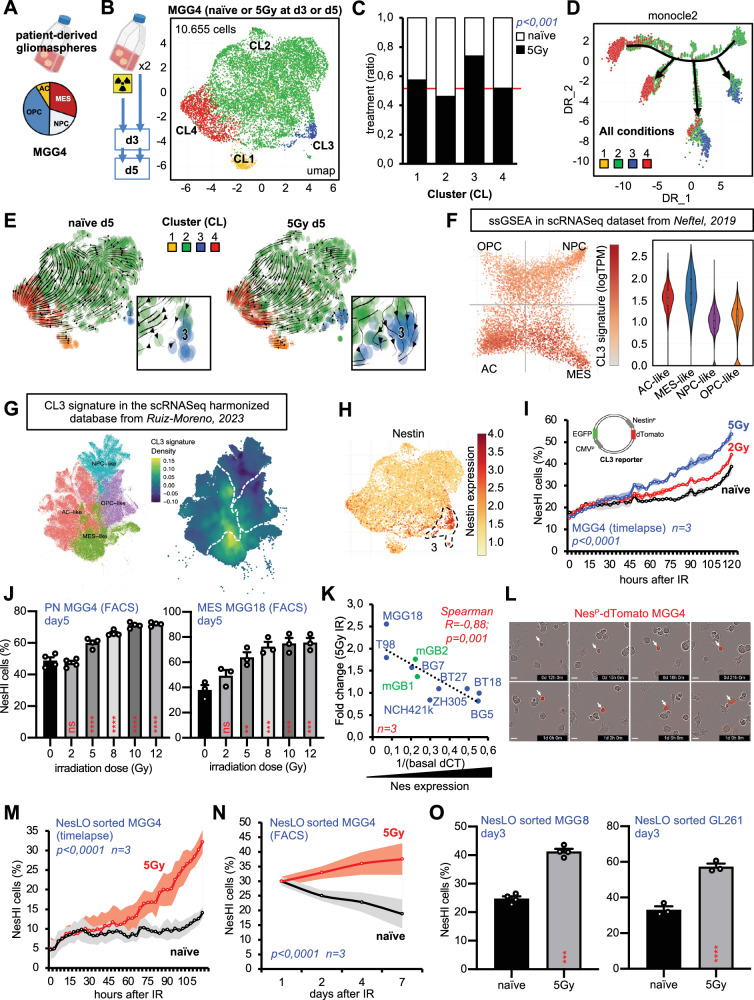
scRNA-seq analysis unveils a GBM radiotherapy-induced cell state transition. **A** Cell state heterogeneity in MGG4 gliomaspheres: astrocyte (AC)-like, oligodendrocyte progenitor cells (OPC)-like, mesenchymal (MES)-like and neural progenitor cells (NPC)-like. Schematics created with BioRender.com. **B** UMAP dimensionality reduction plot of scRNA-seq for MGG4 gliospheres exposed to 5 Gy IR or not, at day3 or 5 of culture (*n* = 2 independent experiments). **C** Effect of IR on each cluster (*p* = 0,0005; Chi-square). **D**, **E** RNA velocity and trajectory analysis of irradiated or naïve MGG4 cells at day5. **F** Expression of CL3 geneset in the Neftel’ representation of cell states. GBM cell positions indicate relative scores for meta-modules, with colors reflecting CL3 geneset expression. Violin and box-and-whiskers plot (Tukey) from 28 GBM patients produced with https://singlecell.broadinstitute.org. **G** Feature plot for CL3 signature in the harmonized database with over 1 M GBM cells. **H** Feature plot of Nestin expression. **I** Enrichment of NesHI cell population upon IR (2 and 5 Gy) in Nestin^P^-dTomato MGG4 cells analyzed by real-time microscopy. Data are means ± SEM (*n* = 3 independent experiments, technical duplicates per experiment; *p* < 0.0001; one-way ANOVA, Turkey’s multiple comparisons test). **J** Enrichment of NesHI cell population upon IR (2, 5, 8, 10, 12 Gy) analyzed at day5 by FACS in Nestin^P^-dTomato MGG4 and MGG18 cells. Data are means ± SEM (MGG4 *n* = 4; MGG18 *n* = 3; ns, non-significant; *p < 0.05; **p < 0.01; ***p < 0.001; ****p < 0.0001; one-way ANOVA, Turkey’s multiple comparisons test). **K** Spearman correlation analysis of basal Nestin expression and fold change (FC) of Nestin expression after irradiation (5 Gy) in 10 GBM cell lines. *X-*axis: 1/basal CT values for Nestin expression determined by RT-PCR; *Y-*axis: Nestin FC after IR determined by RT-PCR. Patient-derived (blue) mouse (green) cell lines (*n* = 3 per cell line, R = 0,88; *p* = 0,001, Spearman test). **L** Time-lapse micrographs of FACS-sorted MGG4 NesLO cells showing Nestin reporter activation (arrows). **M**, **N** Enrichment of MGG4 NesHI cell population overtime upon IR (5 Gy) or not (Naive) in FACS-sorted MGG4 NesLO cells analyzed by real-time microscopy and FACS. Data are means ± SEM (*n* = 3 biologically independent experiments, *p* < 0.0001, Pearson test). **O** Enrichment of NesHI cell population under IR (5 Gy) in FACS-sorted MGG18 and GL261 NesLO cells analyzed at day3 by FACS. Data are means ± SEM (MGG18 *n* = 4; GL261 *n* = 3; ***p < 0.001; ****p < 0.0001; unpaired two-sided *t* test).

Recently, many studies have classified GBM into different states at the single-cell level; however, little is known about how therapeutic stress modulates these cellular states. To focus our attention on the intrinsic transcriptomic programs of GBM cells, we decided to study a patient-derived cell line cultured as a gliomasphere, the IDH-wt MGG4 cell line^26^. The co-existence in this cell line of the four distinct cellular states from Neftel et al. ^11^, i.e. the AC-like, MES-like, OPC-like and NPC-like (Fig. 1A), makes it a highly relevant model for studying the heterogeneity—and potentially plasticity—of cellular states in the GBM. We subjected MGG4 gliomaspheres to 5 Gy of γ-irradiation (IR) – a dose that causes approximately 20% cell death in this cell line (Supplementary Fig. 1A)—and profiled more than 10.000 cells using scRNA-seq on days 3 and 5 after IR. No radical changes in cell state proportions from Neftel’s cell classifier were detected upon IR (Supplementary Fig. 1B, C), with a slight enrichment in the AC-like state, as previously reported in the literature^17^. Therefore, we examined our scRNA-seq dataset using unsupervised clustering to identify the cell phenotypes that were specifically enriched after IR (Fig. 1B and Supplementary Fig. 1D). IKAP^27^ identified 4 clusters in our cell line (CL1, CL2, CL3, and CL4). IR had a significant impact on only one of the clusters, CL3 (Fig. 1C), that—although basally not highly represented in the MGG4 cell line—was two-fold-increased by IR, both at day 3 and 5 (Fig. 1C, Supplementary Fig. 1E). Trajectory analysis indicated that IR diverts the trajectories seen in naïve MGG4, by specifically generating or reinforcing a trajectory that leads to CL3 (Fig. 1D, Supplementary Fig. 1F) and RNA velocity analysis showed that IR changes the cell state transition occurring in MGG4 cells with the development and enrichment of CL3 (Fig. 1E, Supplementary Fig. 1G).

The CL3 marker genes suggested hybrid features, with NPC-like (such as *MAP1B*, *MEST*, and *LBH*), AC-like (such as *SPARC* and *NES*), and MES-like (*VIM*, *A2M*, and *CDKN1A*) genes being the top markers. To understand the CL3 features in relation to GBM classifiers, we investigated the public Neftel dataset and the recently available harmonized GBM database with over 1 M cells^28^. We observed that CL3 cells spread between the AC-like and MES-like cell states (Fig. 1F) and between the cell states (Fig. 1G), thus suggesting hybrid characteristics.

These scRNA-seq analyses indicated radiation-induced reprogramming of GBM cells towards the CL3 phenotype. Hence, to track CL3 cells, we searched for a specific and suitable marker for CL3 in MGG4 cells. We choose Nestin, an intermediate filament protein used as a glioma stem cell (GSC) marker^29^, as it is highly and significantly CL3-specific and has well-studied reporters of its expression (Fig. 1H, Supplementary Fig. 1H). Therefore, we built a fluorescent reporter of Nestin expression, using a previously published reporter^30^, and extended our investigation to three GBM cell lines with different mutational landscapes, phenotypic subtypes and species (Supplementary Data File 1). We first tested the reporter efficiency for the proper detection of Nestin expression via qPCR and in situ single-cell mRNA quantification (Supplementary Fig. 2A, B). Next, using real-time imaging and FACS analysis, we quantified the Nestin^P^-dTomato-transduced PN-MGG4, MES-MGG18 and MES-GL261 GBM cells at different IR doses. IR progressively increased the percentage of Nestin-high (NesHI) cells and the reporter fluorescence per cell in a dose-dependent manner (Fig. 1I, J, Supplementary Fig. 2C–E). We then generalized this finding by analyzing eight other patient-derived and two mouse GBM cell lines. The impact of IR on Nestin upregulation was larger in cell lines with lower basal levels of Nestin (Fig. 1K), by suggesting a marked intertumoral heterogeneity in the number of CL3+ cells.

Our single-cell analyses suggested that the post-therapy enrichment of NesHI cells is due to an active reprogramming of the NesLO cells, and not only a selection process. To test this reprogramming hypothesis, we used NesLO-sorted cells. IR actively induced cell state transition in NesLO cells towards NesHI (Fig. 1L–N, Supplementary Fig. 2F–H). Notably, the radiotherapy-induced NesLO-to-NesHI transition was also confirmed in MGG18 and GL261 cell models (Fig. 1O).

Taken together, our findings demonstrate a cell state transition induced by radiotherapy in GBM cells.

### The DNA-damaging chemotherapy temozolomide (TMZ) induces GBM reprogramming

**Fig. 2:**
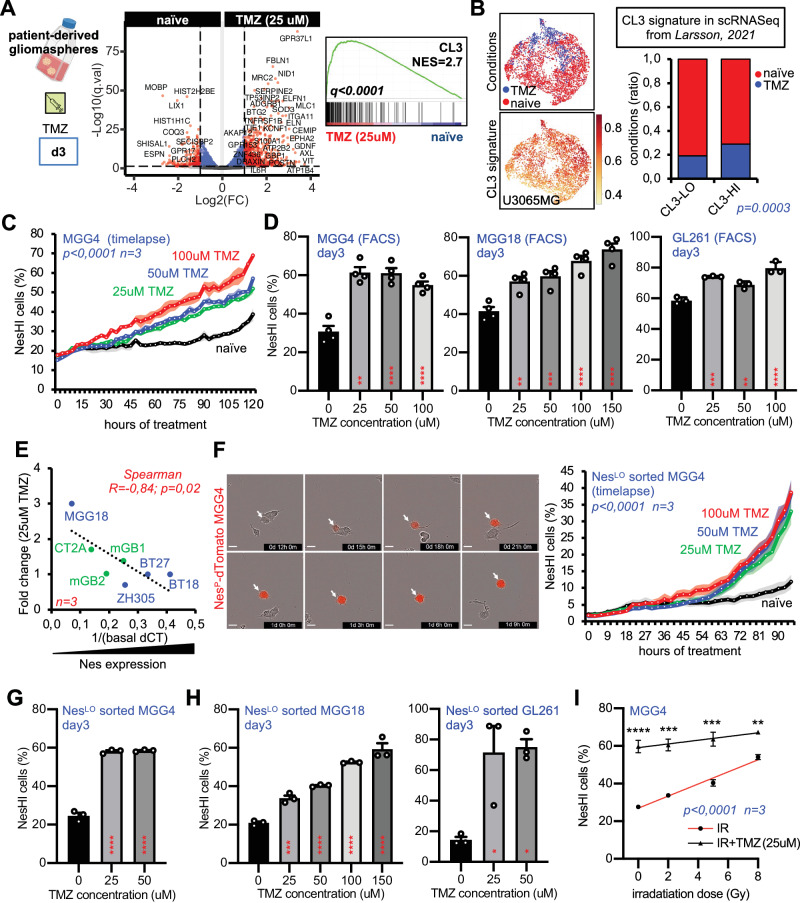
TMZ treatment induces GBM cell state transition. **A** (Left)Volcano plot of the differential expression analysis between MGG4 naïve and treated with TMZ (25 μM) for 3days. (Right) GSEA plot of the CL3 signature in TMZ-treated MGG4 cells vs naïve. Normalized enrichment score (NES) and q.value are indicated (*p* = 0,0003; Fisher’s test). **B** CL3 signature distribution in the scRNA-seq dataset from Larsson, 2021. (Left) Visualization of conditions (naïve or TMZ-treated U3065MG cells) and feature plot of CL3 geneset expression in scRNA-seq UMAP. (Right) Proportions of CL3-HI or CL3-LO cells (*p* = 0.0003; z-test). **C** NesHI cell population upon TMZ treatment (25, 50 and 100 μM) in Nestin^P^-dTomato MGG4 cells by real-time microscopy (*n* = 3 biologically independent experiments, *p* < 0.0001 vs naïve cells, Pearson test). **D** NesHI cell population upon TMZ treatment (25, 50 and 100uM) at day3 by FACS in Nestin^P^-dTomato MGG4, MGG18 and GL261 cells. Data are means ± SEM (*n* = 4 independent experiments; ns, non-significant; **p < 0.01; ***p < 0.001; ****p < 0.0001; one-way ANOVA, Turkey’s multiple comparisons test). **E** Spearman correlation analysis of basal Nestin expression and Nestin fold change (FC) of upon TMZ (25 μM) in 7 GBM cell lines. *X-*axis: 1/basal CT values for Nestin expression by RT-PCR. *Y-*axis: Nestin FC after IR. Patient-derived (blue) mouse (green) cell lines (*n* = 3; R = 0,84; *p* = 0,001; Spearman test). **F** (Left) Time-lapse micrographs of FACS-sorted MGG4 NesLO cells showing the reprogramming detected by dTomato fluorescence (arrow). (Right) of NesHI cell population enrichment upon TMZ treatment in FACS-sorted NesLO MGG4 cells. (*n* = 4 biologically independent experiments, *p* < 0.0001 vs naïve cells, Pearson test). **G** NesHI cell population upon TMZ treatment (25 and 50 μM) in FACS-sorted MGG4 NesLO cells analyzed at day3 by FACS. Data are means ± SEM (*n* = 3 independent experiments; ****p < 0.0001; one-way ANOVA, Turkey’s multiple comparisons test). **H** NesHI cell population enrichment upon TMZ treatment in FACS-sorted MGG18 (left) and GL261 NesLO cells (right) analyzed at day3 by FACS. Data are means ± SEM (*n* = 3; *p < 0.05; ***p < 0.001, ****p < 0.0001; one-way ANOVA, Turkey’s multiple comparisons test). **I** NesHI cell population upon IR alone or combinatorial therapy (25 μM TMZ and indicated IR dose) in Nestin^P^-dTomato MGG4 by FACS at day3. Data are means ± SEM (*n* = 3 independent experiments; ***p < 0.001, ****p < 0.0001 vs the correspondent IR doses; two-way ANOVA, Turkey’s multiple comparisons test).

Both IR and TMZ used in the standard of care are DNA-damaging therapies for GBM cells^5,31^. We therefore investigated whether the CL3 geneset may be considered a reliable indicator of GBM DNA-damaging therapy-induced cell response. We therefore investigated MGG4 cells subjected to TMZ treatment. To do so, we selected the 150 most significantly upregulated genes in scRNA-seq CL3 compared with the rest of the cells (Supplementary Data File 2 and Supplementary Fig. 3A). Notably, the CL3 geneset did not overlap with the Neftel genesets (Supplementary Fig. 3B). We therefore treated MGG4 cells with 25 μM of TMZ for 3 days and performed gene set enrichment analysis (GSEA) using bulk RNA-seq. At this dose, approximately 40% of MGG4 cells died (Supplementary Fig. 3C). Interestingly, GSEA showed a strong enrichment of the CL3 geneset in TMZ-treated versus naïve cells (Fig. 2A).

Next, we verified the relevance of the CL3 signature in a recently published and independent scRNA-seq dataset of TMZ-treated GBM patient-derived cell line^19^. The proportion of cells with the highest CL3 score significantly increased after TMZ treatment (Fig. 2B).

We then challenged Nestin^P^-dTomato MGG4, MGG18 and GL261 cells with TMZ. Like IR, TMZ treatment induced a gradual and dose-dependent increase of NesHI cells proportions in MGG4, MGG18 and GL261 (Fig. 2C, D, Supplementary Fig. 3D–F), which is in line with previous reports^32^. As upon IR, the basal levels of Nestin were inversely correlated with the magnitude of Nestin increase induced by TMZ treatment (Fig. 2E). Finally, we specifically tested whether TMZ induces cell reprogramming as occurs upon IR and treated NesLO cells with TMZ at different doses and times. Real-time imaging and FACS analysis showed that TMZ treatment induced reprogramming of NesLO cells to NesHI in a dose-dependent manner (Fig. 2F–H, Supplementary Fig. 3G). Finally, to investigate the combinatorial effect of IR and TMZ on cell reprogramming, we challenged GBM neurospheres with concomitant treatments, as in clinical practice. IR and TMZ appeared to have an additive effect on their ability to induce reprogramming to the CL3/NesHI cell state (Fig. 2I).

Collectively, these results indicate that TMZ chemotherapy induces reprogramming towards the cell state we discovered, thus making it a GBM DNA-damaging therapy-induced cell state.

### Preclinical and clinical validation of the therapy-induced functional state

**Fig. 3:**
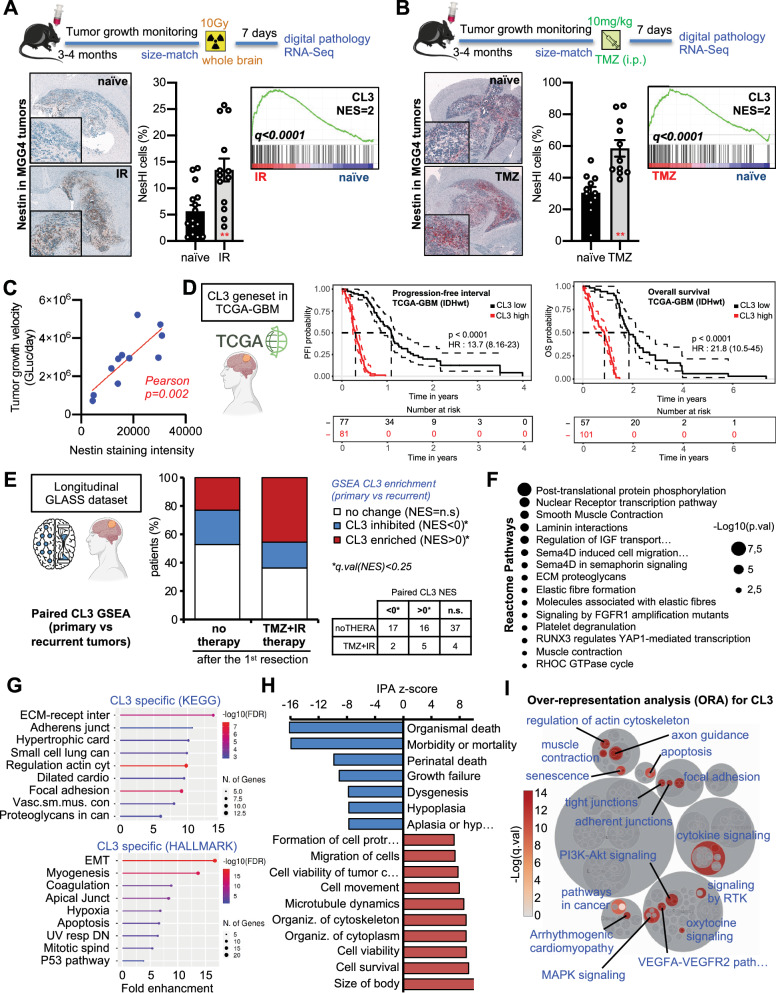
Preclinical and clinical validation of the therapy-induced functional GBM state. **A** (Top) MGG4-GFP-Gluc intracranially implanted and subjected to whole-brain irradiation. (Left) Nestin immunostaining (Nestin, brown; hematoxylin, blue) and quantification of Nestin positive nuclei. Data are means ± SEM (*n* = 15 naïve, *n* = 13 IR-10Gy; **p < 0.01; unpaired two-sided *t* test). Scale bar, 100 μm. (Right) GSEA plot of the CL3 signature after in vivo IR. Only the probes aligned to human genes were taken in account. The normalized enrichment score (NES) and q.value are indicated. **B** (Top) MGG4-GFP-Gluc intracranially implanted and treated with TMZ. (Left) Nestin immunostaining (Nestin, red; hematoxylin, blue) and quantification of Nestin positive nuclei. Data are means ± SEM (*n* = 11 naïve (DMSO), *n* = 11 TMZ (10 mg/kg); **p < 0.01, unpaired two-sided *t* test). Scale bar, 100 μm (Right) GSEA plot of the CL3 signature after in vivo TMZ-treatment. Only the probes aligned to human genes were taken in account. The normalized enrichment score (NES) and q.value are indicated. **C** Pearson correlation analysis between the amount of Nestin staining in the tumor tissue at endpoint and the tumor growth velocity (GLuc increase divided by the number of days). 11 mice in total; *p* = 0,0002; Pearson test. **D** Overall survival and progression-free interval prognostic index estimation in TCGA-GBM (only IDH-wt patients). CL3 signature was used to stratify patients. Age and gender were not different in the two groups. **E** CL3 signature enrichment analysis in paired GBM patient’ tissues from the longitudinal GLASS consortium dataset. GSEA was performed on the paired tissues from each patient (first vs second surgery). Patients were stratified in 2 classes: with TMZ + IR after the first surgery or with no therapy. When q.val(NES) was less than 0.25 was considered to be “no change” (81 patients were analyzed in total). Distribution of patients and corresponding table. **F** Reactome over-representation analysis for the CL3 geneset and its interactors. **G** Gene Ontology analysis for the CL3 geneset in the KEGG or HALLMARKS datasets. **H** Biological functions by Ingenuity Pathway Analysis using the differentially expressed genes in CL3 vs the rest of the cells in MGG4 gliomaspheres from Fig. 1B. **I** Over-representation analysis for CL3 signature. The significantly over-represented pathways are colored and specified (performed with DecoPath). Schematics created with BioRender.com.

Next, to determine whether therapy-induced reprogramming occurs in vivo, we studied orthotopic MGG4 tumors irradiated or treated with TMZ. To obtain a reliable in vivo model, we implanted GLuc-secreting MGG4 cells intracranially and monitored tumor growth with peripheral blood GLuc for approximately three months^20^. Treatment was performed at size-match (predefined threshold of GLuc assay) with 10 Gy whole-brain irradiation or 10 mg/kg i.p. TMZ. As expected, both treatments affected the tumor growth and cell density (Supplementary Fig. 4A, B). IHC and digital pathology allowed us to precisely quantify NesHI cells within the tumors seven days after treatment. Notably, both the percentage of NesHI cells and the amount of Nestin per cell increased in IR- or TMZ-treated MGG4 tumors (Fig. 3A, B, Supplementary Fig. 4C). In addition, GSEA on the mRNA-sequencing of post-therapy compared to naive tumors demonstrated that the CL3 geneset was enriched after treatment, thus cross-validating our results (Fig. 3A, B Supplementary Fig. 4D).

Interestingly, MGG4 tumor growth rate was positively correlated with the number of NesHI cells in the tumor (Fig. 3C). This suggests that Nestin levels and CL3 signature expression are predictive indicators of tumor aggressiveness. Therefore, we used The Cancer Genome Atlas (TCGA-GBM) dataset with the CL3 geneset and found that the CL3 signature was linked to poor GBM prognosis, specifically for progression-free interval (HR:13.7) and overall survival (HR = 21.8) (Fig. 3D, Supplementary Fig. 4E, F). We then tested whether the CL3 signature was enriched in patients who had recently received IR and TMZ. To do so, we analyzed The Glioma Longitudinal AnalySiS (GLASS) consortium dataset^33^. Interestingly, the patients who received IR + TMZ between the first and the second resection seemed to experience an enrichment in the CL3 signature compared to the patients who did not receive IR + TMZ between the two resections (Fig. 3E).

Finally, to understand the CL3 phenotype and its possible biological role, we examined the features of CL3. Reactome, Gene ontology (GO) and Ingenuity pathway analyses (IPA) of the CL3 signature revealed several molecular features, such as ECM-receptor and laminin interaction, focal adhesions and elastic fibers, post-transcriptional phosphorylation and SEMA4A, FGFR1, Rho GTPase and YAP1 signaling (Fig. 3F, G) as well as some cellular functions, such as inhibition of mortality, activation of cell survival and migration (Fig. 3H, I, Supplementary Fig. 4G). Notably, over-representation analysis (ORA) also highlighted the potential involvement of senescence and cytokine signaling (Fig. 3H).

Altogether, we discovered a cell state in GBM cells, already present in the naïve population but strongly induced by TMZ or IR treatment via phenotypic reprogramming. Moreover, we found that monitoring Nestin expression may be instrumental in following this GBM cell state.

### The naïve CL3/NesHI cell state is slow-cycling, senescent-like, reversible and resistant to therapy

**Fig. 4:**
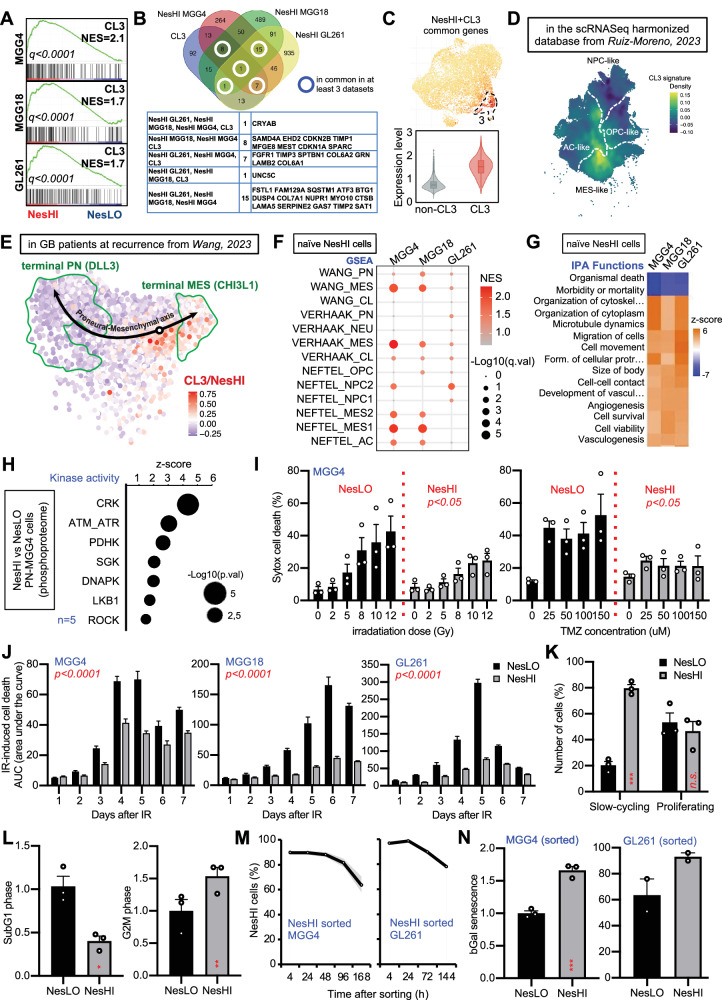
The CL3/NesHI state is already present in naïve GBM cells and is reversible, slow-cycling, senescent-like and resistant to therapy. **A** GSEA plot of the CL3 signature between NesLO and NesHI cells in Nestin^P^-dTomato MGG4, MGG18 and GL261 sorted-cells. Normalized enrichment score (NES) and q.value are indicated. **B** Genes in common in CL3/NesHI genesets. **C** Feature plot and violin graph of NesHI common genes, i.e. the genes commonly upregulated in at least 3 genesets, among NesHI MGG4, MGG18, GL261 and CL3 (see Supplementary Fig. 5B) (2 independent experiments and 2 time-points per experiment). **D** Feature plot for CL3/NesHI signature (32 genes) in the database from Ruiz-Moreno, 2023. **E** Feature plot for CL3/NesHI signature (32 genes) in the Proneural-Mesenchymal axis RNA-velocity from Wang et al. 2023. **F** Bubble plot of GBM states genesets in NesHI MGG4, MGG18 and GL261 cells. **G** Biological functions by IPA in NesHI MGG4, MGG18 and GL261 cells. **H** Kinase enrichment analysis (KEA) on phosphoproteome of NesHI vs NesLO-sorted MGG4 GBM cells (*n* = 5; z-score is indicator of the kinase activity estimated; significant kinase groups are plotted). Volcano plot of the phosphoproteome in Supplementary Fig. 8F.** I** Cell death analysis in NesLO and NesHI cell populations in Nestin^P^-dTomato MGG4 cells upon IR or TMZ. Data are means ± SEM. (*n* = 3 independent experiments, two-way ANOVA, *p* value between NesLO and NesHI). **J** Area under the curve analysis of Sytox+ cells in MGG4, MGG18, and GL261 cells treated with 2, 5, 8, 10, 12 Gy overtime (1–7 days) by FACS. Data are means ± SEM (*n* = 3 independent experiments, two-way ANOVA, Turkey’s multiple comparison test, *p* value between NesLO and NesHI are shown). Dose-effect plots in Supplementary Fig. 9. **K** FACS analysis of CellTrace^TM^ dye dilution during cell division. Data are means ± SEM (*n* = 3 independent experiments; ns, non-significant; ***p < 0.001). **L** Bar plots showing decrease in SubG1 and increase in G2M cell cycle phases in NesHI cells compared to NesLO in Nestin^P^-dTomato MGG4 cells. Data are means ± SEM (*n* = 3; *p < 0,05; **p < 0.01; paired two-sided *t* test). **M** Decrease of NesHI cell population overtime in FACS-sorted NesHI MGG4 and NesHI GL261 cells by FACS. Data are means ± SEM (*n* = 3). **N** β-Gal senescence staining in FACS-sorted NesLO and NesHI MGG4 or GL261 cells. Data are means ± SEM (*n* = 3 independent experiments; ***p < 0.001; unpaired two-sided t-test).

Next, to gain further molecular insight into the naïve (untreated) CL3 cell state and to broaden our results we analyzed the transcriptome of the sorted NesHI cells in the MGG4, MGG18 patient-derived cell lines and the GL261 mouse cell line (Supplementary Fig. 5A). The NesHI upregulated genes were broadly different across the three cell lines (Supplementary Fig. 5B, Supplementary Data File 2) but coherent with the CL3 signature and overall characterized by similar functions (Supplementary Fig. 5C). Indeed, NesHI cells were all drastically enriched in CL3 signature (Fig. 4A), by validating the effectiveness of the Nestin^P^-dTomato tool to study the CL3 state transitions regardless of mutational and transcriptional landscapes. Moreover, the 32 genes commonly upregulated in at least three NesHI subpopulations and in all cell lines (Fig. 4B) coherently labeled cells from CL3 in the scRNA-Seq map (Fig. 4C, Supplementary Fig. 5D) and were prognostic for patients in the TCGA dataset (Supplementary Fig. 6, Supplementary Data File 3). Notably, *CDKN1A* (*p21*) and *CDKN2B* (*p15*) stood out among the 32 commonly upregulated genes which are important markers for cellular senescence as well as its signatures (Fig. 4B, Supplementary Fig. 5E).

NesHI transcriptome analyses allowed us to explore the features of this cell state in more depth and regardless of the mutational landscape, GBM subtype or species. In the GBM harmonized database, the 32-gene signature traced over the profile observed for the 150-gene CL3 at the borders between cell states (Fig. 4D). Thus, we interrogated a recent scRNA-Seq dataset of paired clinical material where RNA-velocity analysis helped to draw the proneural-to-mesenchymal transition (PMT) occurring upon therapy^34^. As expected, the CL3 cells appeared to be intermediate in the proneural-mesenchymal axis (Fig. 4E, Supplementary Fig. 7A). Furthermore, GSEA clearly showed that NesHI cells are more mesenchymal in PN cell lines, such as PN-MGG4, while they are more proneural in extreme MES cell lines, such as MES-GL261 (Fig. 4F), confirming the intermediate features of the NesHI/CL3 cells. Functionally, the Reactome showed similarities in NesHI cells, regardless of the transcriptomic and mutational landscape (Supplementary Fig. 7B) and IPA predicted uniform activation of cell survival, viability and migration regardless of the cell line (Fig. 4G), which was in line with the scRNA-seq data (Fig. 3H). Moreover, GSEA suggested senescence-like and slow-cycling features for NesHI-CL3 cells (Supplementary Fig. 5E and Supplementary Fig. 8A–C). As expected, in line with these results, the post-therapy MGG4 transcriptomes were enriched in CL3, slow-cycling and senescence-like signatures (Supplementary Fig. 8D). Notably, the NesHI cells seem to be coherently enriched in MES-imm and 118-GS^18,35^, two recently discovered scRNA-seq signatures important for immune-evasion and resistance to therapy, respectively (Supplementary Fig. 8E).

To obtain additional molecular insights, we analyzed the phosphoproteome of NesHI- and NesLO-sorted PN-MGG4 cells using Liquid Chromatography coupled to tandem Mass Spectrometry (LC-MS/MS) analysis. Interestingly, kinase activity calculated using the phosphoproteome in NesHI cells indicated pronounced basal activation of all key DDR pathways, such as ATM, ATR and DNA-PK (Fig. 4H, Supplementary Fig. 8F). This is consistent with the senescence features of CL3/NesHI cells, as chronic activation of DDR induces senescence^36^.

To validate these intriguing findings regarding DDR and survival, we used our fluorescent reporter to select or sort CL3 cells and functionally assess this cell state. Therefore, we tested resistance to therapy by challenging Nestin^P^-dTomato-transduced MGG4, MGG18 and GL261 gliomaspheres with increasing doses of IR or TMZ. Even if naïve (untreated) cell survival was not modulated in some cell lines, NesHI cells were consistently more resistant to therapy than NesLO cells, as shown by the area under the curve (AUC) of the dose-response plots (Fig. 4I, J, Supplementary Fig. 9). They also resulted in slower cycling than NesLO, as shown by the CellTrace dye assay (Fig. 4K, Supplementary Fig. 10A, B). The Edu/PI cell cycle analysis demonstrated that NesHI cells were characterized by more cells in the G2M phase and fewer cells in the SubG1 phase, which is an indicator of cell death (Fig. 4L, Supplementary Fig. 10C). We then established that the CL3 cell state is partly reversible by measuring the NesHI percentage within a population of NesHI-sorted cells (Fig. 4M).

Finally, we investigated the mechanism of action underlying CL3 resistance. Nestin is also a GSC marker; therefore, we tested whether the resistance to therapy was due to the already demonstrated stemness features of NesHI cells^32^ or to the anti-apoptotic senescence, as suggested by some of our above-described results. Although undoubtedly GSC-like cells with retained intrinsic stem-like clonogenic features, NesHI cells did not show a higher clonogenic capability than NesLO (Supplementary Fig. 10D).

Cellular senescence has been shown in cancer both basally in untreated cells and as a reaction to stress^37^. Our findings on NesHI cells matched the definition of cancer cell senescence, as it makes NesHI cells more resistant to therapy and slow-cycling (Fig. 4I–K). Moreover, we observed the upregulation of senescence markers, such as p21/p15 upregulation, activation of senescent pathways (Supplementary Fig. 5E, Supplementary Fig. 8B, C) and DDR machinery (Fig. 4H), marked β-Gal senescence (Fig. 4N), and increased gamma-H2Ax foci (Supplementary Fig. 10E)^38^.

These results show that the CL3/NesHI cell state is intermediate in the PMT, resistant to therapy, slow-cycling, enriched in the G2M phase, stem- and senescent-like, partially reversible and with DDR machinery activation.

### VC-Resist state co-opts brain vasculature, which likewise induces cell state transition

**Fig. 5:**
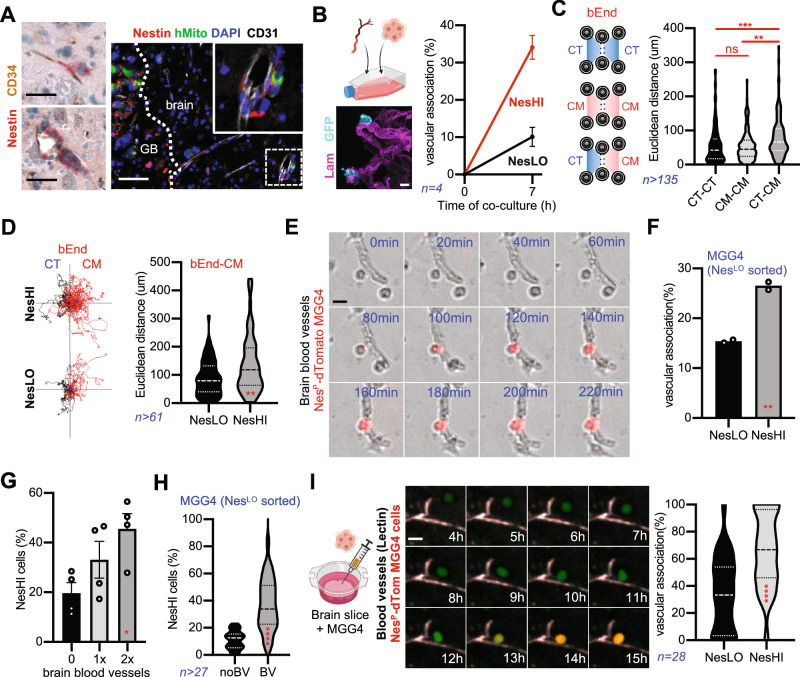
VC-Resist GBM state co-opts brain vasculature, which in turn induces cell reprogramming towards the VC-Resist state. **A**, (Left) Immunostaining of Nestin+ GBM cells (red) close to CD34+ blood vessels (brown) in the invasive front of intracranial MGG4 tumor sections. Similar results was seen in 4 mice. Scale bar, 10 μm. (Right) IF staining for Nestin, human mitochondria, CD31 (blood vessels) in MGG4-tumor-bearing mouse brain irradiated (10 Gy). Scale bar, 50 μm. **B**, (Left) Immunostaining of isolated brain blood vessel (laminin, red) and MGG4-GFP cells in the ex-vivo coculture model. Scale bar, 10 μm. (Right) Vascular association of Nestin^P^-dTomato MGG4 cells after 7 h of co-culture. Data are means ± SEM (*n* = 4 independent experiments; **p < 0.01; unpaired two-sided *t* test). **C** Ibidi chamber slide for chemotaxis of Nestin^P^-dTomato MGG4 cells to endothelial cells conditioned media (bEnd-CM). MGG4 cells directionality (Euclidean distance) in conditioned media (CM)-control (CT) in comparison to CT-CT and CM-CM conditions (*n* = 3; total number of cells quantified are >135; ns, non-significant; **p < 0.01; one-way ANOVA, Turkey’s multiple comparisons test). **D**, (Left) Trajectory plots of FACS-sorted NesHI and NesLO Nestin^P^-dTomato MGG4 cells in response to bEnd conditioned media (CM) vs control (CT) condition (*n* = 3). (Right) Directionality (Euclidean distance) of FACS-sorted NesHI and NesLO cells towards bEnd conditioned media (CM). Data are means ± SEM, *n* = 3, total cells quantified are >65; **p < 0.01; unpaired two-sided *t* test. **E** Time-lapse imaging of cell state transition of a NesLO MGG4 cell to NesHI when close to blood vessel. Scale bar, 10 μm. **F** Vascular association in NesLO-sorted MGG4 cells after 7 h of co-culture. Data are means ± SEM (*n* = 4 independent experiments; **p < 0.01; unpaired two-sided *t* test). **G** Percentage of NesHI cells after 7 h of co-culture with different amounts of brain blood vessels by FACS. Data are means ± SEM (*n* = 4 independent experiments; *p < 0.05; unpaired two-sided *t* test). **H** Percentage of cell state transitions in NesLO cells cultured with blood vessels by FACS. Data are means ± SEM (*n* = 2 independent experiments, total number of cells analyzed >27; ****p < 0.0001; unpaired two-sided *t* test). **I** (Left), Time-lapse confocal micrographs showing the NesLO-to-NesHI reprogramming when close to blood vessels (lectin) and MGG4-Nes-GFP cell in brain slice organotypic model. Scale bar, 20 μm. (Right**)**, Vascular association of Nestin^P^-dTomato MGG4 cells after 20 h of co-culture. Tracking of 28 cells (****p < 0.0001, unpaired two-sided *t* test). Schematics created with BioRender.com.

We then investigated the microenvironment in which the cell state described above was enriched. Interestingly, in our GBM models we noticed a peculiar accumulation of NesHI cells around normal blood vessels at the invasive front, also known as perivascular satellitosis (Fig. 5A, Supplementary Fig. 11A). This in vivo localization strongly suggests that NesHI cells might co-opt pre-existing blood vasculature, which is the active movement of tumor cells towards the pre-existing vasculature^20–22^. To test this hypothesis, we developed a specific assay for brain vessel co-option. We isolated intact pieces of mouse brain blood vessels with live components of the perivascular niche and co-cultured them with GBM cells (Fig. 5B, Supplementary Fig. 11B, C). We then quantified the vascular association of GBM cells after 7 h of co-culture as an indicator of brain vessel co-option. Notably, NesHI cells were significantly more associated with blood vessels than NesLO cells (Fig. 5B). This prompted us to test the chemotaxis of GBM cells towards brain endothelial cells (bEnd) or brain blood vessel-conditioned media. MGG4 cells were significantly attracted by bEnd- or blood vessel-conditioned media (bEnd-CM or BV-CM; Fig. 5C, Supplementary Fig. 11D, E) and the NesHI cells were faster and more directional towards the endothelial released factors than their NesLO counterparts (Fig. 5D, Supplementary Fig. 11F). Altogether, these findings demonstrate that the CL3/NesHI state intrinsically co-opts brain blood vessels; thus, we named this cell state VC-Resist.

We then tracked Nestin^P^-dTomato MGG4 cells co-cultured with brain blood vessels. Surprisingly, we noticed that several NesLO cells reprogrammed to NesHI in close proximity to the blood vessels (Fig. 5E, Supplementary Movie 1). Therefore we co-cultured the NesLO-sorted cells with blood vessels and quantified their vascular association. As expected, we found a high percentage of NesHI cells in contact with blood vessels (Fig. 5F); however, this may be caused by both reprogramming or vessel co-option. To separate these two effects, we quantified the percentage of NesHI cells when co-cultured with blood vessels. Mechanistically, both unsorted MGG4 cells and NesLO-sorted cells demonstrated that blood vessels induce reprogramming (Fig. 5G, H).

Next, we increased model complexity and examined the brain slice organotypic model. We seeded Nestin^P^-dTomato MGG4 cells on brain slices labeled with fluorescent lectin to detect blood vessels. We then accurately tracked their trajectories and reporter’s fluorescence (Supplementary Fig. 12A). At 1 h of co-culture the NesHI cells were approximately 30-40 um from the blood vessels and were thus randomly sparse, and over time they gradually moved closer to the blood vessels (Fig. 5I, Supplementary Fig. 12B, Supplementary Movie 2). Quantification of the localization of MGG4 cells after 20 h of co-culture showed a striking prevalence of NesHI cells that move closer to blood vessels (Fig. 5I, Supplementary Fig. 12C).

### VC-resist cell state co-opts brain vasculature and chemoradiation induces in vivo vessel co-option

**Fig. 6:**
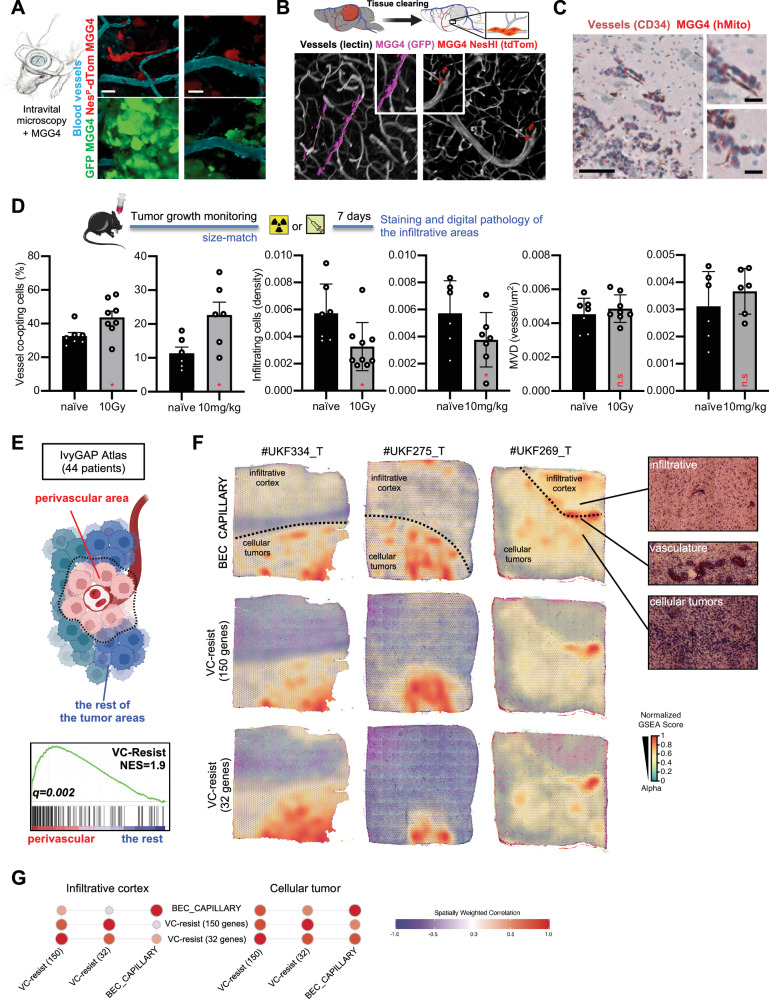
Preclinical and clinical validation of the VC-Resist cell state post-therapy increase and localization. **A** Intravital multiphoton imaging of GFP and Nestin^P^-dTomato MGG4 cells. Blood vessels are visualized using CascadeBlue dextran. Similar results was seen in 4 mice. **B** 3D confocal imaging of cleared thick brain slices from MGG4 tumor-bearing mouse brains. Vessel co-opting GFP+ (magenta) and NesHI (dTomato, red) GBM cells at the leading edge of the tumor. **C** Vessel co-option at the invasive fronts of MGG4 tumors. GBM cells (hMito; red) close to blood vessels (CD34; brown). Similar results was seen in 10 mice. Scale bar, 50 μm **D** Percentage of vessel co-opting GBM cells in the infiltrative front or the density of infiltrative GBM cells in MGG4-tumor-beraring mice at 7 days post-irradiation (10 Gy) or post-TMZ-treatment (10 mg/kg). Data are means ± SEM; *n* = 7 (naïve), *n* = 8 (10 Gy), *n* = 6 (naïve, DMSO), *n* = 6 (TMZ, 10 mg/kg); *p < 0,05; unpaired two-sided *t* test. **E** GSEA analysis for the VC-Resist signature in the IvyGAP atlas. Perivascular region is named microvascular proliferation in the atlas (44 patients). The normalized enrichment score (NES) and q.value are indicated. **F** Surface plot of spatially resolved expression of VC-Resist (150 genes), VC-Resist (32 genes) and BEC_Capillary signatures of 3 patients. Infiltrative cortex and cellular tumor are delimited. Normalized GSEA score is color-coded. **G** Bubble plot of spatially weighted correlations across VC-Resist (150 genes), VC-Resist (32 genes) and BEC_Capillary signatures in *n* = 3 patients in both infiltrative cortex and cellular tumor. Spatially weighted correlation is color-coded. Schematics created with BioRender.com.

These intriguing results prompted us to investigate the behavior of NesHI cells in an orthotopic environment. Intravital microscopy of Nestin^P^-dTomato MGG4 tumors implanted in mouse brains showed that the VC-Resist cells were preferentially located in the proximity of Dextran-labeled blood vessels and extended protrusions towards them (Fig. 6A).

Since IR or TMZ induced an enrichment in the VC-Resist cell state, we then tested whether chemoradiation resulted in an increase in vessel co-option at the infiltrative areas of MGG4 tumor models. The invasive front of treated MGG4 tumors was often characterized by perivascular satellitosis, as shown by 2D IHC and 3D cleared deep imaging (Fig. 6B, C, Supplementary Fig. 12D). Digital pathology of hMito/CD34 staining showed an evident intensification in vascular satellitosis and demonstrated a significant increase in the percentage of vessel co-opted cells in the infiltrative areas after IR or TMZ with no increase in overall infiltrating cells (Fig. 6D). To ensure that no neo-angiogenesis occurred in the infiltrative areas, we quantified the microvessel density (Fig. 6D), thus confirming that vessel co-option occurred towards pre-existing brain blood vessels as we previously described^20^.

To determine the relevance of these findings in patients, we first investigated the IvyGAP patients’ atlas of GBM regional transcriptomes^39^. Our analysis confirmed that VC-Resist geneset was enriched in perivascular regions (called microvascular proliferation in the atlas) compared to the rest of the tumor areas (Fig. 6E). We then examined the localization of the VC-resist specifically in the infiltrative area with a higher resolution of spatial transcriptomics in patients with GBM. To do so, we tested the spatial correlation between our two VC-resist genesets—the CL3 150 genes and the set of the 32 common genes (Supplementary Data File 2)—and a recently published signature for brain capillaries^40^. We took advantage of a recently published framework for GBM patient spatial transcriptomics^41^ and noticed an evident spatial correlation of the VC-Resist signatures with brain capillaries in both infiltrative zones and cellular tumor (Fig. 6F, G). Moreover, this analysis allowed us to investigate the spatial correlation of VC-Resist signatures with GBM classifier genesets, thus confirming that VC-Resist cell state spreads between the AC-like and the MES-like states in the infiltrative areas as seen in the ssGSEA in Fig. 1F, while it coincides with MES-like in the tumor core (Supplementary Fig. 12E).

Although further cases of GBM are needed to confirm this finding in patients, these findings combined with the others above collectively suggest that the naïve and treated VC-Resist cell state co-opts brain vasculature and that brain vessels induce cell state transition of NesLO cells towards the VC-Resist phenotype in vitro and in vivo.

### Angiocrine factors induce reprogramming, resistance to therapy and partial proneural-to-mesenchymal transition

**Fig. 7:**
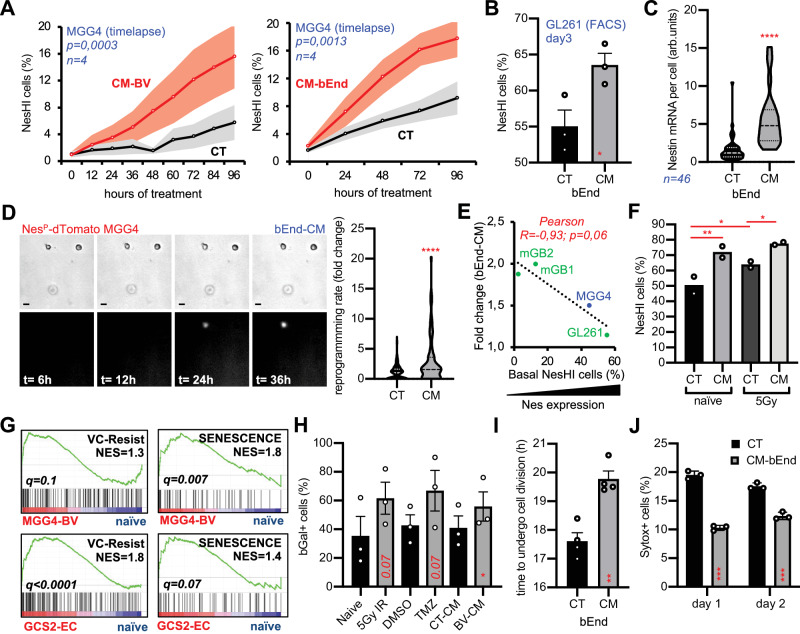
Angiocrine factors induce VC-Resist state transition, cell survival and partial proneural-to-mesenchymal transition. **A** Nestin expression in Nestin^P^-dTomato MGG4 cells in presence of conditioned media from blood vessels (CM-BV) or conditioned media from brain endothelial cells (CM-bEnd) using time-lapse imaging. Data are means ± SEM (*n* = 4 independent experiments; *p* = 0.0003; Spearman correlation). **B** Enrichment of GL261 NesHI cell population when cultured with bEnd-CM or control (CT) media by FACS. Data are means ± SEM (*n* = 3 independent experiments; *p* = 0.0016; paired two-side *t* test). **C** Nestin mRNA per cell in Nestin^P^-dTomato MGG4 cells in the presence of bEnd.3 conditioned media (CM) or control media (CT). Total number of cells=46, unpaired two-sided *t* test. **D** (Left) Real-time micrographs of Nestin^P^-dTomato MGG4 cells embedded in agarose gel showing the induced NesLO-to-NesHI transitions. (Right) Quantification of the reprogramming rate in Nestin^P^-dTomato MGG4 cells pre-conditioned in CT or CM for 3 days (*n* = 76; ****p < 0.0001; unpaired two-sided *t* test). **E** Pearson correlation analysis of basal Nestin expression and fold change (FC) of Nestin expression in presence of conditioned media (bEnd-CM) in 4 GBM cell lines. *X-*axis: 1/basal CT values for Nestin expression by RT-PCR, *Y-*axis: Nestin expression FC with bEnd-CM. Patient-derived (blue) mouse (green) cell lines (*n* = 3; R = 0,88; *p* = 0,001; Pearson test). **F** Enrichment of MGG4 NesHI cell population in the presence of CM-bEnd (CM) both with (5 Gy) and without IR analyzed at day5 by FACS. Data are means (*n* = 2 independent experiments; *p < 0.05; **p < 0.01; two-way ANOVA, Turkey’s multiple comparisons test). **G** (Top), GSEA plots of the VC-Resist signature and senescence geneset (FRIDMAN_SENESCENCE_UP) in MGG4 co-cultured with blood vessels (Bottom), or GSC2 co-cultured with endothelial cells. The normalized enrichment score (NES) and q.value are indicated. **H** β-Gal senescence in Nestin^P^-dTomato MGG4 cells after treatments (5 Gy of IR, 50 μM TMZ) or stimulated with CM-BV. Data are means ± SEM (*n* = 3 independent experiments; *p < 0.05; unpaired two-sided *t* test). **I** FACS analysis of CellTrace^TM^ dye dilution during cell division in CT or CM-bEnd cells. Time to undergo cell division was calculated based on the mean fluorescent intensity values. Data are means ± SEM (*n* = 4, **p < 0.01). **J** Cell death (percentage of Sytox+ cells) in Nestin^P^-dTomato MGG4 cells conditioned with bEnd.3 conditioned media (CM) or control (CT). Data are means ± SEM (*n* = 3 independent experiments; ***p < 0.001; unpaired two-sided *t* test).

In other tissues and contexts, endothelial cells—and more generally blood vessels—are known to release angiocrine factors that protect cells and induce survival^25,42,43^. However, the mechanism by which angiocrine factors modulate GBM cell state transition and resistance to therapy remains unclear. We therefore studied whether endothelial or blood vessel-conditioned media could induce reprogramming towards the VC-Resist cell state. As suggested by Fig. 5 and previous reports^25,44^, media conditioned by brain blood vessels or bEnd strongly reprogrammed MGG4 and GL261 cells, thus gradually increasing VC-Resist cells. This finding was confirmed by 2D and 3D time-lapse, FACS analysis, qPCR, and in situ single-cell RNA quantification (Fig. 7A–D, Supplementary Fig. 13A, B). Notably, angiocrine-induced reprogramming appeared to be even greater than the therapy-induced reprogramming shown in Figs. 1, 2. Also in this case, the impact on reprogramming was larger in cell lines with lower basal levels of Nestin (Fig. 7E). Notably, the effect of bEnd-CM on the VC-Resist reprogramming was even more profound than that of radiotherapy (Fig. 7F).

Next, we examined MGG4 cells when co-cultured with blood vessels or endothelial cells to verify the VC-Resist cell state transition at the transcriptomic level using RNA-seq profiling (Supplementary Fig. 13C, Supplementary Data File 4). The VC-Resist signature was enriched in PN-MGG4 cells co-cultured with blood vessel or in the MES-GCS2 GBM cells plated on endothelial cells (Fig. 7G). Moreover, angiocrine-induced reprogramming towards the VC-Resist state stimulated pathways of senescence (Fig. 7G) as well as G-STEM and MES-imm single-cell signatures (Supplementary Fig. 13D)^35,45^. We then validated these interesting transcriptomic results using functional assays. The ß-Gal assay demonstrated an increase in cellular senescence in CM-treated MGG4 cells, even more profoundly than that in therapy-treated MGG4 cells (Fig. 7H). Similarly, in line with the cell cycle NesHI results shown in Fig. 4, the CM-treated cells were enriched in the G2M phase and reduced in SubG1 dying cells (Supplementary Fig. 13E). Additionally, the CellTrace Proliferation assay showed that the CM-induced MGG4 cells were more slow-cycling/quiescent than their counterparts (Fig. 7I, Supplementary Fig. 12F). Altogether, these features made the CM-stimulated MGG4 cells basally more resistant (Supplementary Fig. 13G) as well as more resistant to therapy, as shown by the Sytox analysis (Fig. 7J) with an increase in the G2M cell phase and a decrease in SubG1 (Supplementary Fig. 13H).

**Fig. 8:**
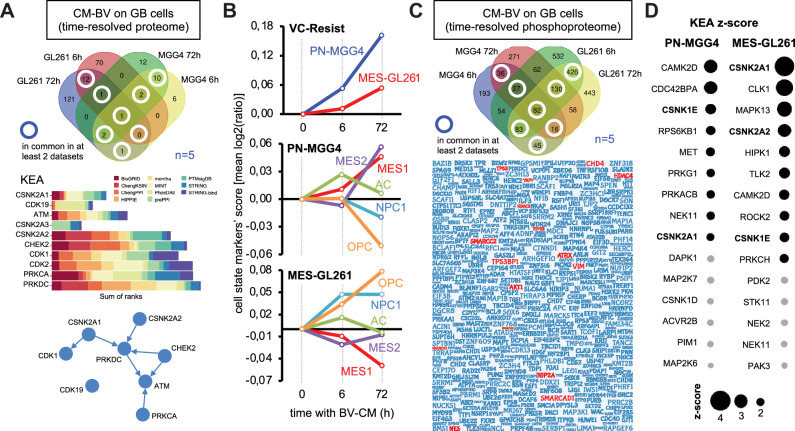
Time-resolved proteome/phosphoproteome of the angiocrine-induced VC-Resist state transition unveils partial proneural-to-mesenchymal transition. **A** Upregulated proteins in common between the patient-derived PN-MGG4 and the mouse MES-GL261 GBM cells treated for 6 or 72 h with control or blood vessel conditioned media (CM-BV) (*n* = 5 independent experiments). (Top) Venn graph of the common genes. (Middle) Kinase enrichment analysis (KEA) of the common proteins using the KEA3 web-based platform. Lower is the combined score, more probable is the activity of the kinase. (Bottom) Network among the top proteins in the KEA. Volcano plots are in Supplementary Fig. 14A. **B** Temporal proteomic profiling of cell plasticity in GBM exposed to blood vessel conditioned media (CM-BV). *Y-*axes are means of all log2(fold change) between CT and CM-BV for Neftel’ cell states (20–30 markers per GBM classifier) or VC-Resist (32 proteins), excluding the infinite values. **C** More phosphorylated proteins in common between the patient-derived PN-MGG4 and the mouse MES-GL261 GBM cells treated for 6 or 72 h with control (CT-CM) or blood vessel conditioned media (BV-CM). (Top) Venn graph of the common genes. (Bottom) Word cloud plot for the commonly hyper-phosphorylated proteins. The largest fonts represent proteins that are present in all four datasets, while the smallest fonts are for proteins shared in just 2 datasets (from different cell lines). Volcano plots are in Supplementary Fig. 14C. **D** Kinase enrichment analysis (KEA) on phosphoproteome in the patient-derived PN-MGG4 and the mouse MES-GL261 GBM cells treated for 72 h with control (CT-CM) or blood vessel conditioned media (BV-CM) (*n* = 5 independent experiments; z-score is indicator of the kinase activity estimated; significant in black and not-significant in gray).

Next, to obtain further molecular insight into the VC-Resist transition dynamics, we performed a time-resolved proteome analysis of two opposite GBM cell lines (PN-MGG4 and MES-GL261) treated with blood vessel-conditioned medium (BV-CM) (Supplementary Fig. 14A). To ensure the solidity of the data, we carried out this analysis in quintuplicates. The results of the kinase enrichment analysis of the upregulated proteins in both GBM cell lines at 6 h or 72 h following treatment with BV-CM indicate the common activation of the DDR machinery, particularly the kinases ATM, DNA-PK (also known as PRKDC), and CHEK2 (Fig. 8A), which is in line with the activation detected in CL3/NesHI cells (Fig. 4H). Moreover, multiple common upregulated proteins were present in the senescence-associated secretory phenotype (SASP) Atlas previously published (Supplementary Fig. 14B)^46^. These time-resolved proteome datasets were particularly useful to test whether VC-Resist was enriched upon BV-CM at the protein level and to understand whether GBM cells exposed to BV-CM were induced to partial PMT or partial MPT, depending on the original GBM cell line status, as suggested at the transcriptomic point of view (Fig. 4E, F). First, we tested whether the VC-Resist gene set (32 genes) from Fig. 4B was suitable for interrogating proteome datasets. Our results confirmed that BV-CM induces a gradual enrichment of VC-Resist in both PN-MGG4 and MES-GL261 cells, regardless of their transcriptomic and mutational landscape (Fig. 8B). Moreover, using as many Neftel state markers as possible (20–30 per GBM classifier), we noticed that BV-CM induced a progressive but partial PMT in PN-GBM cells (among others, with enrichment in VIM, A2M, and CDKN1A), while it induced a partial mesenchymal-to-proneural transition (MPT) in MES-GBM cells (for example, upregulation of OLIG1/2 and CHD7, but strong downregulation of SOX4) (Fig. 8B, Supplementary Data File 5), which is in line with the GSEA of CL3/NesHI sorted cells (Fig. 4F). This clearly indicates that the VC-Resist state induced by BV-CM is intermediate in the GBM proneural-mesenchymal axis, thus cross-validating our transcriptomic results from Fig. 4E, F.

Finally, we searched for common signaling hotspots using phosphoproteomic analysis. Multiple proteins with log2(FC) higher than 0.8 were common between the two GBM cell lines (PN-MGG4 and MES-GL261). Among the 557 proteins with increased phosphorylation in BV-CM GBM cells, SMARCs, HDAC2/4, AKT1, YAP1, NES, VIM, TOP2A/B, CHD4, ATRX, TP53 and TP53BP1 popped up as interesting hits (Fig. 8C, Supplementary Fig. 14C, Supplementary Data File 5). Kinase activity analysis of the common proteins that were differentially phosphorylated revealed ATM and ATR activation and possible CSNK1E (also known as CK1e) and CSNK2a1/2 (collectively named CK2) signaling (Fig. 8D, Supplementary Fig. 15A). Notably, CK1e and YAP1 involvement, as well as the senescence and reprogramming features of VC-Resist and BV-CM, prompted us to further investigate YAP1, since it is known to be phosphorylated by CK1e^47^ and is important for these cellular functions in other models.

### Therapy- and vascular-induced reprogramming is driven by FGFR1-YAP1 axis’ activation

**Fig. 9:**
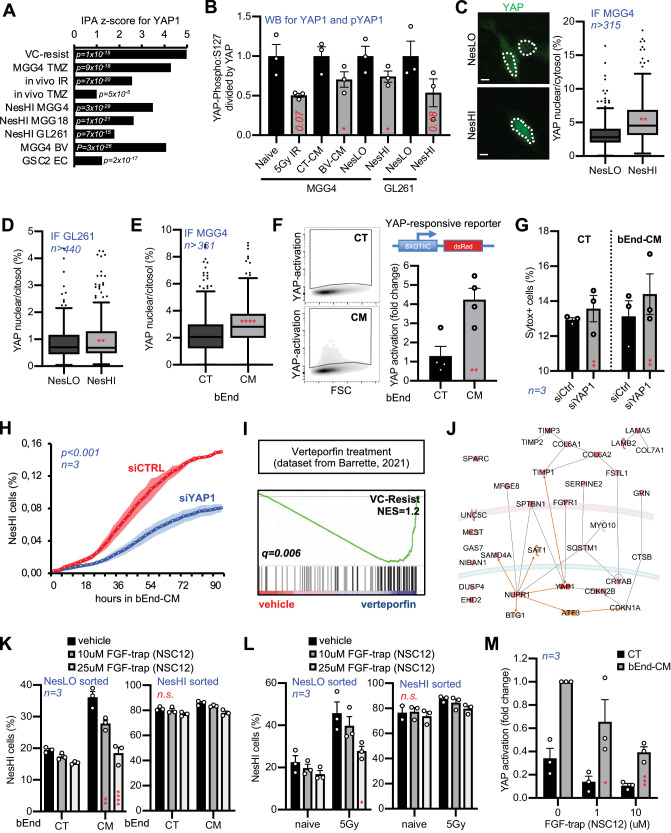
Therapy- and vascular-induced VC-Resist state transition is driven by FGFR1-YAP1 activation. **A** YAP1 Ingenuity Pathway Analysis (IPA) z-scores for all conditions and cell lines with corresponding *p* value. **B** Level of YAP1 S127-phosphorylation. Quantification of immunoblot using YAP and p-YAP antibodies for YAP activation in VC-Resist state transitions. Data are means ± SEM (*n* = 3 independent experiments; *p < 0.05; unpaired two-sided *t* test). Quantification of YAP localization in FACS-sorted MGG4 (**C**), GL261 (**D**) NesLO or NesHI cells or in MGG4 bEnd-conditioned (CM) or control (CT) media (**E**) (*n* = 326 cells for MGG4 NesLO, 315 cells for MGG4 NesHI; 343 cells for GL261 NesLO; 316 cells for GL261 NesHI; 377 cells for CT; 361 cells for CM; 3 independent experiments; unpaired two-sided *t* test). **F**(Left) FACS plots of YAP-activation (dsRed) in MGG4 cells treated with bEnd conditioned media (bEnd-CM) vs control (CTRL). (Top right) YAP-responsive reporter lentiviral construct. (Bottom right) YAP activation in MGG4 cells treated with blood vessel conditioned media (bEnd-CM) vs control (CTRL). Data are means ± SEM (*n* = 4 independent experiments; *p < 0.05; unpaired two-sided *t* test). **G** Inhibition of the BV-CM induced enrichment of NesHI when Nestin^P^-dTomato MGG4 cells are silenced for YAP1 (siYAP1) in comparison with scramble (siCTRL) by FACS. Data are means ± SEM (*n* = 3 independent experiments; **p < 0.01; paired two-sided *t* test). **H** Nestin expression in Nestin^P^-dTomato MGG4 cells silenced for YAP1 (siYAP1) or scramble (siCTRL) in presence of conditioned media from blood vessels using live-cell imaging. Data are means ± SEM (*n* = 3 independent experiments; *p* < 0.001; Spearman correlation). **I** GSEA plot of the VC-Resist signature in verteporfin-treated GBM cells vs control from Barrette et al., 2021. **J** YAP activation is in silico predicted by using the Molecular Activity Predictor with the genes from VC-Resist signature. **K**, **L** The FGF-trap compound NSC12 inhibits the bEnd conditioned media (bEnd-CM) or the irradiation-induced reprogramming, while it does not modify the maintenance of NesHI state. Data are means ± SEM (*n* = 3 independent experiments; *p < 0.05; **p < 0.01 vs the CM/vehicle condition; paired two-sided *t* test). **M** The FGF-trap inhibitor NSC12 blocks the bEnd-CM-induced YAP-responsive reporter activation. YAP activation in MGG4 cells treated with conditioned media (bEnd-CM) vs control (CT) (*n* = 3 independent experiments; *p < 0.05; ***p < 0.001 vs the respective CM/vehicle condition; paired two-sided *t* test).

We then investigated the molecular regulators of the transition by searching for transcriptional programs in common across the three studied settings in PN-MGG4 cells. Interestingly, the differentially expressed genes and IPA-predicted biological functions were coherent across the analysed settings (Supplementary Fig. 15B), suggesting that a common transcriptional program is activated during VC-Resist transition. Moreover, we identified a cascade of upstream transcriptional regulators that can explain the observed gene expression changes in VC-Resist reprogramming using in silico IPA. We listed the common transcription factors (TF) predicted to be activated by IPA and discovered that most of them were directly or indirectly linked to the YAP/TAZ pathway (Fig. 9A, Supplementary Fig. 15C, Supplementary Data File 6), which is consistent with the results shown in Fig. 7. Notably, this occurred regardless of the genetic landscape or the transcriptional subtype of the cell lines.

IPA predictions and phosphoproteome results prompted us to investigate whether YAP1 was differentially phosphorylated in the three settings. Notably, S127 phosphorylation of YAP was reduced in NesHI MGG4 or GL261 cells as well as in IR-treated or CM-stimulated MGG4 cells (Fig. 9B, Supplementary Fig. 15D). It is known that S127 phosphorylation induces YAP cytoplasmic retention and inhibits its activation^48^, thus confirming the potential involvement of the YAP pathway in the transition to the VC-Resist cell state. To further verify the activation of YAP in the VC-Resist state transition, we quantified YAP nuclear localization in NesHI MGG4 and GL261 cells as well as in CM-treated MGG4. YAP localized in the nucleus more in the NesHI and in CM-treated cells than in their counterparts (Fig. 9C–E). We then used a published fluorescent reporter of YAP activation^49^ and confirmed that endothelial CM induces YAP activation (Fig. 9F).

Finally, to mechanistically test the involvement of YAP in VC-Resist reprogramming we silenced YAP1 in GBM cells induced to reprogram towards VC-Resist cell state. YAP1 silencing dampened BV-CM-induced reprogramming without inducing cell death, as shown by FACS analysis and real-time imaging (Fig. 9G, H, Supplementary Fig. 15F). Moreover, GSEA for the VC-Resist geneset of a recently published RNA-seq dataset of three GBM preclinical models treated with verteporfin—a well-recognized and clinically approved inhibitor of YAP^50^ – indicated that therapeutic inhibition of the YAP/TAZ pathway strongly reduces VC-Resist cell state (Fig. 9I).

Finally, we decided to in silico validate the potential activation and upstream regulator/s of YAP1 using the IPA molecule activity predictor (MAP) tool. Using transcriptomic measurements of the 32 common genes in NesHI VC-Resist cells and the curated Ingenuity Knowledge Base, IPA predicted strong activation of YAP1 in NesHI VC-Resist cells (Fig. 9J).

Because YAP1 is at the center of many signaling cascades, it remains difficult to target. Therefore, we decided to search for upstream regulators of VC-Resist specific YAP1 activation. Notably, FGFR1 seemed to be the only membrane receptor in the geneset directly linked with YAP1, so we decided to target FGF signaling to inhibit YAP1-driven reprogramming to VC-Resist. NSC12, an FGF-trap small molecule, blocked the bEnd-CM and IR-induced VC-Resist transition in a dose-dependent manner (Fig. 9K, L, Supplementary Fig. 15G) without inducing important cell death in MGG4 cells (Supplementary Fig. 15H). Interestingly, this was evident in unsorted and NesLO-sorted cells, but not in NesHI GBM cells, suggesting that FGF signaling is key in the transition but not in the maintenance of the VC-Resist cell state. Finally, to confirm that the NSC12 effects were due to YAP1 signaling, we tested the fluorescent reporter of YAP activation, as shown in Fig. 8F, and demonstrated that targeting FGF signaling reduced YAP1 activation in bEnd-CM-treated GBM cells (Fig. 9M).

Altogether, these findings revealed that the VC-Resist state transition is mechanistically driven by the FGFR1-YAP1 axis.

## Discussion

**Fig. 10:**
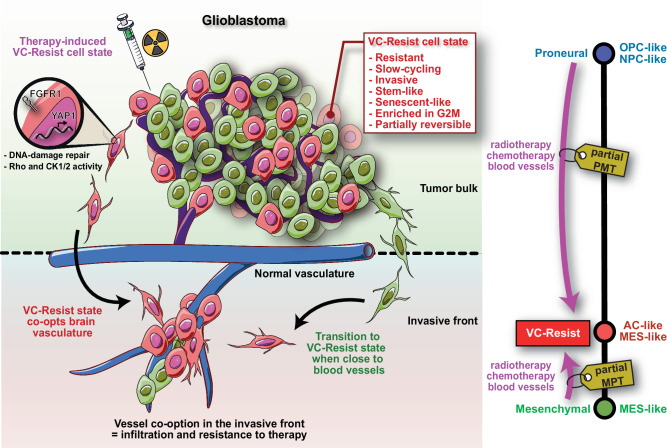
Here we show a cell state called VC-Resist that, even if already present in naïve tumors at different levels, is strongly induced by chemoradiation and angiocrine factors from the brain blood vessels. The VC-Resist cells are intermediate in the PMT and are highly resistant to therapy, vessel co-opting, senescent-like and slow-cycling. Considering our discoveries, we propose a model wherein chemoradiation leads to vessel co-option and resistance to therapy via reprogramming of GBM cells into the VC-Resist cell state. This creates a self-perpetuating cycle, as increased resistance and vessel co-option contribute to the recurrence of GBM.

The mechanism by which therapy alters cancer cell transcriptional states and their relationship with the GBM microenvironment remains poorly understood. Here, we show that therapy induces vessel co-option at the invasion front as well as resistance to therapy via reprogramming towards a functional cell state, which we named VC-Resist (Fig. 10). The VC-Resist cell state did not appear to be explicitly linked with any of the known GBM classifiers but predominantly comprised MES-like and AC-like states and was intermediate in the PMT. Notably, the absence of a substantial overlap with other previously reported cell states and its unique cellular functions suggests that VC-Resist is an innovative GBM cell state (Supplementary Fig. 16A). However, as expected, the VC-Resist state exhibited the highest similarity to the G-STEM and 118-GS signatures (Supplementary Fig. 16A), and some resemblance to the recently discovered p300-driven iGPC signature^51^. Indeed, it has been reported that the G-STEM state is highly dependent on YAP/TAZ pathway activation^45^ and the 118-GS geneset spots a slow-cycling and resistant cell state^18^; features that we highlighted in our own functional analyses of the VC-Resist cell state.

Interestingly, our transcriptomic and time-resolved proteomic results clearly place VC-Resist cells intermediate in the PN-MES axis – with partial PMT in PN cells and partial MPT in highly MES GBM cells—even though closer to the AC/MES-like terminal states. In addition, the VC-Resist location in the middle of the PN-MES axis was validated in a large scRNA-Seq dataset of recurrent GBM patients. Altogether, this indicates that VC-Resist is a hybrid state, as described in the partial EMT for other types of cancer^52,53^. In the GBM, the intermediate states in the PMT are unclear, and the data presented here are one of the first reports of pPMT and pMPT, and their link with therapy and the GBM microenvironment.

The possibility of tracking functional states using imaging is a recent frontier in the GBM field^54^. This allows us to track cell state localization, migration and reprogramming in time and space—even in specific environments as we did with blood vessel co-culture, brain slices or intravitally in the orthotopic mouse brain—thus opening new ways to understand cell plasticity. Here, we used Nestin expression as a reliable and solid marker to track the VC-Resist cell state. Notably, NesHI cells have already been explored in the GBM. Indeed, they have been demonstrated to be resistant and recurrent in a syngenetic GBM model^32^. These studies proposed a resistance mechanism for NesHI cells linked to their stemness, instead of a senescent-like and plastic status as we found. Moreover, the cause of the NesHI increase upon therapy was hypothesized to be due to the selection process of a resistant cell subpopulation^32^, while here we show that they are actively induced by therapy via cell plasticity.

We functionally investigated the features of NesHI/VC-Resist cells and found that they are particularly resistant to therapy, vessel co-opting, stem- and senescent-like, slow-cycling, reversible, and enriched in the G2M cell cycle phase. Notably, in a recent report NesHI cells have also been described as highly tumor microtube (TM)-connected, thus supplementing an additional mechanism of action for their demonstrated radioresistance^18^.

NesHI cells have been shown to have stem-like properties^55^. For this reason, we initially hypothesized that the mechanism of action behind their resistance to therapy was stemness and a potentially faster DNA-repair machinery, as shown in other GSCs^56,57^. Nevertheless, even if NesHI cells retained intrinsic stem-like clonogenicity features, in our gliomasphere stem-like experimental model we did not observe a higher clonogenicity of NesHI vs NesLO cells or a faster γ-H2AX foci repair, thus concluding that stemness – even if present—was not the cause of their resistance to therapy but their senescence-like features. However, senescence and stemness are strongly interrelated in multiple cancer models^55,57–59^, suggesting a non-exclusive senescence/stemness combination based on the cell plasticity observed under our experimental conditions.

Senescence has been described to be induced by therapeutic stress in GBM and many other cancers^60–65^. In this context, for the first time, we identified and characterized a senescent-like GBM cell state, and tracked the reprogramming that drives this cell state transition in vitro and in vivo using real-time imaging. The VC-Resist cell state is empowered by DDR machinery hyper-activation, as recently described in other resistant GBM cell states^66^, and is intrinsically present in naïve GBM cells at different levels, making the cells basally resistant to therapy. Interestingly, the VC-Resist senescent-like state appears partially reversible under the investigated experimental conditions and highly plastic GBM models. This is in line with several observations in other models and cancers^37,55,58^. Moreover, we surprisingly proved that angiocrine factors released from naïve blood vessels and endothelial cells strongly induce the cell state transition towards VC-Resist, regardless of the mutational landscape of GBM cells or their transcriptional subtype. This naïve TME-induced senescent-like status changes our way of seeing senescence as a phenomenon induced exclusively by stress and certainly opens stimulating questions on its role in GBM progression.

Altogether, our transcriptomic and functional findings indicate the slow-cycling VC-Resist as a highly infiltrative cell state, in line with the *go or grow* hypothesis^41,67–69^. Indeed, the specific localization of NesHI cells in proximity to blood vessels – not only in the core of the tumor as previously described^24^, but also in the invasive front – prompted us to investigate vessel co-option as a specialized infiltration strategy. VC-Resist cells showed a marked tendency to associate with blood vessels and to directionally move towards their angiocrine factors. In line with this, we also discovered that therapy with both IR and TMZ induced GBM vessel co-option, thus pointing out the NesHI/VC-Resist cells as the actors of perivascular satellitosis, a pathological hallmark of GBM^21,22^. The chemoradiation-induced stimulation of GBM vessel co-option has never been reported and is potentially a clinically-relevant issue, since on one side it increases the specialized infiltration of the brain parenchyma allowing cells to spread far from the tumor core, and on the other side it exposes cells to the perivascular microenvironment that protects them from the rest of the therapy protocol, as shown by us and others^25^. Another important issue raised by vessel co-opting cells is that they might not be detected by clinical imaging because the blood-brain barrier is often intact in the co-opted blood vessels^20^, thus evading gadolinium leakage that highlights tumor areas. This delineates a scenario where surgery does not remove the invasive vessel co-opting GBM cells that are intrinsically and extrinsically resistant to therapy due to reprogramming towards the VC-Resist cell state.

Moreover, it is worth mentioning that the cellular mechanism behind the vessel co-option described here for the VC-Resist GBM cells is different from the one previously described for Olig2/Wnt7+ GBM cells^20^. Indeed, if Olig2/Wnt7+ cells have been demonstrated to be individual-cell vessel co-opting in OPC-like GBM cells, the VC-Resist cells appear to co-opt vasculature as a collective stream of cells and are AC/MES-like; a molecular and physio-pathologic distinction previously suggested by us^22^. Notably, if vessel co-option was described in Olig2/Wnt7+ GBM cells as an intrinsic and acquired resistance mechanism to anti-angiogenesis^20^, here we discovered that vessel co-option in AC/MES-like cells is an intrinsic and acquired resistance mechanism to conventional therapy.

The molecular characterization of therapy-induced GBM cell states and their transitions, as performed here and in other reports^17,19,70–72^, has the potential to open the door to new therapeutic strategies aimed at inhibiting cancer cell plasticity and resistance which could greatly contribute to treatment improvement. To shed light on the molecular regulation of cell reprogramming, we identified the activated molecular cascade in the VC-Resist cell state. Thirty-two genes were found to be consistently upregulated in our NesHI VC-Resist cell datasets across all cell lines. The transcriptional profiles of VC-Resist cells appear to be homogeneous, with upregulation observed in several molecular classes: (**i**) several ECM components typically present in the vascular basement membrane (such as *LAMA5, LAMB2, SPARC, COL6A1-2*, and *TIMP1-2-3*)^73^; (**ii**) FGFR1, a receptor for FGFs (also secreted by endothelial cells) which plays a role in radioresistance^74^ and possibly in vessel co-option; and (**iii**) genes related to the senescent-like phenotype and stress-resistance (e.g. *CDKN2B, CDKN1A, CRYAB, ATF3, SERPINE2 and NUPR1*)^38,75,76^. Moreover, our proteome and phosphoproteome datasets revealed one of the molecular mechanisms behind the resistance to therapy of VC-Resist cells, with hyper-activation of the DDR machinery (Figs. 4H and 7K), and represents a large and refined source for future research on VC-Resist vulnerabilities. Notably, we showed that YAP1 activation is necessary for the VC-Resist cell state regardless of the mutational or transcriptional landscape of the cell or the experimental conditions that induce this cell state (IR, TMZ, naïve sorted cells, or blood vessels). YAP1 is undoubtedly at the center of multiple cell functions, such as epigenetics, senescence, and maintenance of stemness^45,77^. A recent report showed that radiation-induced YAP1 activation confers glioma radioresistance^64^, thus supporting our findings. However, cellular senescence has not been investigated. Even if probably not the only upstream regulator, here we show that FGFR signaling controls the blood vessel-induced YAP1 activation and VC-Resist transition, and this is consistent with the known pro-survival role of FGF in GBM^78^. Among others, it has been shown that Rho signaling is downstream of FGFR1-induced radioresistance in GBM cells^79,80^ and this is consistent with the pronounced Rho GTPase cycle activation predicted by the Reactome of the common phosphorylated proteins in BV-CM-treated GBM cells (Supplementary Fig. 15B).

The therapy-induced network of processes described here connects several hallmarks of cancer, such as resistance to cell death, phenotypic plasticity, non-mutational epigenetic reprogramming, senescence, access to vasculature and activation of invasion^37^; thus indicating why it may be central to GBM resistance and recurrence. This is a possible reason why we noticed a marked prognostic power for the VC-Resist (150) and VC-Resist (32) signatures as well as the enrichment of the VC-Resist signature in patients recently treated with IR and TMZ. Interestingly, our analyses of clinical retrospective data, also validated in a preclinical model, match an independent prospective small report that demonstrated Nestin as the unique GSC marker to be prognostic for the time of GBM recurrence^81^. Our findings suggest that cell plasticity during chemoradiotherapy directly induces resistance and aggressiveness in recurrent tumors.

To investigate the clinical relevance and broad application of the VC-Resist signature, we used a harmonized scRNA-Seq dataset and the Neftel map (Figs. 1F, G, 4D), overall survival and progression-free interval prognostic index estimation using TCGA dataset (Fig. 3D, Supplementary Fig. 6), longitudinal comparison of paired tissues from the GLASS Consortium (Fig. 3E), analysis of the scRNA-Seq PN-MES axis trajectory (Fig. 4E), and spatial transcriptomic analysis of large resections from patients with GBM (Fig. 6F, G, Supplementary Fig. 12E). We thus proved the existence, prognostic power, therapy-induced increase, and localization of the VC-Resist cell state directly in patients with GBM.

In conclusion, here we show a cell state called VC-Resist that, even if already present in naïve tumors at different levels, is strongly induced by chemoradiation and angiocrine factors from the brain blood vessels. The VC-Resist cells are intermediate in the PMT and are highly resistant to therapy, vessel co-opting, senescent-like and slow-cycling (Fig. 10). In light of our discoveries, we propose a model wherein chemoradiation leads to vessel co-option and resistance to therapy via reprogramming of GBM cells into the VC-Resist cell state. This creates a self-perpetuating cycle, as increased resistance and vessel co-option contribute to the recurrence of GBM.

## Methods

### Ethics

All animal care and treatment protocols complied with European legislation (no. 2010/63/UE) and national (French Ministry of Agriculture) guidelines for the use and ethical treatment of laboratory animals. Experimental procedures were specifically approved by the ethics committee of the Institut Curie CEEA-IC #118 (Authorization number APAFIS#24702-2020031815185853 v2 given by the National Authority) in compliance with the international guidelines.

### Cell lines and lentiviral transduction

The GBM patient-derived cell lines MGG4 and MGG18 were obtained from Dr. Wakimoto (Dept. of Neurosurgery, Massachusetts General Hospital, USA)^26^, the BG5, the BG7 and the NCH421k from Dr. Daubon (IBGC, Bordeaux, France), the BT18, BT27 and ZH305 from Dr. Le Joncour (Faculty of Medicine, University of Helsinki, Finland), the T98 from Dr. Dutreix (Institut Curie, Paris, France) and GSC2 were previously isolated by our team^82^. The mouse GL261 cell line was purchased from The Jackson Laboratory (Bar Harbor, USA) and the mGBM1 and mGBM2 were provided by Dr. Angel (DKFZ, Heidelberg, Germany). The BT18 and BT27 were maintained in neurospheres using Dulbecco’s Modified Eagle’s Medium (DMEM) containing F12,GlutaMAX^TM^ supplement (Gibco Thermo Fisher Scientific #10565018), B27^TM^ supplement serum free (GibcoTM, Thermo Fisher Scientific #17504044), 15 μM HEPES (GibcoTM, Thermo Fisher Scientific #15630080), 10 ng/ml bFGF (STEMCELL Technologies, #78003) and 20 ng/ml EGF (STEMCELL Technologies, #78006). All other GBM cells were maintained in neurospheres using NeuroCult basal medium with NeuroCult proliferation supplement (STEMCELL technologies, #05751), 20 ng/ml EGF, 20 ng/ml bFGF, 5ug/ml heparin (STEMCELL Technologies, #07980) and gentamycin (Sigma) (NC complete media) in low attachment flasks (Corning, #3814). All cell lines were repeatedly tested and were negative for mycoplasma using the Mycoplasma Detection Kit (MB Minerva Biolabs, #117048). Cells were authenticated and cultured for no more than 15 passages. Neurospheres were passaged when they reached a diameter of 100-150 μm or a high density using accutase (Thermo Fisher Scientific, A11105-01).

To follow the reprogramming of GBM cells upon treatment in real-time, MGG4, MGG18 and GL261 cells were infected with lentiviral particles of pLV-EGFP:T2A:Puro-Nestin>dTomato (Nestin^P^ dTomato). In this system, the tdTomato fluorescent protein was placed under the control of the Nestin promoter^30^, while the GFP fluorescent protein expression is controlled by the CMV promoter. Cells were then selected using 0,25ug/ml puromycin 48 h post transduction for 2 weeks.

The pLV-Bsd-EF1A>GLuc plasmid was built to measure the Gaussia luciferase activity (GLuc) released by the transduced cells implanted in vivo. This construct was generated to monitor tumor size, by measuring Gluc activity in the bloodstream of the mice^83^. MGG4 cells were infected with lentiviral particles and selected with 4ug/ml blasticidin 48 h post transduction for 2 weeks. The pLV-EGFP:T2A:Puro-Nestin>dTomato and pLV-Bsd-EF1A > (GLuc) were generated by VectorBuilder Inc. (Chicago IL, USA).

To follow YAP/TAZ activity in real-time, MGG4 cells were infected with lentiviral particles of pTRE 8XGTIIC DsRedDD (a gift from Joan Massague; Addgene plasmid #115798). In this construct a destabilization domain (DD) is fused to a red fluorescence protein (RFP) under the control of YAP responsive promoter (8×GTIIC). In the absence of trimethoprim (TMP), RFP is rapidly degraded. Adding TMP leads to accumulation of RFP only when YAP responsive promoter is activated^49^. Infected cells were then selected using 1 mg/ml of G418.

The bEnd.3 cells (ATCC CRL-2299) were cultured in complete DMEM/F12- with GlutaMAX supplemented with 10% fetal bovine serum (Eurobio, CVFSVF00-01), penicillin-streptomycin (100U/ml) (Invitrogen 15140122). The conditioned media from bEnd.3 cells was collected as follows: once cells reached 90-95% of confluence (in their growth culture media), they were washed using NeuroCult basal medium. Next, NeuroCult basal medium, NeuroCult proliferation, 20 ng/ml EGF, 20 ng/ml, bFGF 5 μg/ml, heparin and gentamycin were added. After 24 h the supernatant was recovered, centrifuged, and filtrated before being frozen. In parallel, the same volume of medium was placed in 10 cm^2^-plate with no cell to be used as a control.

### siRNA and inhibitors

For siYAP experiments, MGG4-Nestin cells were seeded into 24 well plate before transfection. Cells were transfected with 30pmol of siRNA-YAP1 (#4392420; Ambion by Life Technologies) or siRNA-Control (#4390843; Ambion by Life Technologies) using lipofectamine RNAiMAx Reagent (#13778; life technologies) for 48 h at 37 °C. RNA extraction and qPCR have been done to check the efficiency of YAP silencing using YAP1 TaqMan assay (4331182, Thermo-Fisher Scientific).

### In vitro γ-irradiation and TMZ treatment

Neurospheres were centrifuged, either resuspended with accutase as single cells or kept as small neurospheres and irradiated using a Cs-137 source (GSR Cs137/C, Gamma Service Medical GmbH) at the indicated doses. For the TMZ treatment, neurospheres were centrifuged, resuspended with accutase as single cells, and seeded at optimal density depending on the experiment. TMZ was diluted in DMSO and used at indicated concentrations.

For combinatorial experiments (γ-irradiation and TMZ) cells were first irradiated as indicated above before seeded in the presence of TMZ at indicated concentrations.

### NSC12 treatment

NSC12 (a FGF2/FGFR2 interaction inhibitor) was purchased from Selleck Chemicals (#S7940, Selleck, Texas, USA) and used at indicated concentrations for 3 days before analysis.

### RT-PCR

Total RNA was extracted, DNAse-digested to remove DNA contamination and purified using the QIAGEN RNeasy Mini Kit (QIAGEN, #74104) according to the manufacturer’s instructions. RNA quality and concentration were determined using a Nanodrop (Thermo Fisher). 1ug of RNA was then reverse-transcribed using superscript III (Thermo Fisher, #18080044). qPCR was then performed using the Fast advanced master mix in the light cycler QuantStudio5 (Applied Biosystems). Inventoried Taqman assays were first validated for maximal efficiency.

### Reprogramming using Incucyte

#### Effect of conditioned media from blood vessels on reprogramming

4.000 MGG4 Nestin^P^-dTomato cells were seeded on a low attachment 96-well-plate in conditioned media from blood vessels (0,5 brain/ml of CM-Bv vs CM-Control) or conditioned media from bEnd.3 cells (50% CM-bEnd vs CM-Control). The real-time visualization of Nestin^P^ Tomato reporter was performed using an IncuCyte live-cell imaging system (Sartorius). Images were taken every 3 h at ×4 magnification using RFP channel and phase contrast for 4 days. The IncuCyte Basic Software was used to perform image analysis. Cell segmentation was performed by using the phase contrast images. An area filter was applied to exclude objects below 50 μm^2^ (debris). Red channel background noise was subtracted with the Top-Hat method of background non-uniformity correction with a radius of 100 μm and a threshold of 0,6 red corrected units. Fluorescence signal was quantified as follows: the area of cells expressing tdTomato divided by the total area of cells.

#### Reprogramming of the Nes-LO cells upon treatment

MGG4-Nestin^P^-dTomato cells irradiated (2 or 5 Gy) or not were seeded on a collagen-I coated (50 μg/ml) 96 well plate (3000 cells per well) in Neurocult complete media or Neurocult complete media containing 0, 25, 50 or 100 μM of TMZ. The real-time visualization of Nestin^P^-dTomato reporter was performed using an IncuCyte live-cell imaging system. Images were taken every 3 h at ×20 magnification using RFP channel and phase contrast for 5 days. To analyze the reprogramming, raw images were extracted, and a custom ImageJ macro was designed to quantify the number of GFP+dTomato+ cells and GFP+dTomato- cells at each time point for all timepoints. The percentage of double positive cells was then calculated.

### FACS sorting

Neurospheres were collected, centrifuged, and resuspended as single cell suspension with accutase. Cells were then washed with PBS, centrifuged, and resuspended in neurocult media before filtering in a FACS tube. Cells then loaded into the BD FACSAria^TM^III sorter (BD Biosciences). Viable cells were first gated based on their size and granularity on FSC-A/SSC-A parameters. Doublets were excluded using both FSC-A/FSC-H and SSC-A/SSC-H parameters. Finally, cells were plotted for their FITC and dTomato parameters. For optimal sorting, GFP-neg cells were excluded and sorting was performed on GFP-pos/dTomato-neg cells for NesLO cells and GFP-pos/dTomato-pos cells for NesHI cells. To clearly separate the two populations, gates were placed at each extremity of the dTomato intensity plot.

### FACS analysis

#### Reprogramming and cell death analysis

Nestin^P^-dTomato MGG4, MGG18 or GL261 cells were seeded at the optimal density, treated (IR or TMZ) or not and cultured for several days according to the experiment. On the day of FACS analysis, neurospheres of each condition were collected, centrifuged, and resuspended with accutase as single cell suspension. Cells were then washed with PBS, centrifuged and resuspended in MACS buffer and divided in 2 FACS tubes: one for the dTomato analysis, the other one for cell death analysis. For reprogramming analysis, cells were loaded into the BD LSRFortessaTM Cell Analyzer (BD Bioscience). Live cells were first gated and doublets were then excluded as explained above. Nestin^P^-dTomato cells were detected using the PE channel. MGG4, MGG18 or GL261 naïve cells were used as control. For cell death analysis, Sytox (0.5 μM; Thermo Fisher, #S34857) was added in the flow cytometry tube containing cells and incubated for few minutes. Cells were then loaded into the FACS analyzer. Debris were discarded based on FSC-A/SSC-A parameters and Sytox positive cells were then detected in the BV421 channel. Cells without Sytox of each and all conditions were used as control for proper identification of Sytox positive cells. Analysis of all the recorded FACS data were then performed using FlowJo v10.7.2 software (BD Biosciences).

#### YAP/TAZ activity reporter analysis

For YAP/TAZ activity experiments, MGG4 cells expressing the YAP/TAZ activity reporter were plated at the optimal density on collagen I (Thermo Fisher, #A1048301) coating in 50% bEndCM or 50% CTL media, with or without trimethoprim (TMP, Sigma, #T7883) (10 μM final). At day 3, cells of each condition were collected, centrifuged, and resuspended with accutase as single cell suspension. Cells were then washed with PBS, centrifuged, and resuspended in MACS buffer. Cells were loaded into the FACS analyzer (BD Fortessa). Live cells were first gated, and doublets were then excluded as explained above. DsRed positive cells were detected using the PE channel. Cells of each condition, incubated without the TMP were used as control for gating. Analysis of all the recorded FACS data were then performed using FlowJo.

#### Slow cycling cells assay

Slow cycling versus highly proliferative cells were quantified in MGG4 Nestin^P^ dTomato cells using the CellTrace^TM^ Cell Proliferation Kit (Thermo Fisher Scientific, #C34572). In this assay, Celltrace^TM^ agent binds covalently to intracellular amines, resulting in retained fluorescent staining. The dye is then progressively diluted when cells divide. On day 0, neurospheres were collected, centrifuged, and resuspended with accutase as single cell suspension. Cells were then washed with PBS, centrifuged, and incubated for 20 min at 37 °C with the CellTrace^TM^ Far red dye or not. Cells were then washed with media containing 1% FBS to quench the fluorescence, centrifuged, washed with PBS and resuspended in NC media before seeding. At the desire timepoint cells of each condition were collected, centrifuged, and resuspended with Accutase as single cell suspension. Cells were then washed with PBS, centrifuged, and resuspended in MACS buffer and loaded into the FACS analyser. Live cells were first gated, and doublets were then excluded as explained previously. Celltrace dye intensity was detected using the 647 channel. Analysis was then performed using FlowJo. Mean fluorescent intensity was analyzed in each subpopulation of cells (NesLO and NesHI) after normalization on day 0. Mean fluorescent intensity (MFI) was analyzed in each subpopulation of cells (NesLO and NesHI) after normalization on day 0. The time to undergo cell division was calculated by the following formula: 2X = B with X as the number of cell division and B as the ratio (initial MFI)/(final MFI). Therefore, X= loGBM/log2, based on the fact that at every cell division the fluorescence intensity is divided by two^84^.

#### Cell cycle

Click-iT EdU Alexa Fluor 647 kit was used (#C10424; Thermo Fisher Scientific). Briefly, MGG4 Nestin^P^ dTomato cells were seeded at an initial density of 1 × 10^5^ cells/well in 6-well plates. Depending on time points and treatments employed (5 Gy irradiation or preconditioning with bEnd.3 CM), cells were harvested, resuspended as single cell using accutase, washed with PBS and incubated with EdU at a concentration of 10 μM for 2 h at 37 °C. EdU incorporation was subsequently detected by Alexa Fluor 647 azide, followed by Sytox staining as per manufacturer’s protocol (Thermo Fisher Scientific). Using FACS Fortessa and FlowJo 10, the fraction of cells in SubG1, G0/G1, S and G2/M phases were determined in each condition. The histogram plots for fraction of cells in each cell cycle phase was plotted using GraphPad 8 software.

FACS gating strategies provided in Supplementary Fig. 17,18.

### γ-H2AX assay

#### γ-H2AX immunolabelling

MGG4 Nestin^P^-dTomato cells, MGG4-CMV^P^GFP cells and MGG4 cells were seeded at an initial density of 2 × 10^6^ cells/ml in T75 flasks. After 48 h, the neurospheres were dissociated with accutase and washed with 1XPBS. After fixation and permeabilization cells were incubated with primary antibody for anti-γH2AX (ps139, clone N1_431, BD biosciences 1:100) for 1 h at room temperature. Following which the cells were stained with Alexa fluor 647 conjugated secondary Ab (1:500) for 30 min. Lastly, the cells were suspended in MACS buffer and stained with DAPI 1 μg/ml^85^.

#### Data acquisition using ImageStream and IDEAS

All samples were run through the ImageStream X MKII (ISX MKII) imaging flow cytometer (LUMINEX Corporation, Austin, Texas) and data was acquired with ISX INSPIRE software. Images of cells were acquired for each sample at 60X magnification with the extended depth field (EDF) mode by using 4 lasers set as 405 nm 20 mW for DAPI, 488 nm 100 mW for GFP, 561 nm 170 mW for Nestin and 642 nm 150 mW for γ-H2AX; brightfield images were captured in channel Ch01 and Ch09, while the DAPI images were captured in channel Ch07; GFP on channel Ch02, Nestin (dTomato) on channel Ch03 and γ-H2AX on channel Ch11. Data were recorded by gating on Ch01 area feature and Ch01 Aspect Ratio feature, allowing to select cells by size and avoiding debris. For compensation purposes, MGG4 cells were single stained with DAPI or γ-H2AX. The data analysis was done by using the Image Data and Exploration Analysis Software (IDEAS) package (v6.2). Compensation matrix and scatted profile for each channel were applied and following gating strategy applied: we selected cells in focus with Ch01 Gradient RMS feature (Focus), and isolated single cells using Ch01 area and Aspect Ratio Intensity features (Singlets). We then gated on DAPI positives nuclei (DAPI + ), followed by the isolation of gH2AX positives events γ-H2AX +  and finally the identification of NesHI and NesLO populations. For γ-H2AX analysis in both respective NesHI and NesLO population, we first quantified geometric mean fluorescence intensity of γ-H2AX in each population. And then, to quantify the nuclear γ-H2AX foci formation in each population per cell, we created a specific mask allowing the identification of all γ-H2AX peaks of intensity and exclude background noise (Peak (M11, Ch11, 4)), and apply it to a spot count feature. The plots and curves for spot quantification and geometric mean fluorescence intensity of γ-H2AX in each condition was plotted using GraphPad 8 software.

### Chemotaxis assay

For chemotaxis experiments u-slide chemotaxis slides (Cat number-80326 Ibidi GmbH, Germany) were used. These are microscopy slides equipped with 3 chambers each consisting of one channel for cells and 2 reservoirs for media with and without chemotaxis factors. Following the manufacturer’ guidelines, channels were coated with 50ug/ml of collagen 1 for 1 h at 37 °C. After quick washes with 1XPBS, the cells at a concentration 10^7^ cells/ml were seeded in the channels. Once the cells were well attached in the channels, bEnd.3 CM was added at a concentration of 50% (diluted with Neurocult complete media) in the first reservoir while in the second reservoir we added the control media. In other two chambers of the slide, a positive and a negative control with bEnd.3 CM or control media in both reservoirs were loaded. This allowed us to analyse specifically chemotaxis and avoiding any potential chemokinesis effect induced by CM*.*

After preparing the slides, the migration was recorded with a DMi6000B inverted widefield Microscope (Leica). The slides were maintained at 37 °C in a humidified atmosphere of 5% CO2. A series of time-lapse videos were captured at magnification of ×10 for each chamber at an interval of 10 min for a duration of 24 h in the following channels: brightfield, GFP 500-550 nm and DsRed (590–650 nm). For cell tracking, a custom-made manual tracking (Fabrice Cordelières; Institut Curie, Orsay, France) plugin by ImageJ (National Institute of Health, Bethesda, USA) was used. Around 40–60 cells were tracked per condition in each experiment. After tracking, the paths of individual cells were visualized by plotting the trajectory plots of each condition by importing the x and y coordinated of cells in the Chemotaxis and Migration tool developed by Ibidi. Using this tool, we further analyzed different chemotaxis parameters, which evaluate the directed migration of cells in response to bEnd.3 CM: forward migration indices in parallel or perpendicular to the direction of chemotaxis gradient, accumulated distance, Euclidean distance, velocity and directness^86^.

### Immunofluorescence of YAP

Cells were seeded on collagen-I (MGG4) or fibronectin (GL261) in an Ibidi chamber slide (m-slide 8 well Ibidi GmbH, Germany, #80826) for 3 h, thus allowing them to nicely adhere. Cells were then fixed with 4% PFA, permeabilized using PBS Triton X-100 0.1% and then blocked in 10% FBS, PBS Triton X-100, 0.1% for 1 h. Cells were then incubated overnight with primary antibody against YAP (D8H1X, XP® Rabbit mAb #14074 S) in blocking buffer at 4 °C. Cells were then washed in PBS and incubated with Alexa Fluor-conjugated secondary antibodies (1:500; Thermo Fisher Scientific) for 1 h at RT and finally stained with DAPI 1 μg/ml. Images were acquired using an inverted microscope (Leica DMI6000B), 40X magnification. Images were taken for brightfield, DAPI and YAP (far red). Nuclear versus cytoplasmic YAP signal was analyzed using a custom ImageJ macro designed to quantify the signal in each compartment.

### Animals

All animal procedures were conducted in compliance with recommendations of the European Community (2010/63/UE). 7 to 8-week-old female Swiss nude mice (Crl:NU(Ico)-*Foxn1*^*nu*^; Charles River) were used in our studies. These mice were housed in temperature and light controlled facility with maximum five mice per cage. Mice were routinely observed and weighted to ensure that interventions were well tolerated. Animal experimental procedures were specifically approved by the ethics committee of Institut Curie (CEEA-IC #118; 2018-010).

### Tumor growth monitoring and treatment protocols

For the orthotopic implantation of the patient-derived MGG4 cell line, mice were anesthetized with isoflurane and secured in stereotactic head holder. 20.000 MGG4-Gluc cells in 5 μl of media were implanted at the following coordinates according to Bregma: x = 2; y = −0.5; z = −3/−2 mm.

In vivo tumor growth was monitored using an established Gluc assay^83^. In short, 3–4 weeks post tumor implantation, Gluc activity was routinely recorded in the blood. Blood Gluc activity was measured using Coelentrazine (100 μM; Nanolight Technologies) by a Tristar2 multi-modal microplate reader (Berthhold technologies) in the luminescence mode. Before treatment, the blood Gluc activity was required to reach a predefined threshold range of 2–5 × 10^6^ RLU/s. MGG4-Gluc tumors reached the predefined Gluc threshold at median period of 90 days. For in vivo irradiation treatment the Small Animal Radiation Research Platform (SARRP, XStrahl), an image-guided micro X-ray irradiator was employed; wherein the treatment group (10–15 mice) was treated with a dose 10 Gy. In the TMZ in vivo study, 10 mg/kg of TMZ (Sigma T2577) prepared in DMSO solution in treatment group was intraperitoneally injected while control group was injected with the same amount of DMSO. Post-irradiation or TMZ injection, the tumor growth was monitored by measuring Gluc activity in blood over a period of 7 days. At day 7, mice were euthanized. For robust comparison, we ensured that we had time-matched Gluc values for control and 10 Gy mice.

In this study, the determination of maximal tumor size/burden was based on a combined assessment involving GLuc measurement and clinical behavior evaluation. The experimental protocol established a maximum threshold for tumor burden when GLuc reached more than 15 million GLuc bioiluminiscence units. Otherwise, the endpoints in this study were determined by a loss of 15-20% of the mouse initial body weight and/or the degradation of the general condition of the animal such as prostration, loss of coordination, loss of alertness or cutaneous lesions. In adherence to the established criteria, the maximal tumor size/burden was carefully monitored and did not exceed the predetermined threshold, thus maintaining the ethical standards outlined by the research institution and its regulatory bodies.

### Tissue harvesting and IHC

Mouse brains were heart-perfused with 4%PFA under controlled pressure. The harvested brains were immersed in 4%PFA for 24–48 h. After PBS washes and dehydration step with increasing gradient of ethanol percentage solutions, the brains were embedded in paraffin. All paraffin-embedded blocks were sectioned to obtain coronal sections of 7 μm. To visualize general tumor histopathology features, tissue sections were routinely H&E stained. For other IHC staining, sections were deparaffinized and then underwent heat induced antigen retrieval step in citrate buffer pH6 for 20 mins. The sections were then subjected to blocking and incubation overnight at 4 °C with the following primary Abs: anti-Nestin (10C2; 1:200; Ebiosciences or #PAS-82905, 1:1000, ThermoScientific), anti-hMito (113-1; 1:200 or 1:50; Millipore), anti-CD31 PECAM (#AF-3618, 1:100, R&D Systems) and anti-CD34 (EPS73Y; 1:500; Abcam). After incubation with primary Abs, the sections were incubated with HRP or AP secondary Ab polymer for 25 mins at RT. For Biotin-HRP detection system, the revelation was done using for Vector Substrate Peroxydase DAB kit and Substrate ImmPACT Vector Red AP kit. In both cases the sections were counter stained with hematoxylin for nuclear staining and finally the sections were mounted using aqueous based mounting media. For immunofluorescence, secondary antibodies (Alexa Fluor Donkey anti-Rabbit 488) (#A21206, 1:500, ThermoScientific), Dylight Donkey anti-Mouse 650 (#DkxMu-003-D650NHSX, 1:500, Diagomics), Alexa Fluor Donkey anti-Goat 555 (#A21432, 1:500, ThermoScientific) were applied for 30 min at room temperature. Finally, the slices were mounted using Fluoroshield with DAPI Histology Mounting Medium (#F6057, Merck).

### Tissue clearing and whole mount immunofluorescence

MACs clearing was performed according to the manufacturer’s instruction (#130-126-719; Miltenyi Biotech). Briefly, after perfusion and fixation with PFA 4%, adult mouse brains were collected, washed with PBS and embedded in 4% agarose for cutting into 500um slices with vibratome. Brain slices were permeabilized overnight at RT and incubated with primary antibodies (chicken GFP antibody from AvèsLabs #GFP-1020; rabbit mCherry antibody from Curie Facility #APR-13; Lycopersicon Esculentum Lectin dylight-649 #DL-1178 Vector Laboratories) for3 days at 37 °C. After washes with staining solution 5 times, the secondary antibodies were added (AlexaFluor555 goat anti-rabbit #A21429 Invitrogen or goat anti-chicken-555 #ab150170 Abcam) overnight at 37 °C. After washes with staining solution 3 times, labeled slices were embedded in 1,5% agarose and dehydrated with a series of ethanol dilutions (50% ethanol / 70% ethanol / 100% ethanol containing 2%Tween 20). After deshydration, the embedded slices were transferred on tubes containing clearing solution for 6 h. 12 bits images were acquired with a Leica SP8X inverted confocal laser scanning microscope (CLSM), equipped with a 16x FLUOTAR immersion objective (NA = 0.6). The objective ring was adjusted for oil immersion (RI 1.51). Sequential excitation mode (647 nm and 555 nm obtained with a white light laser (WLL)) was used to collect images on GaAsP Hybrid photon detectors. Emission was detected at 660-710 nm upon excitation at 647 nm and at 575‐625 nm upon excitation at 555 nm. The whole system was driven by LAS X software (Leica).

### Image analysis

Whole slide scan images of sections at 20X magnification were obtained using ZEISS AXIO Imager Z2 microscope. Automated quantification of staining was performed using Visiopharm (VIS; Visiopharm A/S, Hoersholm, Denmark). Blinding during analysis was used for all the in vivo experiments.

#### Nestin+ nuclei quantification

An algorithm-based analysis protocol package (APP) was developed to detect the tumor area in each tissue section. A deep-learning-based APP was created to distinguish tumor vs normal tissue based on the differences in cell density.

For Nestin positive nuclei detection in the defined tumor ROI, a threshold-based APP was designed. In this APP the differences in Nestin intensity and feature like Fast Red were used to detect and classify nuclei as NesHI or NesLO (or negative). Moreover, additional steps in the APP allowed us to separate and detect highly dense nuclear regions (NesHI nuclei, NesLO,). To calculate the percentage Nestin positive nuclei in tumor region:

$$\%{Nestin}\,{positive}\,{nuclei}=\frac{{Number}\,{of}\,{Nestin}\,{positive}\,{nuclei}}{{Total}\,{number}\,{of}\,{nuclei}\,{in}\,{tumor}\,{ROI}}\times 100$$

The tumor area was also calculated for sections in treatment and control group to ensure that there were no significant differences between the two groups.

#### CD34-hMito Vessel co-option quantification

For detection of tissue, an APP was developed, which differentiated between tissue and background based on a classification method. Next, for tumor detection, a similar APP (like one used for Nestin+ nuclei quantification) was created to distinguish tumor vs normal tissue based on the differences in cell density, in addition tumor border was defined based on decreasing cell density from tumor core.

Based on CD34 (in brown) for blood vessels and hMito (in red) for tumor cells staining, an APP was designed to detect blood vessels and tumor cells. In next steps, we specifically detected tumor cells in the border of tumor in contact with blood vessels (vessel co-opting cells) and also quantified tumor cells present in total.

### Tissue collection and RNA extraction

Mice were sacrificed using the standard method of cervical dislocation, following which the brain was immediately harvested. Dissect pieces of tumor were immediately immersed in RNAlater solution (Invitrogen). After 2–3 h of incubation at RT, the tumor pieces in RNAlater were stored at 4 °C for 1 week before initiating RNA extraction. Tumor tissues were homogenized using Precellys CK28 Hard tissue homogenizing columns with ceramic beads and evolution homogenizer (Bertin Corp). Following the homogenization, the RNA was extracted using RNeasy Mini kit (Qiagen). For the RNA-seq, only the probes aligned to human genes were taken in account.

### Intravital microscopy

EGFP-Nestin^P^-dTomato MGG4 tumor cells were stereotactically implanted into the right brain cortex of 8- to 10-week-old female immunocompromised Swiss nude mice (Crl:NU(Ico)-*Foxn1*^*nu*^; Charles River). Injections were stereotactically performed at 0.5 mm right from sagittal sinus, 1 mm caudal to the bregma and at a depth of 0.5 mm from the brain surface. One week prior to intravital microscopy, 7-mm-diameter cranial windows were surgically implanted to dynamically follow tumor cells intravitally. High speed driller with 0.6 mm burr-tip diameter was used to perform the craniotomy. Transparent cover glass was gently placed and glued to the skull with cyanocrylate and acrylic powder.

Intravital multiphoton microscopy was performed by using an A1R MP+ multiphoton microscope (Nikon Instruments Inc., USA). Mice were anaesthetized with isoflurane and cranial windows were fixed to a specific platform to avoid undesired movements during imaging. Retro-orbital injection of 0.1 ml of Cascade Blue-tagged dextran was done to visualize blood vessels^87,88^. Sequential imaging was performed with the use of 920 nm (for EGFP) and 990 nm (for Nestin^P^-dTomato) excitation laser lights. The used emission filters were: 400-492 nm (for CascadeBlue), 500-550 nm for EGFP and 563-588 nm for dTomato. Resulted 2D images and 3D z-stacks were processed with Imaris Image Analysis software.

### Ex vivo mouse brain vessels

The isolation of mouse brain vessels was based on a published protocol^89^ with some modifications. Brains from C57 BL/6 mice (Charles River Laboratories) were washed in PBS and transferred into 1X HBSS (Sigma, #H4641) with 10 ml of Hepes (Sigma, H3375). All following steps of brain vessel isolation were carried out at 4 °C. Brain tissues were cut into pieces of approximately 1 mm using scalpel and homogenized using a 5-cm^3^ Potter-Elvehjem tissue grinder (Wheaton, 357979, mortar size 10 ml) with 20 strokes of the pestle. The resulting homogenate was centrifuged at 2000g for 10 min. After removing the supernatant, the pellet containing the whole cortex homogenate was resuspended in 18% Dextran (Sigma, 31390) solution in HBSS buffer with 10 mM Hepes by vigorously shaking. Centrifugation at 3220 g for 30 min resulted in a pellet containing the brain vessels and a white myelin-rich layer of floating glial and neuronal cells at the top, which was removed together with the supernatant. The blood vessel pellet was resuspended in HBSS buffer with 10 mM Hepes and 1% BSA (Sigma, #A2153) and passed through a 20 m cell strainer to remove single cells and debris. The brain vessels on top of the filter were washed and then collected by inverting and rinsing the cell strainer with HBSS with 10 mM Hepes and 1%BSA. After filtration, the blood vessels were pelleted by centrifugation at 2000 × *g* for 5 min and resuspended in Neurocult complete medium to keep a ratio of 0.25 brain for 1 ml of media (24-well plate). The conditioned medium from blood vessels was collected 24 h after the seeding of blood vessels according to the ratio of 0.25 brain per 1 ml of media. The supernatant was recovered, centrifuged, and filtrated before being frozen. In parallel, the same volume of medium was placed in 24-well plate without blood vessels to be used as a control.

Immunofluorescence staining of blood vessels was performed after fixation with 4%PFA in PBS for 20 min at RT, washed twice, permeabilized and blocked overnight with 0,25%Triton-X 100, 2% FBS in PBS. Primary antibodies were diluted in the permeabilization/blocking solution and incubated at 4 °C. The blood vessels were washed 3X with 1%BSA. Alexa Fluor-conjugated secondary antibodies (1:500, Thermo Fisher Scientific) were incubated for 4 h at RT. Blood vessels were washed twice, then resuspended in approximately 30ul 1%BSA and subsequently mounted using mounting medium with DAPI.

### Vascular association to blood vessels

Blood vessels were seeded (0.25 brain/ml; in 24-well plate) on collagen-I coating (50 μg/ml; Corning #354236) to allow their attachment (24h-37 °C, 5% CO2). The MGG4 Nestin^P^-dTomato cells were added on top of the brain vessels (20.000 cells for 0,25 brain/ml of culture). The vascular association was analyzed using an inverted microscope (Leica DMI6000B) at 37 °C with 5%CO2 for 24 h. Images were taken with GFP and dTomato and bright-field filters (10X magnification, every 10 min for 24 h for multiple positions). Further analyses were performed using ImageJ software. Quantification of vascular association was calculated according to the following formula: (number of Nestin positive cells attached to vessel)/ (total number of Nestin positive cells in the field) compared to (number of Nestin negative cells attached to vessel)/(total number of Nestin negative cells in the field). In parallel, the quantification of the reprogramming was performed by calculating the number of Nestin positive cells normalized by the total number of cells in presence or not of vessels.

### Brain slice co-culture

The organotypic brain slice culture was based on previously published protocol^90^. Briefly, brain slices were cut using a vibratome (thickness at 250um in dissection buffer containing G-Glucose 1 M; NaHCO_3_ 1 M; MgCl_2_ 6H_2_O 100 mM; CaCl_2_ 2 H_2_O 100 mM; Hepes 0.25 M pH7,4; amphotericin B 1X; penicillin-streptomycin in HBSS 1X) after embedding brain in 4% of low-melting agarose. Then brain slices were transferred on the top of a free-floating nucleopore membrane (13-mm-diameter, 0,8-um pore size, polycarbonate, WHA110409-Sigma Aldrich) previously placed in a 24 well-plate containing NeuroCult NS-A Medium with the proliferation supplement (StemCell Technologies); 10mM G-Glucose; amphotericin B 1X; penicillin-streptomycin 1X; Glutamax 1X (Invitrogen) and kept at 37 °C for maximum one week. Immunostaining of brain slices was performed to attest the survival of all cell components of the brain microenvironment.

For live imaging, brain slices were maintained overnight at 37 °C in the media. On the following day, brain slices were labeled with lectin-647 (1 h at RT; 4 ug/ml; Vector #DL1178), and then maintained in the bottom of a u-slide 8 well glass bottom (IBIDI chamber) with 2% agarose covered with Neurocult basal medium. For MGG4 Nestin^P^-dTomato cell transplants, a standard Gilson pipet was used to deposit 20.000 cells in the smallest volume possible. An inverted Nikon Spinning-disk microscope with 10X magnification was used for image acquisition at 37 °C and 5%CO_2_. Multistage positions and z-series corresponding to a range of 450 um were acquired every 20 min using 491, 561 and 642 nm wavelength. Imaris (Bitplane) was used to quantify the distance of MGG4-Nestin cells to vessels after creating segmentation for vessels and defining cells as spots at different time points.

### Spatial transcriptomics of GBM patients

Only samples containing the tumor core and the infiltrated cortex was used for this analysis. To quantify the overlap of the signatures of interest, we carried out spatially weighed correlation analysis for each individual sample^41^. The correlation coefficient in spatially resolved data needs to be addressed differently compared to data where every datapoint can be assumed to be independent. In the context of spatial weighted correlation measurements, the model needs to be corrected for effects of local neighbor dependencies. In our analysis, we only made use of samples that were clearly distinguishable by histological features (*n* = 3). Each sample was segmented into tumor core and the infiltrative area, which were then used to generated spatially weighted correlation arrays (c,c,n) (c = signatures and n = number of samples), which was reduced by mean to a *c x c* correlation matrix.

### RNA sequencing

Total RNA was isolated (RNeasy kit, Qiagen, #74104) from all conditions and cell lines described. RNA quality was assessed using Agilent 2100 Bioanalyzer (RNA 6000 Nano kit -Agilent Technologies). For the blood vessel co-culture, the co-culture was centrifuged, and filtered to focus on the cancer cells only for RNA extraction, for which the quality was assessed using an RNA 6000 Pico kit on Agilent 2100 Bioanalyzer.

RNA-seq libraries were constructed using Illumina library structure (Illumina), according to the manufacturer’s instructions, and were sequenced as 100 bp paired-end runs on the Novaseq 6000 (Illumina), resulting in an average of 30 million reads per sample.

The reads obtained were processed using the Curie Institute Nextflow RNA-seq analysis pipeline v3.1.8 (in vivo MGG4, MGG18, GL261, MGG4 TMZ), v3.1.7 (BV co-culture), v3.1.5 (MGG4 Tom high). Briefly, reads quality was assessed using FastQC and mapped to the reference genome (hg19/GRCh37 or mm10/GRCm38 for GL261) using STAR software. Finally, raw reads count tables were generated using STAR. Software versions and full pipelines are available at https://github.com/bioinfo-pf-curie/RNA-seq/.

Normalization and differential expression analysis were performed using DESeq2 R package (v1.30.0). DESeq2’s median of ratios normalization was extracted and used as input for Gene Set Enrichment Analysis (GSEA). GSEA was performed using the Broad Institute software and its publicly available gene signatures database (HALLMARK, KEGG, Gene Ontology), along with specific signatures downloaded or custom-made. Over-representation analysis (ORA) was performed with the web-based tool^91^, lollipop graphs with ShinyGO v0.741^92^, UpSet plots with FLAME^93^ and Ingenuity Pathway Analysis (IPA) by using the platform from Qiagen.

RNA-seq data have been deposited in NCBI’s Gene Expression Omnibus (GEO; https://www.ncbi.nlm.nih.gov/geo/) and are accessible through GEO Series accession number GSE218860.

### Single cell RNA sequencing and analysis

#### Cell preparation

MGG4 naïve cells cultivated as small neurospheres were collected, centrifuged, and resuspended with accutase as single cell suspension. Cells were then washed with PBS, centrifuged, counted, and irradiated (5 Gy) or not (control cells). At day 3 and day 5, neurospheres of both conditions (control and 5 Gy) were collected, centrifuged and resuspended with accutase as single cell suspension. Cells were then washed with PBS, centrifuged and dead cells were removed using the dead cell removal kit (Miltenyl Biotec, #130-090-101) according to the manufacturer instructions. Briefly, cells were incubated with Dead cell removal microbeads for 15 min at room temperature to remove dead cells. After the incubation the cell suspension was diluted with 1X binding buffer and loaded onto the column. Live cells were collected into the effluent. Column was rinsed with 1X binding buffer. Live cell fraction was then centrifuged and resuspended in 1X PBS BSA 0.04%. Cells were counted and resuspended at 10^6^ cells/ml.

#### 10X genomics procedure

For single-cell library preparation on the 10x Genomics platform, we used: the Chromium Single Cell 3′ v3.1 Library and Gel Bead Kit v3.1 (PN-1000121), Chromium Next GEM Chip G Single Cell Kit (PN-1000127), according to the manufacturer’s instructions in the Chromium Single Cell 3′ Reagents Kits V3.1 User Guide. Approximatively 3000 cells were loaded on the chip. The 10X capture and library preparation protocol was used without modification. Samples were sequenced on the Illumina Novaseq 6000.

#### Data preprocessing

Sequencing output reads were converted to FASTQ files using bcl2fastq (v2.20) and aligned to the hg38 reference genome with Kallisto aligner (v0.46.2)^94^. The resulting bus files were corrected, sorted then raw count matrix was generated with bustools (v.0.40.0) programs^95^. The raw matrix was filtered with the function emptyDrops from the DropletUtils package, using the method ‘cellranger’^96^. Filtered raw counts data was imported into Seurat R package (v4.0.3)^97^ for further processing and analysis. Raw transcript counts of gene-cell matrices were filtered to remove cells with total UMI counts lower than 4000 and higher than 11000; and cells with more than 20% mitochondrial genes. The UMI counts matrices were then normalized with Satija’s lab SCTransform method. Cell cycle scores were calculated with Seurat and used to regress out the cell cycle signal during normalization. Finally, the different datasets were integrated with Seurat’s anchors method using 3000 features.

#### Dimension reduction

Linear dimension reduction (principal component analysis) was applied on the 3000 genes with the highest variance identified by SCTransform and the number of principal components used in downstream analyses, 30 was chosen considering Seurat’s PCHeatmap and Elbowplot. Seurat’s implementation of Uniform manifold approximation and projection (UMAP) was applied on the reduced data for visualization in two-dimensional space.

#### Cluster analysis

Clusters were identified with IKAP algorithm^27^. IKAP uses Seurat graph-based unsupervised clustering. It generates various candidate clustering by tuning Seurat’s algorithm parameters, then computes a gap statistics for each clustering. The clustering with the highest gap increase is then selected. CerebroApp’s getMarkerGenes, which internally calls Seurat’s FindAllMarkers, was used to identify cluster-specific markers. To select widely and significantly overexpressed genes, the minimal logFC was set to 0,5 and the minimum percentage of cells to 0.75. For each cluster, FindMarkers function was used to calculate DE genes between treated and untreated cells.

#### Molecular 4-states cell classification

Cells were classified according to Suva’s lab method and the gene signatures they generated^11^. For each set of genes (G_j_), a score was attributed to each cell. This score was calculated as the difference between the average relative expression of the genes in (G_j_) and the average relative expression in a control gene set, i.e. Score_(G,)_ = av(Er(G(_j,i_)) – av(Er(G_j control, i_))). The control gene set was defined as first binning all analyzed genes into 30 bins of aggregate expression levels and then, for each gene in the gene-set (G_j_), randomly selecting 100 genes from the same expression bin. The cell was then attributed the state with the highest score between APC-like, NPC-like, AC-like, MES-like. For 2D representation, the y coordinate was calculated by the formula y = max(SCopc,SCnpc) – max(SCac,Scmes). The sign of the y coordinate allowed to separate cells into OPC/NPC (y > 0) versus AC/MES (y < 0). The x coordinate was defined for OPC/NC cells as x = log2( | SCopc – SCnpc | +1) and for AC/MES cells as x = log2( | SCac – SCmes | +1).

#### RNA velocity

RNA velocity was analyzed using the aligner velocyto (v0.17.17) and the scvelo toolkit (v0,2,3)^98,99^. Count matrices of pre-mature (unspliced) and mature (spliced) RNAs were obtained with velocyto. Scvelo functions were used with defaults parameters to filter and normalize the data. Future cell state was computed using a likelihood-based dynamical model (funtion velocity, diff_kinetics=True). For results representation, Seurat’s Umap representations were imported and scvelo functions were used to project velocities into Umap’s low dimension space.

#### Data visualization

For most steps of the analysis, plots were generated either with Seurat’s visualization functions or with R package ggplot2 or CerebroApp visualization and export functions. For RNA velocity, plots were generated with scvelo’s visualization functions.

### Liquid chromatography tandem mass spectrometry (LC-MS/MS) analysis

#### Material preparation

Cells were lysed in a buffer containing 8 M urea, 50 mM ammonium bicarbonate (ABC). Lysates were sonicated to decrease viscosity and centrifuged at 20,000 x g for 10 min. The protein concentration was measured using the BCA assay (Sigma). Equal amounts of proteins were then prepared (400ug of each condition) and reduced by adding 5 mM dithiothreitol (Sigma #D0632) and incubated for 30 min at 55 °C. Samples were subsequently alkylated by incubation with iodoacetamide (Sigma #I1149) at a final concentration of 10 mM for 30 min in the dark. Samples were then diluted 10-fold with 50 mM ABC to obtain a final concentration of urea > 1 M before overnight digestion with Trypsin/LysC (Promega #V5072) at 37 °C. Digested samples were incubated with 1% trifluoroacetic acid (Sigma #299537) for 15 min on ice and then centrifuged at 3,000 x g for 10 min to remove precipitate. Peptides were desalted using a SEP-PAK C18 cartridge (Waters #WAT054955) and eluted with 0,1% trifluoroacetic acid, 40% acetonitrile buffer and 90% of the starting material was enriched using Titansphere Phos-TiO kit centrifuge columns (GL Sciences #5010-21312) as described by the manufacturer. After elution from the Spin tips, the phospho-peptides and the remaining 10% eluted peptides were vacuum concentrated to dryness and reconstituted in 0.1% formic acid prior to LC-MS/MS of phosphoproteome and proteome analyses.

#### LC-MS/MS analysis

Peptides for MGG4 proteome analyses were separated by LC using an RSLCnano system (Ultimate 3000, Thermo Fisher Scientific) coupled online to an Orbitrap Exploris 480 mass spectrometer (Thermo Fisher Scientific). Peptides were trapped on a C18 column (2 cm × 75 μm inner diameter; nanoViper Acclaim^TM^ PepMap^TM^ 100, Thermo Fisher Scientific) with buffer A (2/98 MeCN/H2O (vol/vol), 0.1% formic acid) at a flow rate of 3 µl / min over 4 min. Separation was performed using a 50 cm × 75 μm C18 column (Thermo Fisher Scientific #164540), regulated to a temperature of 50 °C with a linear gradient of 3% to 32% buffer B (100% MeCN, 0.1% formic acid) at a flow rate of 300 nl / min over 211 min. MS full scans were performed in the ultrahigh-field Orbitrap mass analyzer in the m/z range of 375–1500 with a resolution of 120,000 (at m/z 200), an automatic gain control (AGC) set at 300% and with a maximum injection time (IT) set on custom mode. The 30 most intense ions were isolated (isolation width of 1.6 m/z) and further fragmented via high-energy collision dissociation (HCD) activation and a resolution of 15,000, an AGC target value set to 100% and with a maximum IT on auto. We selected ions with charge state from 2+ to 6+ for screening. Normalized collision energy (NCE) was set at 30 and dynamic exclusion of 40 seconds.

For MGG4 phosphoproteome analyses LC-MS/MS was performed as previously (same LC and MS system, trap column, column and buffers). Peptides were trapped on a C18 column with buffer A at a flow rate of 3 µl/min over 4 min and separation was performed using a C18 column, regulated to a temperature of 40 °C with a linear gradient of 3% to 29% buffer B at a flow rate of 300 nl/min over 91 min. MS full scans were performed in the ultrahigh-field Orbitrap mass analyzer in the m/z range of 375–1500 (120,000 resolution; AGC 300%; IT 25 ms). The 20 most intense ions were isolated and further fragmented via HCD (15,000 resolution; AGC 100%; IT 60 ms; selected ions 2+ to 6 + ; NCE 30) and with dynamic exclusion of 40 seconds.

For GL261 and PN-MGG4 proteome analyses, LC was performed as previously with an RSLCnano system (same trap column, column and buffers) coupled online to an Orbitrap Eclipse Tribrid mass spectrometer (Thermo Fischer Scientific). Peptides were trapped on a C18 column at a flow rate of 3.0 µl / min in buffer A for 4 min and separation was performed using a C18 column regulated to a temperature of 50 °C with a linear gradient from 2% to 30% buffer B at a flow rate of 300 nl / min over 211 min. MS1 data were collected in the Orbitrap (120,000 resolution; IT 60 ms; AGC 4 × 10^5^). Charges states between 2 and 5 were required for MS2 analysis, and a 45 s dynamic exclusion window was used. MS2 scan were performed in the ion trap in rapid mode with HCD fragmentation (isolation window 1.2 Da; NCE 30%; IT 60 ms; AGC 10^4^).

For GL261 and PN-MGG4 phosphoproteome analyses, LC was performed as previously with an RSLCnano system (same trap column, column and buffers) coupled online to an Orbitrap Exploris 480 mass spectrometer. Peptides were trapped on a C18 column with buffer A at a flow rate of 2.5 µl/min over 4 min and separation was performed using a C18 column regulated to a temperature of 50 °C with a linear gradient of 2% to 30% buffer B at a flow rate of 300 nl/min over 91 min. MS full scans were performed in the ultrahigh-field Orbitrap mass analyzer (range 375–1500; resolution 120,000; AGC 300%; IT 60 ms). The 20 most intense ions were isolated and further fragmented via HCD (resolution 15,000; AGC 100%; IT 60 ms; selected ions 2+ to 6 + ; NCE 30) and a dynamic exclusion of 40 seconds.

#### Data analysis

For identification, the data were searched against the Homo Sapiens (UP000005640_9606) UniProt database for MGG4 and PN-MGG4 samples and against the Mus Musculus (UP000000589 database downloaded 03/2020) for GL261 samples using Sequest HT through Proteome Discoverer (PD version 2.4). Enzyme specificity was set to trypsin and a maximum of two missed cleavage sites were allowed. Oxidized methionine, N-terminal acetylation, methionine loss and methionine acetylation loss were set as variable modifications. Phospho serine, threonine and tyrosines were also set as variable modifications in phosphoproteome analyses. Maximum allowed mass deviation was set to 10 ppm for monoisotopic precursor ions and 0.02 Da for MS/MS peaks from the Orbitrap Exploris 480 instrument and 0.6 Da for MS/MS peaks from the Orbitrap Eclipse Tribrid instrument. The resulting files were further processed using myProMS^100^ https://github.com/bioinfo-pf-curie/myproms v.3.9.3. False-discovery rate (FDR) was calculated using Percolator^101^ and was set to 1% at the peptide level for the whole study. Label-free quantification was performed using peptide extracted ion chromatograms (XICs), computed with MassChroQ^102^ v.2.2.21. For protein quantification, XICs from proteotypic peptides shared between compared conditions (TopN matching for proteome setting and simple ratios for phosphoproteome) with missed cleavages were used. Median and scale normalization at peptide level was applied on the total signal to correct the XICs for each biological replicate (N = 5). The phosphosite localization accuracy was estimated by using the PtmRS node in PD, in PhosphoRS mode only. Phosphosites with a localization site probability greater than 75% were quantified at the peptide level. To estimate the significance of the change in protein abundance, a linear model (adjusted on peptides and biological replicates) was performed, and p-values were adjusted using the Benjamini–Hochberg FDR procedure.

Proteins with at least 3 total peptides in all replicates (*n* = 5) and an adjusted *p* value ≤ 0.05 were considered significantly enriched in sample comparisons. Unique proteins were considered with at least three total peptides in all replicates. Kinase Enrichment Analysis (KEA) was performed using the web-based platform KEA3^103^ with a selected list of proteins (more than 0.8 of log2 fold change and shared between MGG4 and GL261 datasets) or KSEAapp (https://github.com/casecpb/KSEAapp/)^104^. For KSEAapp, KEA was performed with a *p* value threshold at 0.01 and a minimum of 5 substrates per kinase.

For the proteomic enrichment analysis shown in Fig. 7L the *cell state markers’ score* was performed by using the mean of the log2(ratio) of all cell state markers’ genes present in the proteomics dataset. This was carried out with the intention of evaluating the difference in protein expression levels between the two conditions in the time.

The mass spectrometry proteomics data have been deposited to the ProteomeXchange Consortium (http://proteomecentral.proteomexchange.org) via the PRIDE partner repository^105^ with the dataset identifier PXD042606.

### SNAIL

The design of the SNAIL probes and their use to detect specific relative single cell mRNA amounts was based on a published protocol^106^. Each probe was designed with 21 nucleotides to hybridize the target RNA with Tm of 60 °C, a 5’phosphate sequence, 3’OH sequence, and a Cy5 fluorescence sequence for detection. Human *Nestin* probes:

ACATTATTCCTCATCTGCAAACCCATACCAAGGTAGTTTAGTAGCCTGAAAGATA

ACATTATCTCCTTTTCCAGAGCTGTCAACCAAGGTAGTTTAGTAGCCTGAAAGATA

ACATTATTCTCTTGTCCCGCAGACTTACCAAGGTAGTTTAGTAGCCTGAAAGATA

ACATTACATTTTCCACTCCAGCCATCACCAAGGTAGTTTAGTAGCCTGAAAGATA

MGG4-Nestin^P^ Tomato cells were fixed with 1.6% PFA in PBS for 10 min then transferred to pre-chilled (−20 °C) methanol and kept at −80 °C for at least 15 min (and up to 1 wk). SNAIL probes were dissolved at 100 μM in ultrapure RNase-free water and pooled at a final concentration of 100 nM per oligo (1 gene detected by 4 probe pairs). The probe mixture was heated at 90 °C for 2 to 5 min and then cooled-down at RT. The samples were taken from −80 °C and equilibrated to RT for 5 min, washed by PBSTR (0.1% Tween-20, 40 U/mL RNAsin·In in PBS) for 2–5 min and incubated in 1× hybridization buffer (2X SSC, 10% formamide, 1% Tween-20, 20 mM RVC, 0.1 mg/ml salmon sperm DNA and pooled SNAIL probes at 100 nM per oligo) in 40 °C humidified oven with gentle shaking overnight. The samples were then washed for 2 min, twice, with PBSTR, followed by one 20 min wash in 4X SSC dissolved in PBSTR at 40 °C. Finally, the sample was briefly rinsed with PBSTR once at RT. The samples were then incubated for two hours with T4 DNA ligation mixture (1:50 dilution of T4 DNA ligase supplemented with 1X BSA and 0.2 U/μl of RNAsin) at room temperature with gentle agitation. Then samples were washed twice with PBSTR, incubated with RCA mixture (200U/ml of Phi29 DNA polymerase, 250 μM dNTP, 1X BSA and 0.2 U/μl of RNAsin) at 30 °C for two hours under agitation. The samples were next washed twice in PBSTR and 2,5 μM of detection oligo for human Nestin and CD31 were added and incubated at 37 °C for 30 min with shaking. After two washes with PBST, a staining with DAPI was performed 7 min at RT, then samples are washes with PBS and imaged.

### Statistical analysis

Data are expressed as mean ± SEM. All statistical tests were two-sided, and results were considered statistically significant at p < 0.05. All results shown in the manuscript are the outcomes of at least three biological replicates (different batches of cells) and independent experiments. Statistical analyses for all other experiments were performed using paired or unpaired two-sided t-tests or, when more than two groups were assessed, by ANOVA followed by Dunnett’s multiple comparisons test. Statistical analyses were performed using Prism (GraphPad Software Inc.). Blinding during analysis was used for all the in vivo experiments.

### Reporting summary

Further information on research design is available in the Nature Portfolio Reporting Summary linked to this article.

### Supplementary information

Supplementary information

Reporting Summary

Supplementary Movie 1

Supplementary Movie 2

Supplementary Data File 1

Supplementary Data File 2

Supplementary Data File 3

Supplementary Data File 4

Supplementary Data File 5

Supplementary Data File 6

Peer Review File

Description of Additional Supplementary Files

### Source data

Source data

## Data Availability

RNA-seq data have been deposited in NCBI’s GEO and are publicly available through GEO Series accession number GSE218860. The mass spectrometry proteomics data have been deposited to the ProteomeXchange Consortium via the PRIDE partner repository with the dataset identifier PXD042606, which is publicly available. The remaining data are available within the Article, Supplementary Information or Source Data file. Source data are provided with this paper.

## Code availability

Software versions and full pipelines are available at https://github.com/bioinfo-pf-curie/RNA-seq/ for RNA-seq, https://github.com/bioinfo-pf-curie/myproms for proteomics and https://github.com/casecpb/KSEAapp/ for KSEA.

## References

[1] Ostrom QT, Cioffi G, Waite K, Kruchko C, Barnholtz-Sloan JS (2021). CBTRUS statistical report: Primary brain and other central nervous system tumors diagnosed in the United States in 2014–2018. Neuro Oncol..

[2] Louis DN (2021). The 2021 WHO classification of tumors of the central nervous system: A summary. Neuro Oncol..

[3] Stepanenko, A. A. & Chekhonin, V. P. Recent advances in oncolytic virotherapy and immunotherapy for glioblastoma: A glimmer of hope in the search for an effective therapy? *Cancers (Basel)* **10**10.3390/cancers10120492 (2018). 10.3390/cancers10120492 PMC6316815 30563098

[4] Bikfalvi A (2023). Challenges in glioblastoma research: focus on the tumor microenvironment. Trends Cancer.

[5] Stupp R (2005). Radiotherapy plus concomitant and adjuvant temozolomide for glioblastoma. N. Engl. J. Med.

[6] Yabo YA, Niclou SP, Golebiewska A (2022). Cancer cell heterogeneity and plasticity: A paradigm shift in glioblastoma. Neuro Oncol..

[7] Verhaak RG (2010). Integrated genomic analysis identifies clinically relevant subtypes of glioblastoma characterized by abnormalities in PDGFRA, IDH1, EGFR, and NF1. Cancer Cell.

[8] Wang Q (2017). Tumor evolution of glioma-intrinsic gene expression subtypes associates with immunological changes in the microenvironment. Cancer Cell.

[9] Lee JK (2017). Spatiotemporal genomic architecture informs precision oncology in glioblastoma. Nat. Genet.

[10] Sottoriva A (2013). Intratumor heterogeneity in human glioblastoma reflects cancer evolutionary dynamics. Proc. Natl. Acad. Sci. USA.

[11] Neftel C (2019). An integrative model of cellular states, plasticity, and genetics for glioblastoma. Cell.

[12] Guilhamon, P. et al. Single-cell chromatin accessibility profiling of glioblastoma identifies an invasive cancer stem cell population associated with lower survival. *Elife* **10**10.7554/eLife.64090 (2021). 10.7554/eLife.64090 PMC7847307 33427645

[13] Chaligne R (2021). Epigenetic encoding, heritability and plasticity of glioma transcriptional cell states. Nat. Genet.

[14] Suvà ML, Tirosh I (2020). The glioma stem cell model in the era of single-cell genomics. Cancer Cell.

[15] Uribe, D. et al. Adapt to persist: Glioblastoma microenvironment and epigenetic regulation on cell plasticity. *Biology (Basel)* **11**10.3390/biology11020313 (2022). 10.3390/biology11020313 PMC8869716 35205179

[16] Dirkse A (2019). Stem cell-associated heterogeneity in Glioblastoma results from intrinsic tumor plasticity shaped by the microenvironment. Nat. Commun..

[17] Johnson KC (2021). Single-cell multimodal glioma analyses identify epigenetic regulators of cellular plasticity and environmental stress response. Nat. Genet.

[18] Xie XP (2022). Quiescent human glioblastoma cancer stem cells drive tumor initiation, expansion, and recurrence following chemotherapy. Dev. Cell.

[19] Larsson I (2021). Modeling glioblastoma heterogeneity as a dynamic network of cell states. Mol. Syst. Biol..

[20] Griveau A (2018). A Glial Signature and Wnt7 Signaling Regulate Glioma-Vascular Interactions and Tumor Microenvironment. Cancer Cell.

[21] Seano G (2018). Targeting the perivascular niche in brain tumors. Curr. Opin. Oncol..

[22] Seano G, Jain RK (2020). Vessel co-option in glioblastoma: emerging insights and opportunities. Angiogenesis.

[23] Ballestin A, Armocida D, Ribecco V, Seano G (2024). Peritumoral brain zone in glioblastoma: Biological, clinical and mechanical features. Front Immunol..

[24] Calabrese C (2007). A perivascular niche for brain tumor stem cells. Cancer Cell.

[25] Galan-Moya EM (2011). Secreted factors from brain endothelial cells maintain glioblastoma stem-like cell expansion through the mTOR pathway. EMBO Rep..

[26] Wakimoto H (2012). Maintenance of primary tumor phenotype and genotype in glioblastoma stem cells. Neuro Oncol..

[27] Chen, Y. C. et al. IKAP-Identifying K mAjor cell Population groups in single-cell RNA-sequencing analysis. *Gigascience* **8**10.1093/gigascience/giz121 (2019). 10.1093/gigascience/giz121 PMC6771546 31574155

[28] Ruiz-Moreno, C. et al. Harmonized single-cell landscape, intercellular crosstalk and tumor architecture of glioblastoma. *BioRxiv*10.1101/2022.08.27.505439 (2022).

[29] Jin X, Jung JE, Beck S, Kim H (2013). Cell surface Nestin is a biomarker for glioma stem cells. Biochem. Biophys. Res. Commun..

[30] Lothian C, Lendahl U (1997). An evolutionarily conserved region in the second intron of the human nestin gene directs gene expression to CNS progenitor cells and to early neural crest cells. Eur. J. Neurosci..

[31] Ferri, A., Stagni, V. & Barilà, D. Targeting the DNA Damage Response to Overcome Cancer Drug Resistance in Glioblastoma. *Int. J. Mol. Sci*. **21**10.3390/ijms21144910 (2020). 10.3390/ijms21144910 PMC7402284 32664581

[32] Chen J (2012). A restricted cell population propagates glioblastoma growth after chemotherapy. Nature.

[33] Varn FS (2022). Glioma progression is shaped by genetic evolution and microenvironment interactions. Cell.

[34] Wang L (2022). A single-cell atlas of glioblastoma evolution under therapy reveals cell-intrinsic and cell-extrinsic therapeutic targets. Nat. Cancer.

[35] Gangoso E (2021). Glioblastomas acquire myeloid-affiliated transcriptional programs via epigenetic immunoediting to elicit immune evasion. Cell.

[36] Schmitt CA, Wang B, Demaria M (2022). Senescence and cancer - role and therapeutic opportunities. Nat. Rev. Clin. Oncol..

[37] Hanahan D (2022). Hallmarks of cancer: New dimensions. Cancer Discov..

[38] González-Gualda E, Baker AG, Fruk L, Muñoz-Espín D (2021). A guide to assessing cellular senescence in vitro and in vivo. FEBS J..

[39] Puchalski RB (2018). An anatomic transcriptional atlas of human glioblastoma. Science.

[40] Yang AC (2022). A human brain vascular atlas reveals diverse mediators of Alzheimer’s risk. Nature.

[41] Ravi VM (2022). Spatially resolved multi-omics deciphers bidirectional tumor-host interdependence in glioblastoma. Cancer Cell.

[42] Rafii S, Butler JM, Ding BS (2016). Angiocrine functions of organ-specific endothelial cells. Nature.

[43] Singhal M, Augustin HG (2020). Beyond Angiogenesis: Exploiting angiocrine factors to restrict tumor progression and metastasis. Cancer Res.

[44] Degorre, C. et al. Mechanistic insights of radiation-induced endothelial senescence impelling glioblastoma genomic instability at relapse. *bioRxiv*, 10.1101/2021.12.13.472364 (2021).

[45] Pagliari S (2021). YAP-TEAD1 control of cytoskeleton dynamics and intracellular tension guides human pluripotent stem cell mesoderm specification. Cell Death Differ..

[46] Basisty N (2020). A proteomic atlas of senescence-associated secretomes for aging biomarker development. PLoS Biol..

[47] Zhao B, Li L, Tumaneng K, Wang CY, Guan KL (2010). A coordinated phosphorylation by Lats and CK1 regulates YAP stability through SCF(beta-TRCP). Genes Dev..

[48] Kwon H, Kim J, Jho EH (2022). Role of the Hippo pathway and mechanisms for controlling cellular localization of YAP/TAZ. FEBS J..

[49] Abdel Hadi L (2018). A bidirectional crosstalk between glioblastoma and brain endothelial cells potentiates the angiogenic and proliferative signaling of sphingosine-1-phosphate in the glioblastoma microenvironment. Biochim. Biophys. Acta Mol. Cell Biol. Lipids.

[50] Barrette AM (2022). Anti-invasive efficacy and survival benefit of the YAP-TEAD inhibitor verteporfin in preclinical glioblastoma models. Neuro Oncol..

[51] Muthukrishnan SD (2022). P300 promotes tumor recurrence by regulating radiation-induced conversion of glioma stem cells to vascular-like cells. Nat. Commun..

[52] Aggarwal V, Montoya CA, Donnenberg VS, Sant S (2021). Interplay between tumor microenvironment and partial EMT as the driver of tumor progression. iScience.

[53] Lüönd F (2021). Distinct contributions of partial and full EMT to breast cancer malignancy. Dev. Cell.

[54] Schmitt MJ (2021). Phenotypic mapping of pathologic cross-talk between glioblastoma and innate immune cells by synthetic genetic tracing. Cancer Discov..

[55] Lathia JD, Mack SC, Mulkearns-Hubert EE, Valentim CL, Rich JN (2015). Cancer stem cells in glioblastoma. Genes Dev..

[56] Carruthers RD (2018). Replication stress drives constitutive activation of the DNA damage response and radioresistance in glioblastoma stem-like cells. Cancer Res.

[57] Milanovic M (2018). Senescence-associated reprogramming promotes cancer stemness. Nature.

[58] Chakradeo S, Elmore LW, Gewirtz DA (2016). Is senescence reversible?. Curr. Drug Targets.

[59] Dou Z, Berger SL (2018). Senescence elicits stemness: A surprising mechanism for cancer relapse. Cell Metab..

[60] Salam R (2023). Cellular senescence in malignant cells promotes tumor progression in mouse and patient Glioblastoma. Nat. Commun..

[61] Jochems F (2021). The Cancer SENESCopedia: A delineation of cancer cell senescence. Cell Rep..

[62] Mongiardi, M. P., Pellegrini, M., Pallini, R., Levi, A. & Falchetti, M. L. Cancer Response to Therapy-Induced Senescence: A Matter of Dose and Timing. *Cancers (Basel)* **13**10.3390/cancers13030484 (2021). 10.3390/cancers13030484 PMC7865402 33513872

[63] Putavet, D. A. & de Keizer, P. L. J. Residual Disease in Glioma Recurrence: A Dangerous Liaison with Senescence. *Cancers (Basel)* **13**10.3390/cancers13071560 (2021). 10.3390/cancers13071560 PMC8038015 33805316

[64] Jeon HY (2016). Irradiation induces glioblastoma cell senescence and senescence-associated secretory phenotype. Tumour Biol..

[65] Aasland D (2019). Temozolomide induces senescence and repression of DNA repair pathways in glioblastoma cells via activation of ATR-CHK1, p21, and NF-κB. Cancer Res.

[66] Migliozzi S (2023). Integrative multi-omics networks identify PKCdelta and DNA-PK as master kinases of glioblastoma subtypes and guide targeted cancer therapy. Nat. Cancer.

[67] Kim YH (2017). Senescent tumor cells lead the collective invasion in thyroid cancer. Nat. Commun..

[68] Wang B, Kohli J, Demaria M (2020). Senescent cells in cancer therapy: Friends or foes?. Trends Cancer.

[69] Hoek KS (2008). In vivo switching of human melanoma cells between proliferative and invasive states. Cancer Res.

[70] Rabé M (2020). Identification of a transient state during the acquisition of temozolomide resistance in glioblastoma. Cell Death Dis..

[71] Liau BB (2017). Adaptive chromatin remodeling drives glioblastoma stem cell plasticity and drug tolerance. Cell Stem Cell.

[72] Gu J (2022). Targeting radiation-tolerant persister cells as a strategy for inhibiting radioresistance and recurrence in glioblastoma. Neuro Oncol..

[73] Thomsen MS, Birkelund S, Burkhart A, Stensballe A, Moos T (2017). Synthesis and deposition of basement membrane proteins by primary brain capillary endothelial cells in a murine model of the blood-brain barrier. J. Neurochem.

[74] Gouazé-Andersson V (2016). FGFR1 induces glioblastoma radioresistance through the PLCγ/Hif1α Pathway. Cancer Res.

[75] Limbad C (2022). Senolysis induced by 25-hydroxycholesterol targets CRYAB in multiple cell types. iScience.

[76] Huang, C., Santofimia-Castaño, P. & Iovanna, J. NUPR1: A Critical Regulator of the Antioxidant System. *Cancers (Basel)* **13**10.3390/cancers13153670 (2021). 10.3390/cancers13153670 PMC8345110 34359572

[77] Zanconato F, Cordenonsi M, Piccolo S (2016). YAP/TAZ at the Roots of Cancer. Cancer Cell.

[78] Haley EM, Kim Y (2014). The role of basic fibroblast growth factor in glioblastoma multiforme and glioblastoma stem cells and in their in vitro culture. Cancer Lett..

[79] Gouaze-Andersson V (2016). FGFR1 induces glioblastoma radioresistance through the PLCgamma/Hif1alpha pathway. Cancer Res..

[80] Ader I (2002). The radioprotective effect of the 24 kDa FGF-2 isoform in HeLa cells is related to an increased expression and activity of the DNA dependent protein kinase (DNA-PK) catalytic subunit. Oncogene.

[81] Wańkowicz P, Rogińska D, Machaliński B, Nowacki P (2020). Expression of markers of neural stem and progenitor cells in glioblastoma multiforme in relation to tumor recurrence and overall survival. Arch. Med. Sci..

[82] Villalva C (2011). STAT3 is essential for the maintenance of neurosphere-initiating tumor cells in patients with glioblastomas: a potential for targeted therapy?. Int J. Cancer.

[83] Tannous BA (2009). Gaussia luciferase reporter assay for monitoring biological processes in culture and in vivo. Nat. Protoc..

[84] Deleyrolle LP (2011). Evidence for label-retaining tumour-initiating cells in human glioblastoma. Brain.

[85] Stanzani, E. et al. Dual role of integrin alpha-6 in glioblastoma: Supporting stemness in proneural stem-like cells while inducing radioresistance in mesenchymal stem-like cells. *Cancers (Basel)* **13**10.3390/cancers13123055 (2021). 10.3390/cancers13123055 PMC8235627 34205341

[86] Zengel P (2011). μ-Slide Chemotaxis: A new chamber for long-term chemotaxis studies. BMC Cell Biol..

[87] Seano G (2019). Solid stress in brain tumours causes neuronal loss and neurological dysfunction and can be reversed by lithium. Nat. Biomed. Eng..

[88] Askoxylakis V (2017). A cerebellar window for intravital imaging of normal and disease states in mice. Nat. Protoc..

[89] Boulay, A. C., Saubaméa, B., Declèves, X. & Cohen-Salmon, M. Purification of Mouse Brain Vessels. *J. Vis. Exp.* e53208 10.3791/53208 (2015). 10.3791/53208 PMC4692710 26574794

[90] Humpel C (2015). Organotypic brain slice cultures: A review. Neuroscience.

[91] Mubeen S (2021). DecoPath: a web application for decoding pathway enrichment analysis. NAR Genom. Bioinform.

[92] Ge SX, Jung D, Yao R (2020). ShinyGO: a graphical gene-set enrichment tool for animals and plants. Bioinformatics.

[93] Thanati, F. et al. FLAME: A Web Tool for Functional and Literature Enrichment Analysis of Multiple Gene Lists. *Biology (Basel)* **10**10.3390/biology10070665 (2021). 10.3390/biology10070665 PMC8301326 34356520

[94] Bray NL, Pimentel H, Melsted P, Pachter L (2016). Near-optimal probabilistic RNA-seq quantification. Nat. Biotechnol..

[95] Melsted P, Ntranos V, Pachter L (2019). The barcode, UMI, set format and BUStools. Bioinformatics.

[96] Lun ATL (2019). EmptyDrops: Distinguishing cells from empty droplets in droplet-based single-cell RNA sequencing data. Genome Biol..

[97] Hao Y (2021). Integrated analysis of multimodal single-cell data. Cell.

[98] Bergen V, Lange M, Peidli S, Wolf FA, Theis FJ (2020). Generalizing RNA velocity to transient cell states through dynamical modeling. Nat. Biotechnol..

[99] La Manno G (2018). RNA velocity of single cells. Nature.

[100] Poullet P, Carpentier S, Barillot E (2007). myProMS, a web server for management and validation of mass spectrometry-based proteomic data. Proteomics.

[101] The M, MacCoss MJ, Noble WS, Kall L (2016). Fast and accurate protein false discovery rates on large-scale proteomics data sets with percolator 3.0. J. Am. Soc. Mass Spectrom..

[102] Valot B, Langella O, Nano E, Zivy M (2011). MassChroQ: a versatile tool for mass spectrometry quantification. Proteomics.

[103] Kuleshov MV (2021). KEA3: improved kinase enrichment analysis via data integration. Nucleic Acids Res.

[104] Wiredja DD, Koyuturk M, Chance MR (2017). The KSEA App: a web-based tool for kinase activity inference from quantitative phosphoproteomics. Bioinformatics.

[105] Perez-Riverol Y (2022). The PRIDE database resources in 2022: a hub for mass spectrometry-based proteomics evidences. Nucleic Acids Res..

[106] Wang, X. et al. Three-dimensional intact-tissue sequencing of single-cell transcriptional states. *Science* **361**10.1126/science.aat5691 (2018). 10.1126/science.aat5691 PMC6339868 29930089

