# Supplementary material for: VC-resist glioblastoma cell state: vessel co-option as a key driver of chemoradiation resistance
Source: Nat Commun. 2024 Apr 29;15:3602. doi: 10.1038/s41467-024-47985-z (PMC11058782; doi:10.1038/s41467-024-47985-z)
Supplement: Supplementary file 1 — Supplementary information [file 41467_2024_47985_MOESM1_ESM.pdf]

# Supplementary Information

## VC-Resist glioblastoma cell state: vessel co-option as a key driver of chemoradiation resistance

Cathy Pichol-Thievend[1,*], Oceane Anezo[1,*], Aafrin M. Pettiwala[1,2,*], Guillaume Bourmeau[1], Remi Montagne[2,3,4], Anne-Marie Lyne[2,3,4], Pierre-Olivier Guichet[5,6], Pauline Deshors[1], Alberto Ballestín[1], Benjamin Blanchard[1], Juliette Reveilles[1], Vidhya M. Ravi[7], Kevin Joseph[7], Dieter H. Heiland[7], Boris Julien[1], Sophie Leboucher[8], Laetitia Besse[9], Patricia Legoix[10], Dingli Florent[11], Stephane Liva[2,3,4], Loew Damarys[11], Elisa Giani[12], Valentino Ribecco[1], Charita Furumaya[1], Laura Kovandzic Marcos[1], Konstantin Masliantsev[5,6], Thomas Daubon[13], Lin Wang[14], Aaron A. Diaz[15], Oliver Schnell[7], Jürgen Beck[7], Nicolas Servant[2,3,4], Lucie Karayan-Tapon[5,6], Florence M.G. Cavalli[2,3,4], Giorgio Seano[1,#]

# Supplementary Figure 1

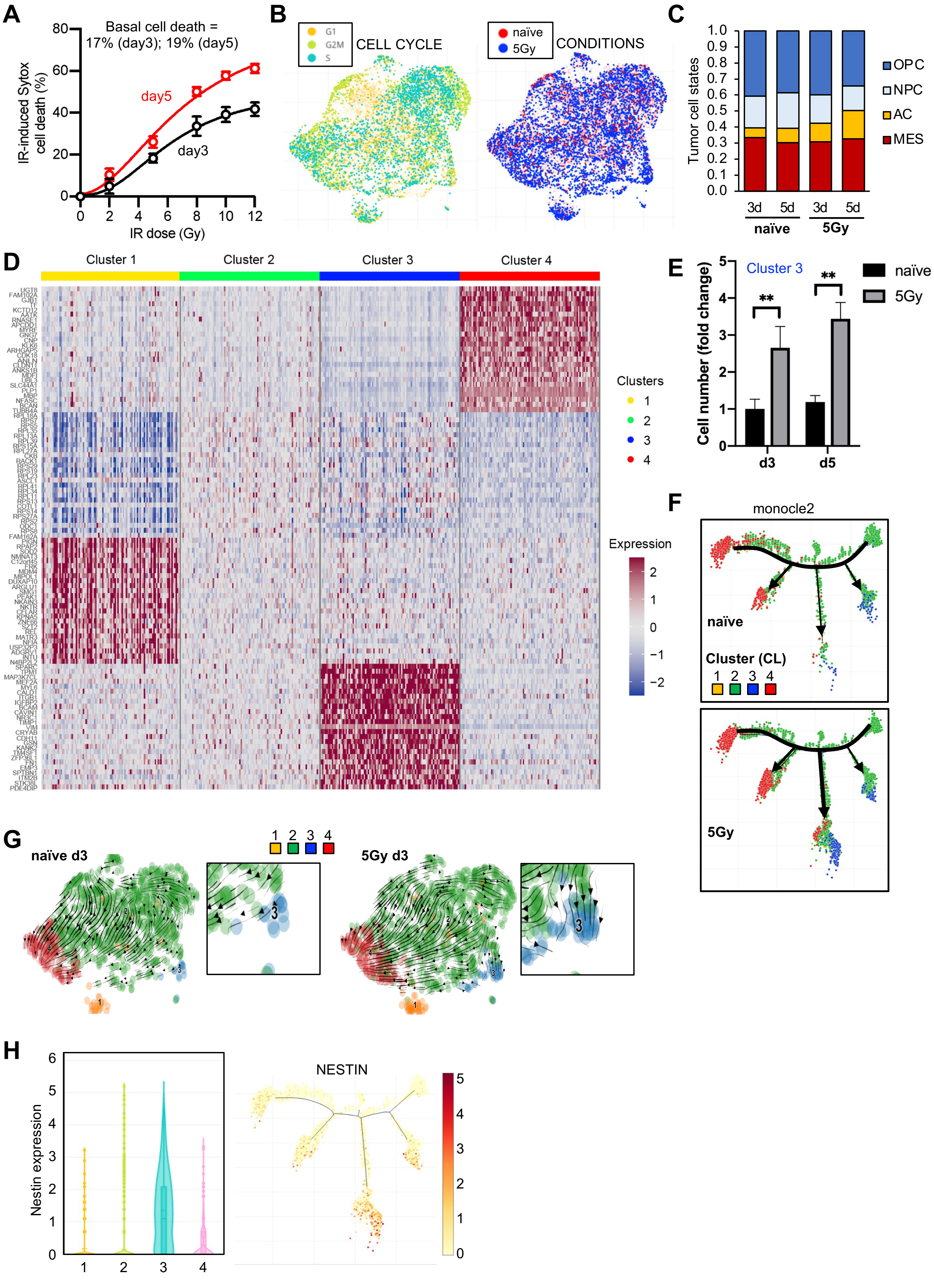

Supplementary Figure 1 – **Time-resolved scRNA-seq dataset upon irradiation**

**A,** Cell death analysis at day 3 and 5 with increasing doses of irradiation in MGG4 cells. **B,** Visualization of the cell cycle phase (**left**) and naïve versus irradiated MGG4 cells features (**right**) in scRNA seq data after dimension reduction (UMAP). **C,** scRNA-seq data of naïve and irradiated MGG4 gliomaspheres at day 3 and 5 analyzed following the pipeline by Neftel et al. Stacked plot representing the distribution in tumor cell states. Astrocyte (AC)-like, oligodendrocyte progenitor cells (OPC)-like, mesenchymal (MES)-like and neural progenitor cells (NPC)-like. **D,** Heatmaps for the genes differentially expressed in the different IKAP clusters (25 genes per cluster). **E,** Fold change for the number of CL3+ cells (naïve vs irradiated conditions). Data are means±SEM (n=2 independent experiments; two-way ANOVA, Tukey's multiple comparisons test). **F,** RNA velocity analysis of MGG4 naïve cells and upon irradiation (5 Gy) at 3 days, showing how IR diverts the trajectories seen in the naive MGG4 cells by generating a new trajectory that leads to CL3**.** **G,** Trajectory analysis of MGG4 in all conditions (day3 and 5, irradiated or not). **H,** Nestin gene expression distribution among clusters (left) and in the trajectory plot (right). Plot made with Cerebro.

# Supplementary Figure 2

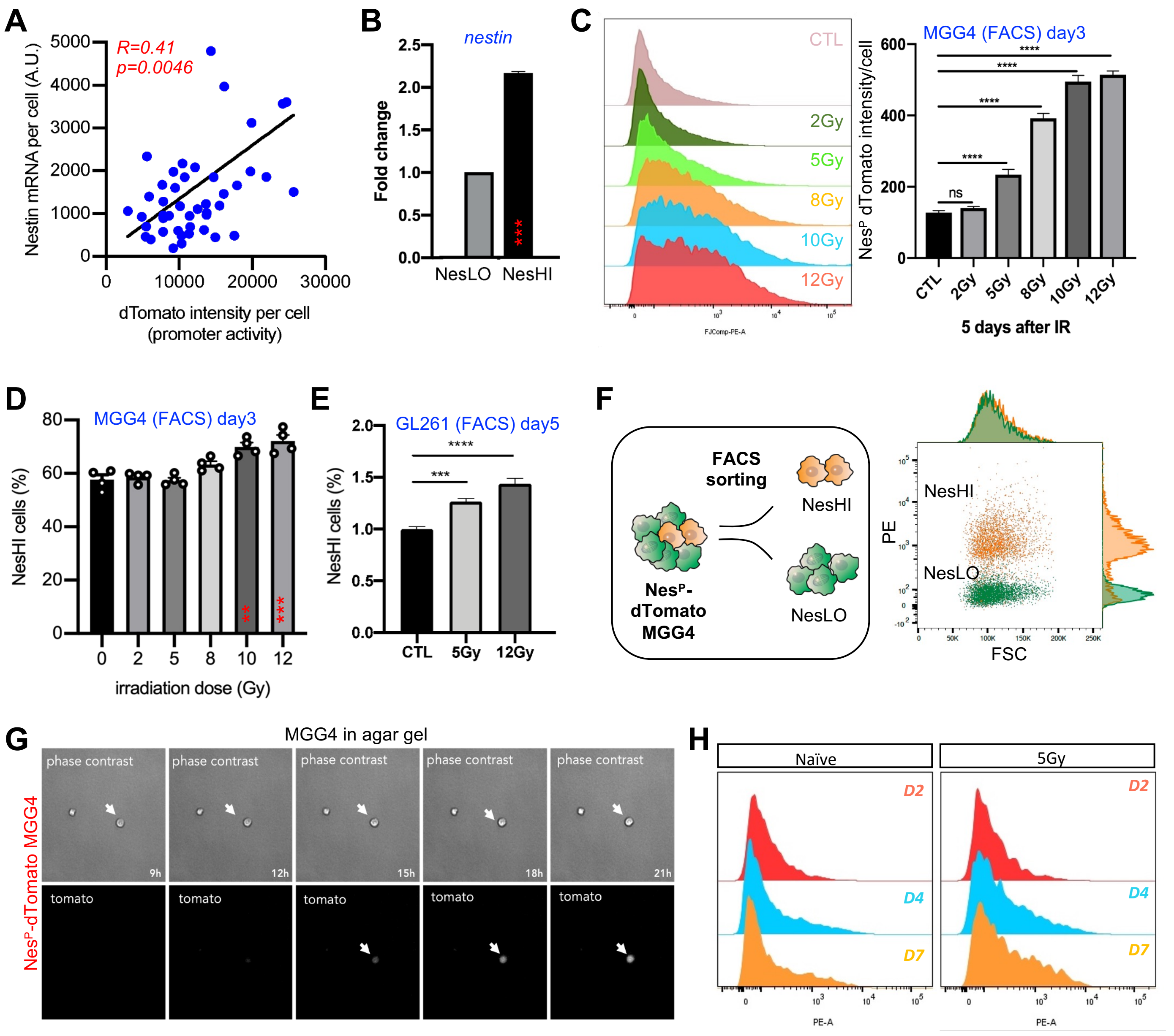

Supplementary Figure 2 – **Tracking cell state transitions unveils that irradiation induces GB cell state transition**

**A,** Correlation analysis between the quantity of Nestin mRNA per cell and the tdtomato intensity per cell in $Nestin^{P}$-dTomato MGG4 cells. **B,** Fold change of NesHI/NesLO quantitative RT-PCR analysis of Nestin expression in MGG4 NesLO and NesHI FACS-sorted cells. Data are means±SEM (n=2). **C,** (**Left**) Flow cytometry histograms of Tomato intensity in $Nestin^{P}$-dTomato MGG4 cells irradiated (2, 5, 8, 10,12Gy) or not (control). (**Right**) Tomato intensity increase under escalading doses of irradiation (2, 5, 8, 10, 12Gy) at day 3 in $Nestin^{P}$-dTomato MGG4 cells. Data are means±SEM (n=4, ns, non-significant; ****p < 0.0001). **D,** Enrichment of NesHI cell population upon irradiation (2, 5, 8, 10, 12Gy) analyzed at day 3 by FACS in $Nestin^{P}$-dTomato MGG4 cells. Data are means±SEM (n=4). **E,** Enrichment of NesHI cell population upon irradiation (5 and 12Gy) in $Nestin^{P}$-dTomato GL261 cells analyzed at day 3 by FACS. Data are means±SEM. **F,** FACS-sorting strategy of NesLO and NesHI cells and corresponding flow cytometry histogram profiles. **G,** Real-time microscopy images of FACS-sorted MGG4 NesLO cells seeded in agar gel, showing the induced NesLO-to-NesHI transitions and detected using the Nestin reporter. The arrow indicating reprogramming. **H,** Flow cytometry histograms of Tomato intensity in MGG4 NesLO cells irradiated (5Gy) or not (naïve) at day 2, 4 and 7. Tomato intensity increases upon irradiation.

# Supplementary Figure 3

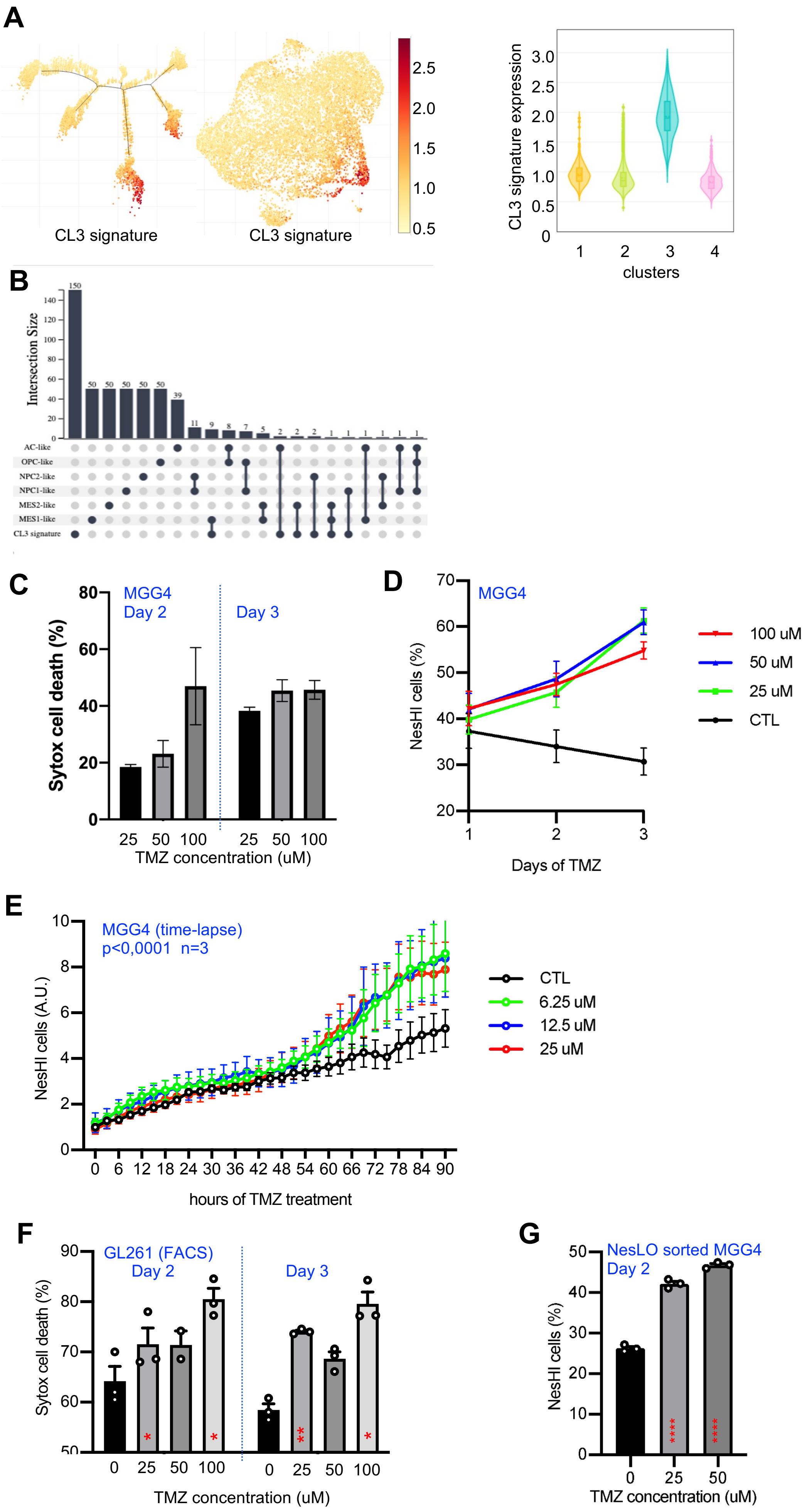

Supplementary Figure 3 – **Temozolomide induces GB reprogramming towards the newly discovered IR-induced cell state**

**A,** Feature plot and violin graph of CL3 geneset expression in the scRNA-seq dataset from Fig. 1B. CL3 signature is composed by the 150 most significantly upregulated genes in CL3 vs the rest of the cells. Plot made with Cerebro. **B,** UpSet plot showing the lack of significant overlap of the CL3 geneset with the Neftel's GB classifiers. **C,** Cell death analysis at day 2 and 3 with increasing doses of TMZ in MGG4 cells. Data are means±SEM (n=3) **D,** Enrichment of NesHI cells control and treated by TMZ (25uM to 100 uM) analyzed by FACS in $Nestin^{P}$-dTomato MGG4 cells overtime. Data are means±SEM. **E,** Enrichment of NesHI cell population overtime upon TMZ treatment (6,25, 12,5 and 25uM) in $Nestin^{P}$-dTomato MGG4 cells analyzed by real-time microscopy. Data are means±SEM. **F,** Enrichment of NesHI cell population under TMZ treatment (25, 50 and 100uM) analyzed by FACS in $Nestin^{P}$-dTomato GL261 cells overtime. Data are means±SEM. **G,** Enrichment of NesHI cell population upon TMZ treatment in FACS-sorted MGG4 NesLO cells (right) analyzed at day 2 by FACS. Data are means±SEM (n=3; *p<0.05; ****p<0.0001; one-way ANOVA, Tukey's multiple comparisons test).

# Supplementary Figure 4

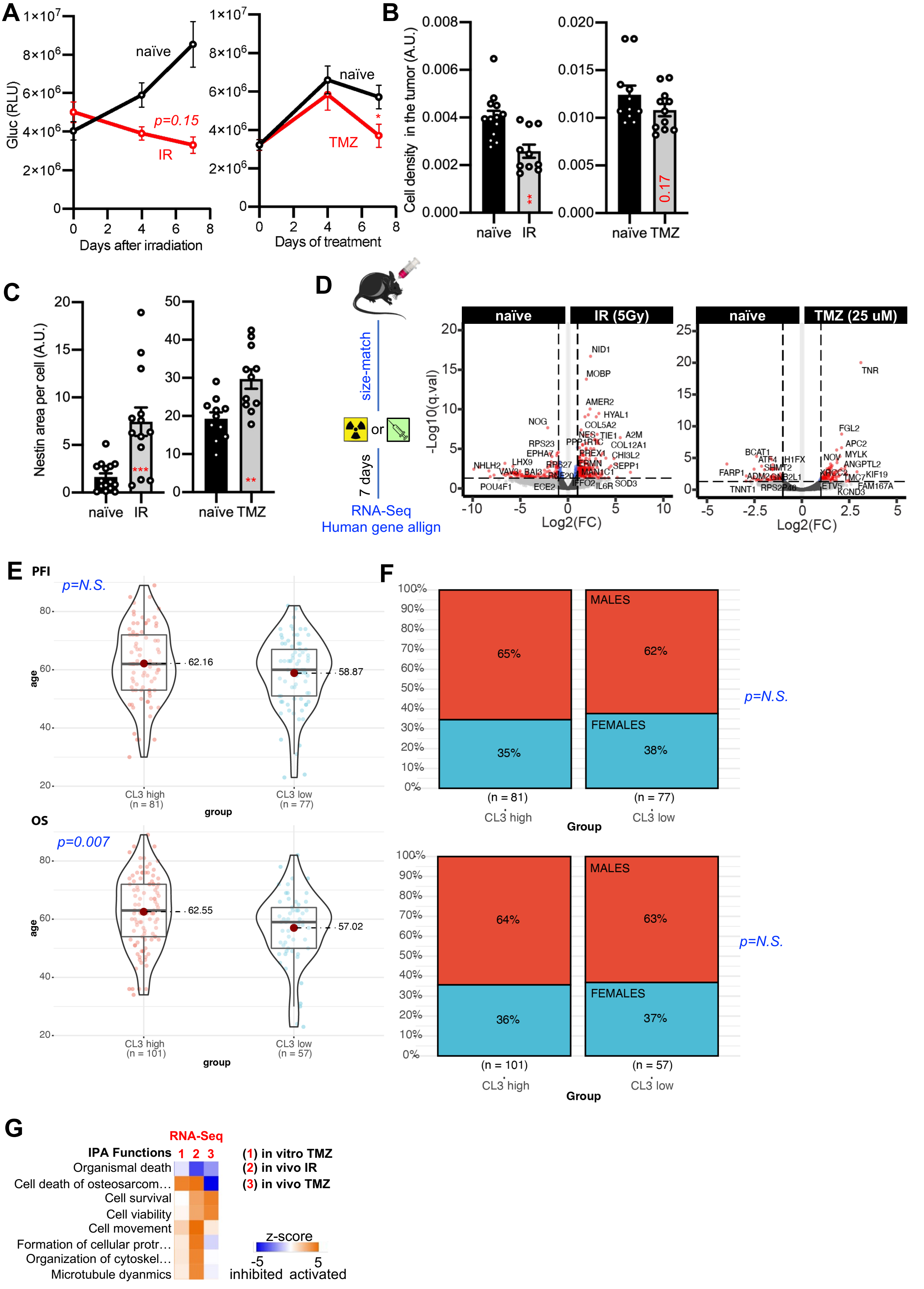

Supplementary Figure 4 – **Preclinical and clinical validation of the therapy-induced functional GB state**

**A,** *In vivo* Gaussia Luciferase (Gluc) activity of MGG4-GFP-Gluc cells intracranially injected in immunocompromised mice over time post irradiation (10Gy) and TMZ (10mg/kg); n=15 naïve, n=13 IR (10Gy); n=11 naïve (DMSO), n=11 TMZ (10 mg/kg); *p<0,05 at day 7, unpaired two-sided t-test. **B,** Bar plots showing quantification of cellular density of MGG4 cells intracranially-injected in immunocompromised mice over time post irradiation (10Gy) and TMZ (10mg/kg) respectively. Data are means±SEM; n=15 naïve, n=13 IR (10Gy); n=11 naïve (DMSO), n=11 TMZ (10 mg/kg); p<0,05, unpaired two-sided t-test. **C,** Bar plots showing quantification of Nestin area/cell of MGG4 cells injected intracranially in immunocompromised mice over time post irradiation (10Gy) and TMZ (10mg/kg) respectively. Data are means±SEM; n=15 naïve, n=13 IR (10Gy); n=11 naïve (DMSO), n=11 TMZ (10 mg/kg); p<0,05, unpaired two-sided t-test. **D,** Volcano plots of the differential expression analysis between in vivo MGG4 naïve and irradiated (**left**), as well as in vivo MGG4 naïve and treated with TMZ (**right**). Horizontal dotted bar represents a significance level of p=0.05. Vertically the bar is set at a fold change of 2. **E-F,** Overall survival and progression-free interval prognostic index estimation in TCGA-GBM database (only the IDH-wt patients selected). Age and gender were not different in the two groups. **G,** Biological functions predicted by Ingenuity Pathway Analysis (IPA) by using the differentially expressed genes in *in vitro* TMZ, *in vivo* IR or TMZ-treated MGG4 cells or tumors. Z-score indicates the predicted level of activation (if positive) or inhibition (if negative) of the function. All functions shown are statistically significant.

# Supplementary Figure 5

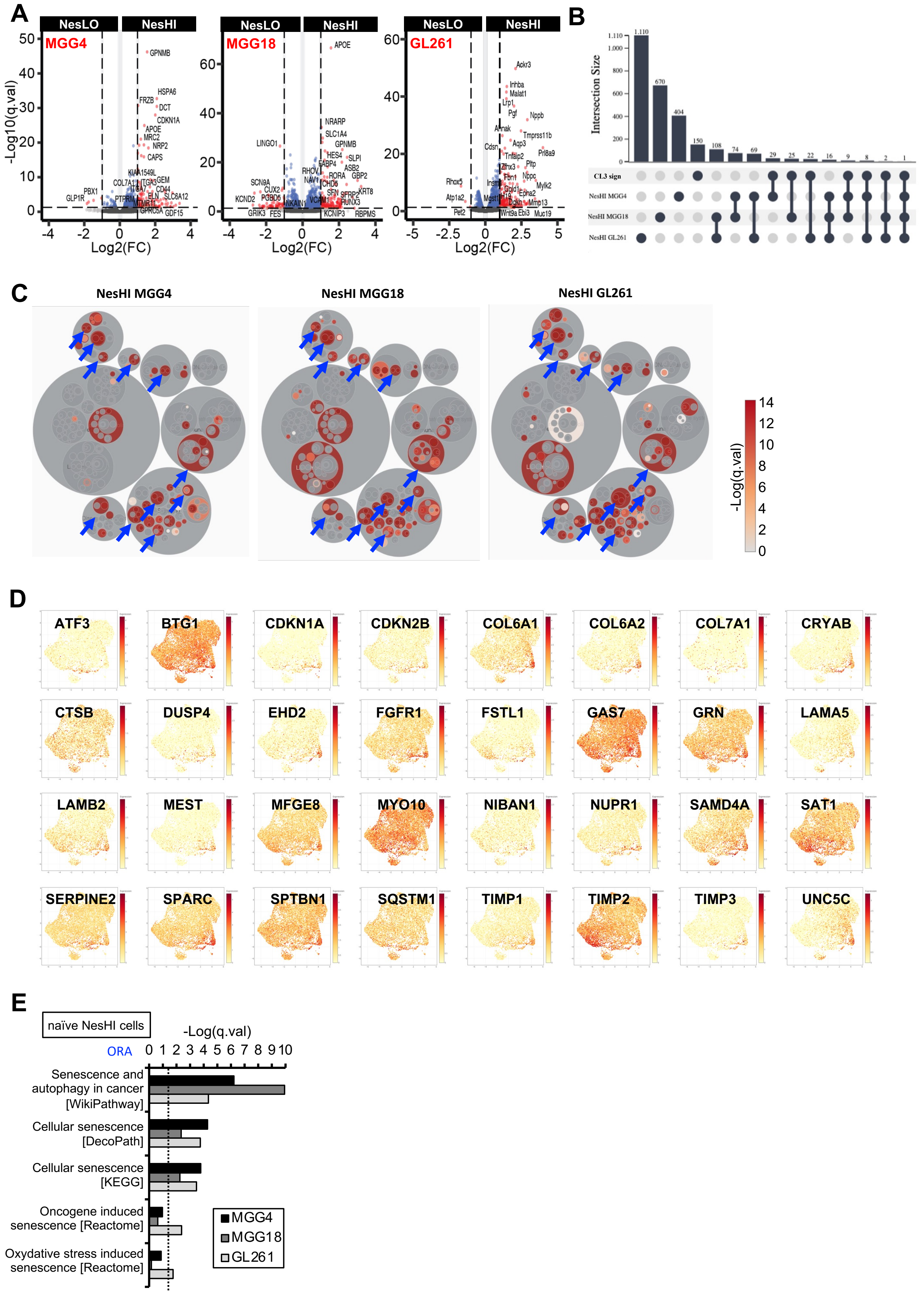

Supplementary Figure 5 – **The 32 genes commonly upregulated in the NesHI subpopulations**

**A,** Volcano plots of the differential expression analysis between NesLO and NesHI cells in FACS-sorted $Nestin^{P}$-dTomato MGG4, MGG18 and GL261 cells. Horizontal dotted bar represents a significance level of p=0.05. Vertically, the bar is set at a fold change of 2. **B,** Genes in common in CL3/NesHI genesets. UpSet and Venn graphs of CL3 geneset, NesHI MGG4, MGG18 and GL261 upregulated genes. **C,** Over-representation analysis for the upregulated genes in NesHI MGG4, MGG18 and GL261 cells. The graph shows the -Log(q.val) for the most relevant senescence pathways (performed with DecoPath). **D,** Feature plots for each of the 32 common genes in the NesHI/CL3 datasets. Plots made with Cerebro. **E,** Over-representation analysis for NesHI MGG4, MGG18 or GL261 upregulated genes. The significantly over-represented pathways are colored and specified (performed with DecoPath).

# Supplementary Figure 6

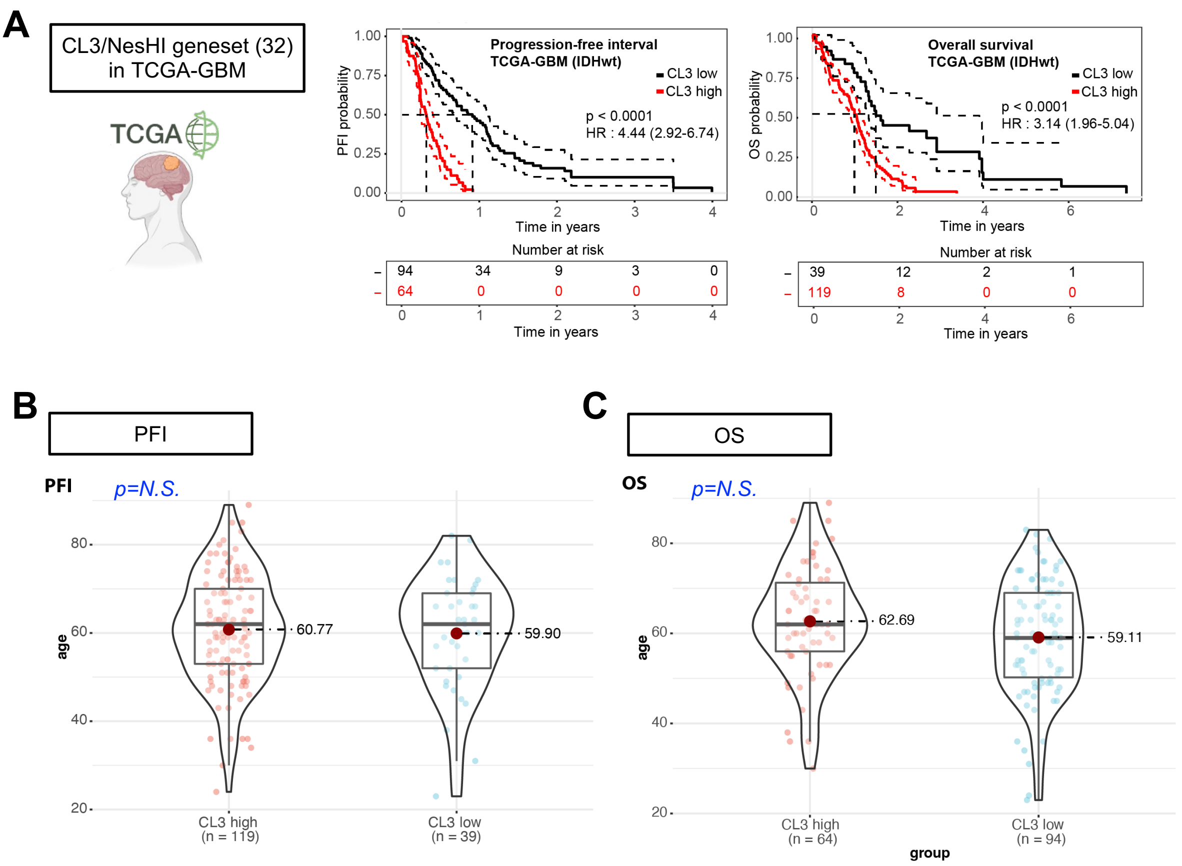

Supplementary Figure 6 – **The 32 genes commonly upregulated in the NesHI subpopulations are prognostic for patients in the TCGA dataset**

**A,** Overall survival and progression-free interval prognostic index estimation in TCGA-GBM database (only the IDH-wt patients selected). Using the Tumor online Prognostic analyses Platform (ToPP), we perform a multivariate analysis with Cox proportional hazards model. CL3/NesHI common gene signature (32 genes) was used to stratify patients ("best cut"). Age and gender were not different in the two groups. **B-C,** Age across the groups.

# Supplementary Figure 7

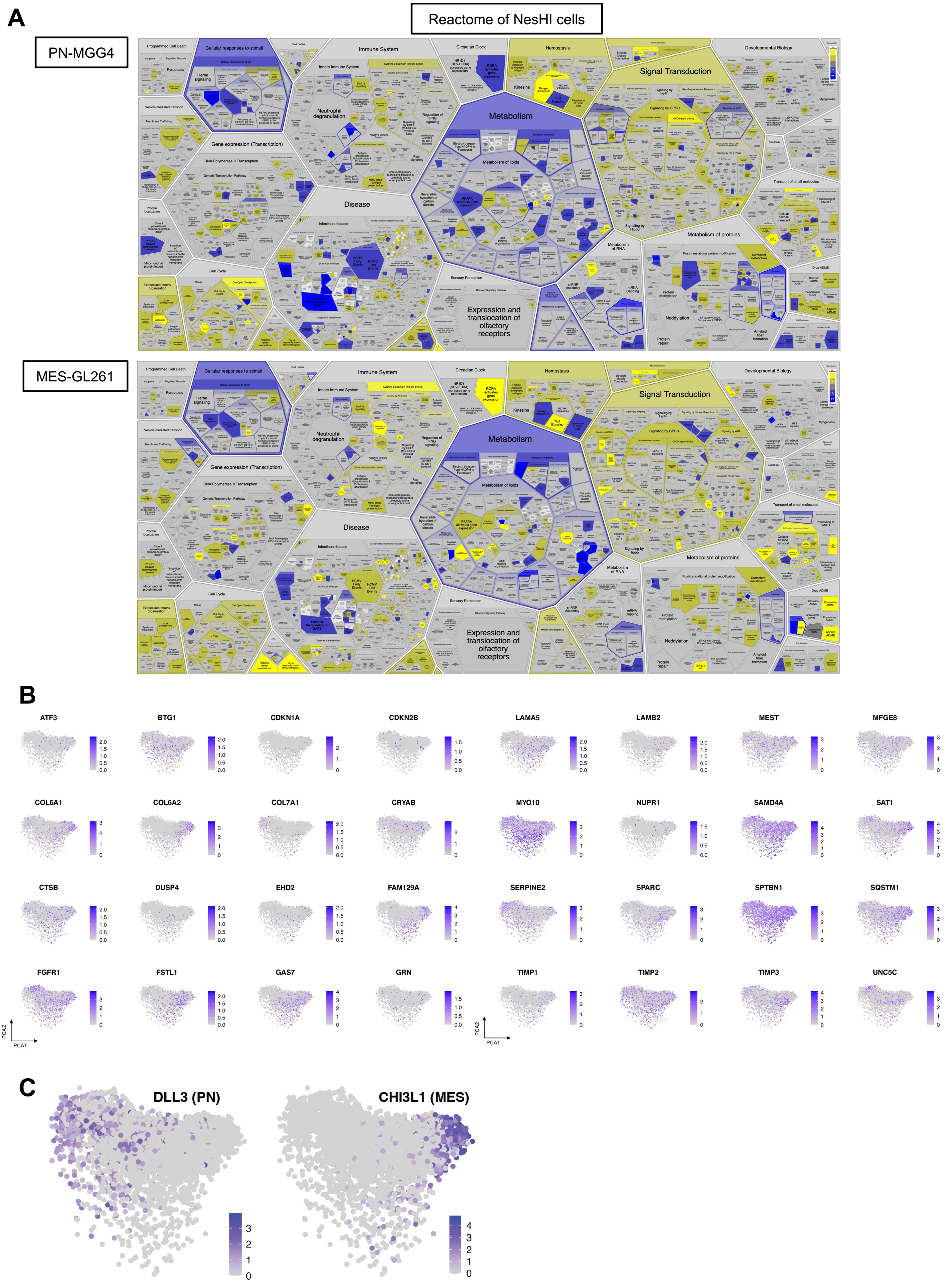

Supplementary Figure 7 – **GSEA for NesHI subpopulations**

**A,** Over-representation analysis of NesHI patient-derived GB cells using the Reactome database. The whole RNA-Seq result was used to perform the PADOG analysis (i.e., a weighted geneset analysis method that down-weighs genes that are present in many pathways) between NesLO and NesHI-sorted cells. **B,** Feature plots for each of the 32 common genes in the NesHI/CL3 datasets in the PMT dataset from Fig. 4E. **C,** Feature plots for the markers of the PN (DLL3) and MES (CHI3L1) subtypes.

# Supplementary Figure 8

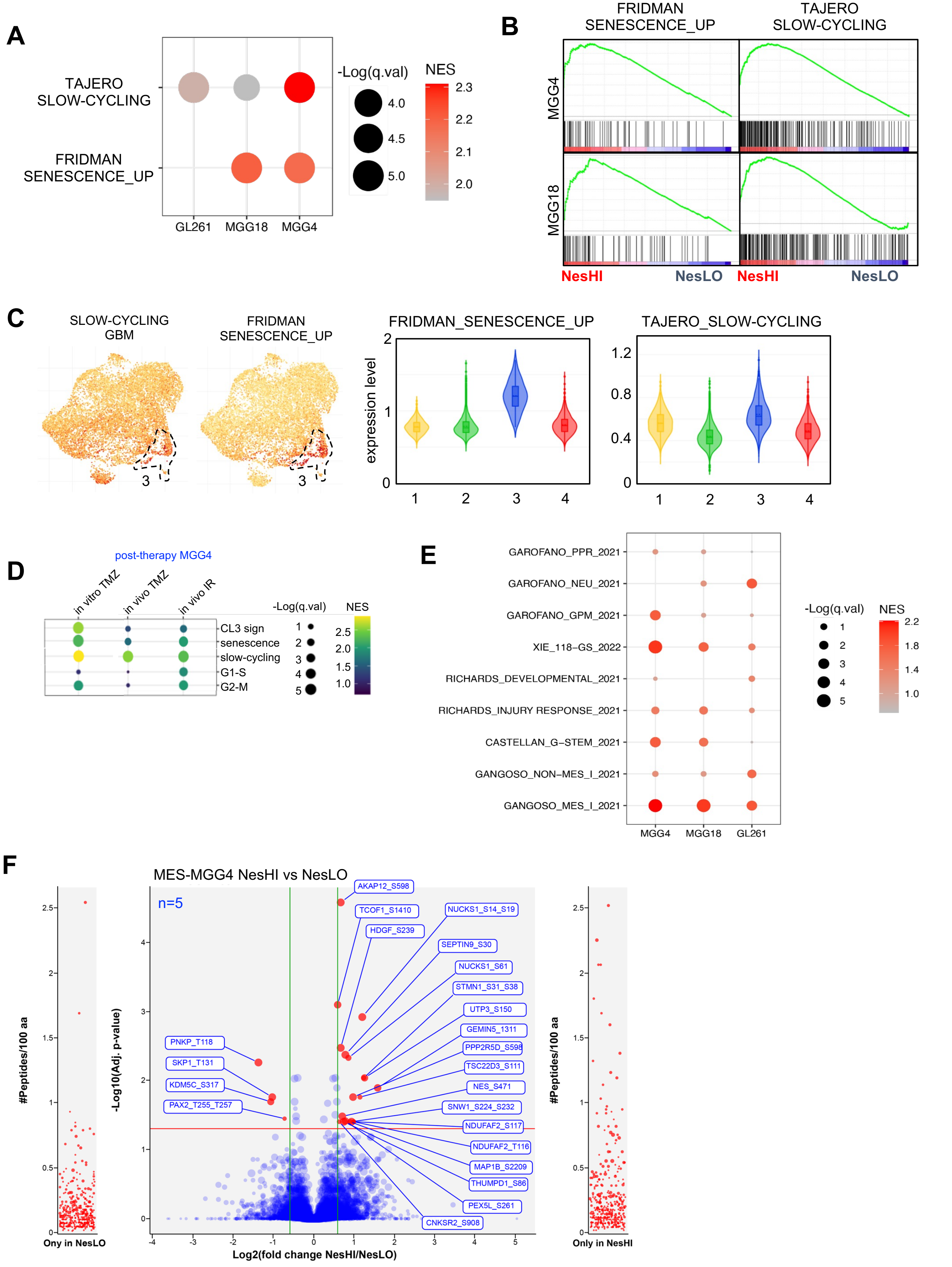

Supplementary Figure 8 – **GSEA for NesHI subpopulations**

**A,** Bubble plot of "slow-cycling" and senescence gene sets in NesHI cells in MGG4, MGG18 and GL261 cell lines. Color code and size indicate NES and significance respectively. **B,** GSEA plots for senescence and "slow-cycling" gene sets in the NesHI and NesLO MGG4 or MGG18 cells. The normalized enrichment score (NES) and q.value are indicated. **C,** Distribution in the UMAP (**left**) and violin plot (**right**) of the enrichment of FRIDMAN_SENESCENCE_UP and TEJERO_SLOW-CYCLING genesets in the 4 clusters of the scRNA-seq dataset from Fig. 1B. **D,** Bubble plot of the indicated gene sets in MGG4 cell line treated with TMZ or irradiation *in-vivo*, and TMZ *in-vitro*. Color code and size indicate NES and significance respectively. **E,** Bubble plot of the indicated published signatures in NesHI MGG4, MGG18 and GL261 cells. Color code and size indicate NES and significance respectively. **F,** Volcano plot for phosphoproteome analysis between NesHI and NesLO-sorted PN-MGG4 cells. Horizontal dotted bar represents a significance level of p=0.05. Vertically the bar is set at a fold change of 2. Proteins specific to one of the two groups compared were assigned a fold change of infinity ("only in NesHI" or "only in NesLO" sections). Only proteins with at least 3 total peptides in all replicates (n=3) over 5 biological replicates were considered significantly enriched in sample comparisons.

# Supplementary Figure 9

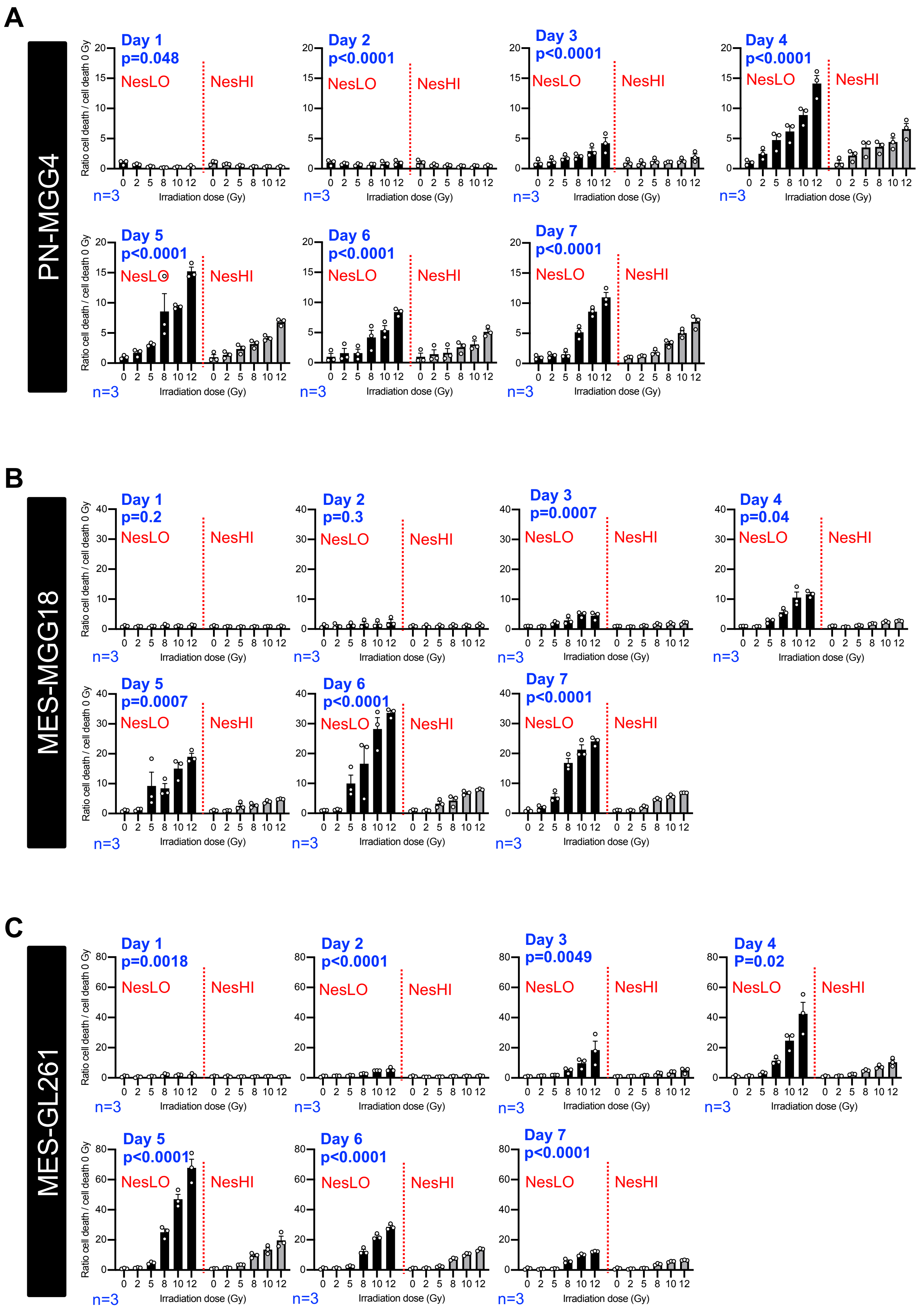

Supplementary Figure 9 – **NesHI cells are more resistant to cell death after irradiation**

Dose-response graphs. Cell death analysis in NesLO vs NesHI cells under escalading doses of irradiation (2, 5, 8, 10, 12 Gy) overtime (7 days), in Nestin$^{P}$-dTomato MGG4 cells (**A**), MGG18 cells (**B**), and GL261 cells (**C**). Data are means±SEM. (n=3, p-values are shown, Anova test between the two cell populations). Fold change of the percentage of irradiated cells normalized by the percentage of controls for each day.

# Supplementary Figure 10

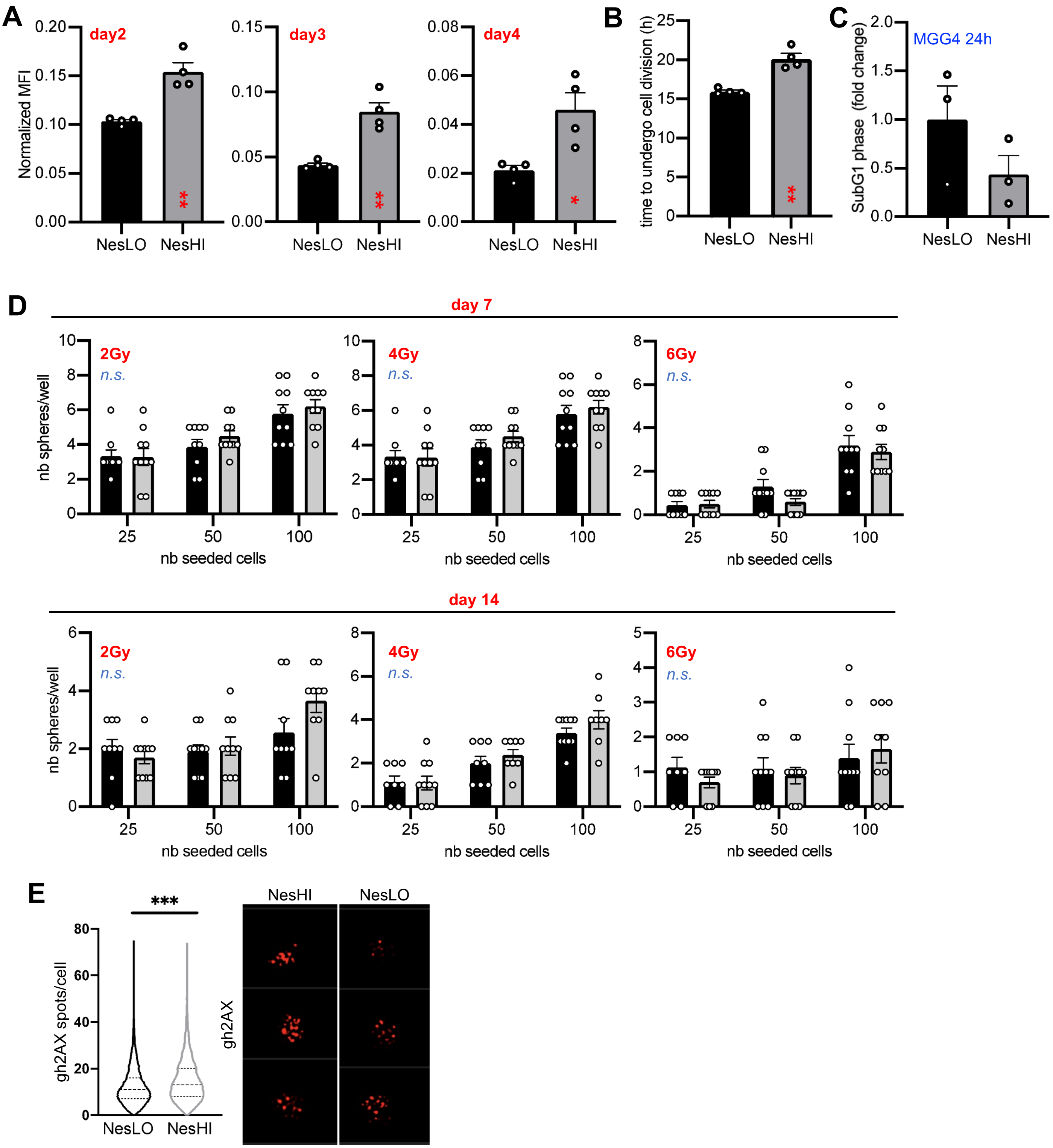

Supplementary Figure 10 – **NesHI cells are more resistant and slow-cycling**

**A,** FACS analysis of CellTrace$^{TM}$ dye dilution during cell division in MGG4 NesLO and NesHI cells. The normalized mean fluorescent intensity of CellTrace$^{TM}$ dye is higher in NesHI cells compare to NesLO cells at day 2, 3 and 4. Data are means±SEM (n=4, *p<0.05, **p<0.01). **B,** Time to undergo cell division was calculated based on the mean fluorescent intensity values in NesLO vs NesHI cells and is higher in NesHI cells. Data are means±SEM (n=4, **p < 0.01). **C,** Bar plots showing decrease in SubG1 cell cycle phase in NesHI cell subpopulation compared to NesLO cell population in Nestin$^{P}$-dTomato MGG4 cells at day 1. Data are means±SEM (n=3, *p<0,05, **p < 0.01, paired two-sided t-test). **D,** Clonogenic assay performed in FACS-sorted MGG4 NesLO (black bars) and NesHI (white bars) cells with escalading doses of irradiation (2, 4 and 6 Gy). The number of spheres scored at day 7 and 14 were plotted respectively to the number of cells seeded. **E,** (**Left**) Violin plot illustrating γ−H2AX spots/cell in MGG4 NesHI and NesLO cells. Data are means±SEM (n=3, total number of cells quantified are > 6000, ***p<0.001, unpaired two-sided t-test). (**Right**) Representative images of γ−H2AX foci in MGG4 NesHI and NesLO.

# Supplementary Figure 11

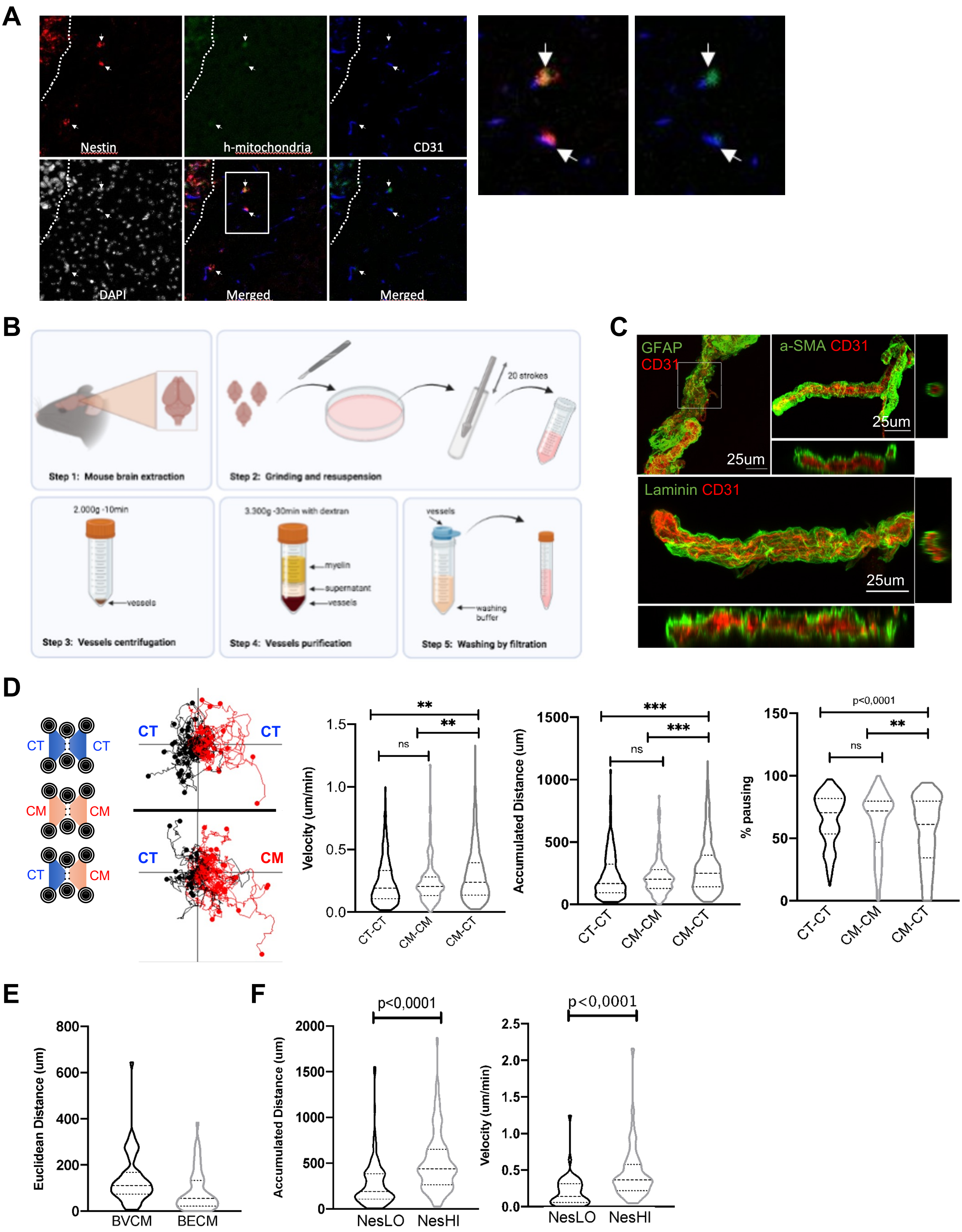

Supplementary Figure 11 – **The perivascular niche in a dish model and chemotaxis**

**A, (Left)** Immunofluorescence staining of Nestin, human-mitochondria, CD31 (blood vessels) of a MGG4-tumor-bearing mouse brain irradiated with 10Gy. The arrows shows Nestin+human-mitochondria+ cells close to blood vessel (co-option). **(Right)** magnification of two vessel co-opting Nestin+, hMito+ MGG4 cells. **B,** Workflow for the mouse brain vessel isolation **C,** Maximum projection of confocal images and orthogonal views of immunostaining performed on isolated brain vessels with CD31 (endothelial cells), α-SMA (smooth muscle actin), GFAP (astrocyte). Scale bar, 25um **D,** (**Left**) Trajectory plots of $Nestin^{P}$-dTomato MGG4 cells in response to bEnd.3 conditioned media (CM) vs control (CT) condition, (n=3). (**Right**) Violin plots showing an increase in velocity, accumulated distance (um) and decrease in the percentage of pausing of $Nestin^{P}$-dTomato MGG4 cells in CM-CT vs CT-CT and CM-CM conditions, Data are means±SEM (n=3, total number of cells quantified are > 135; ns, non-significant; **p<0.01, ***p<0.001, one-way ANOVA, Tukey's multiple comparisons test). **E,** Violin plots showing the Euclidean distance (um) of bulk MGG4 cells in Blood vessel conditioned media vs b.End.3 conditioned media. **F,** Violin plots showing increase in accumulated distance and velocity of FACS-sorted NesHI and NesLO MGG4 cells in response to bEnd.3 conditioned media (CM). Data are means±SEM. (n=3, total number of cells quantified are > 65, ***p<0.0001, one-way ANOVA, Tukey's multiple comparisons test).

# Supplementary Figure 12

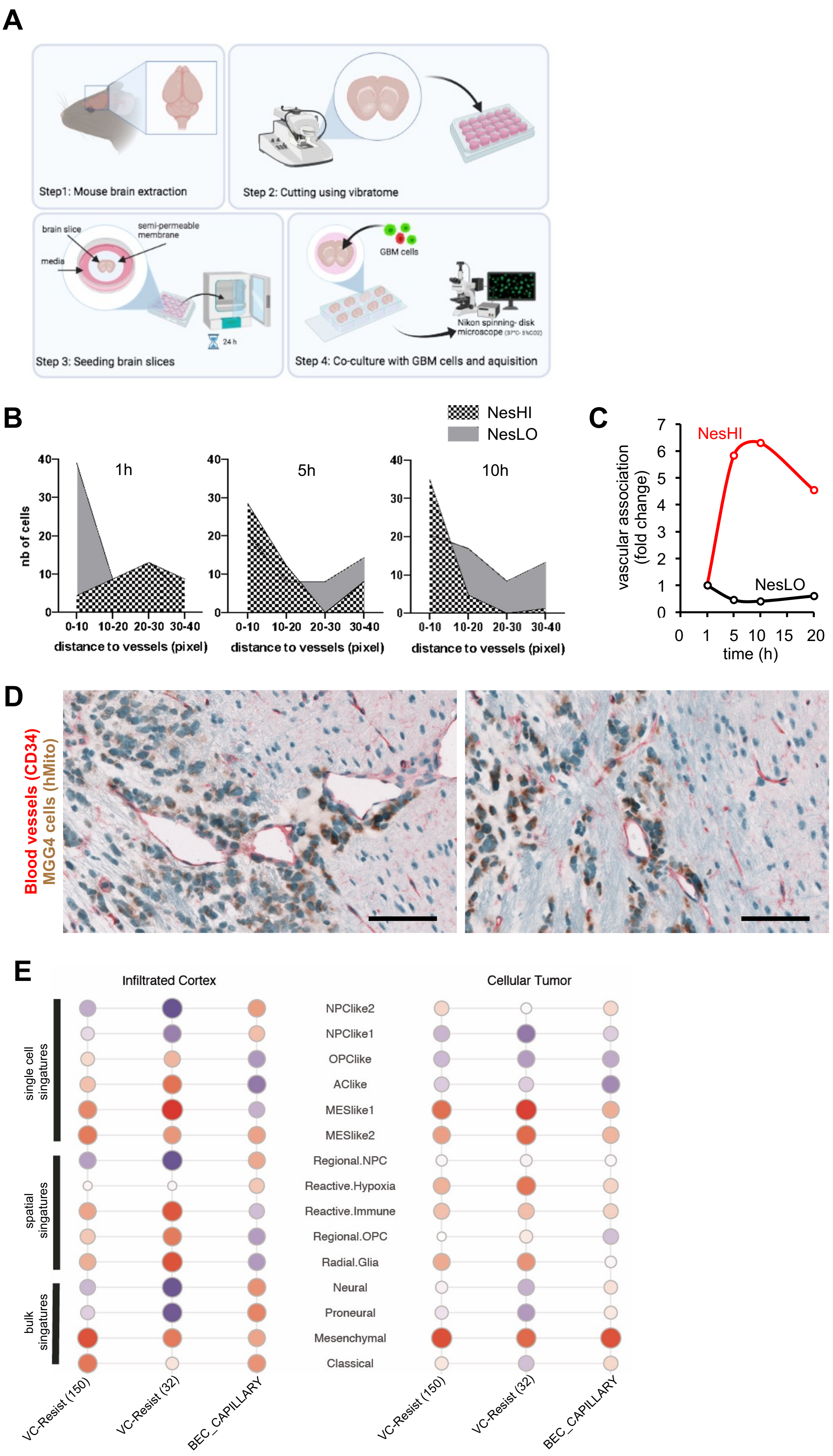

Supplementary Figure 12 – **Vessel co-option in brain slices and preclinical models**

**A,** Pipeline for the brain slice organotypic model. **B,** Comparison of the distribution of NesHI and NesLO cells on brain slices according to their distance to vessels over time. Followed cells number was between 69 to 120, depending on the time point. **C,** Fold change of the vascular association of NesHI cells or NesLO cells at different time points. Vascular association means the number of cells present within 10um of a blood vessel at 4 different time points. Data are from tracking of more than 100 cells. **D,** Vessel co-option and vascular it is blurred satellitosis in the invasive fronts of MGG4 intracranial tumors. Representative micrograph for immunostaining of tumor cells with anti-hMito (brown) close to CD34 stained blood vessels (red). Scale bar, 20 µm. **E,** Bubble plot of spatially weighted correlations across VC-Resist (150 genes), VC-Resist (32 genes) and GB classifiers' genesets for n=3 patients in both infiltrative cortex and cellular tumor. Spatially weighted correlation is color-coded.

# Supplementary Figure 13

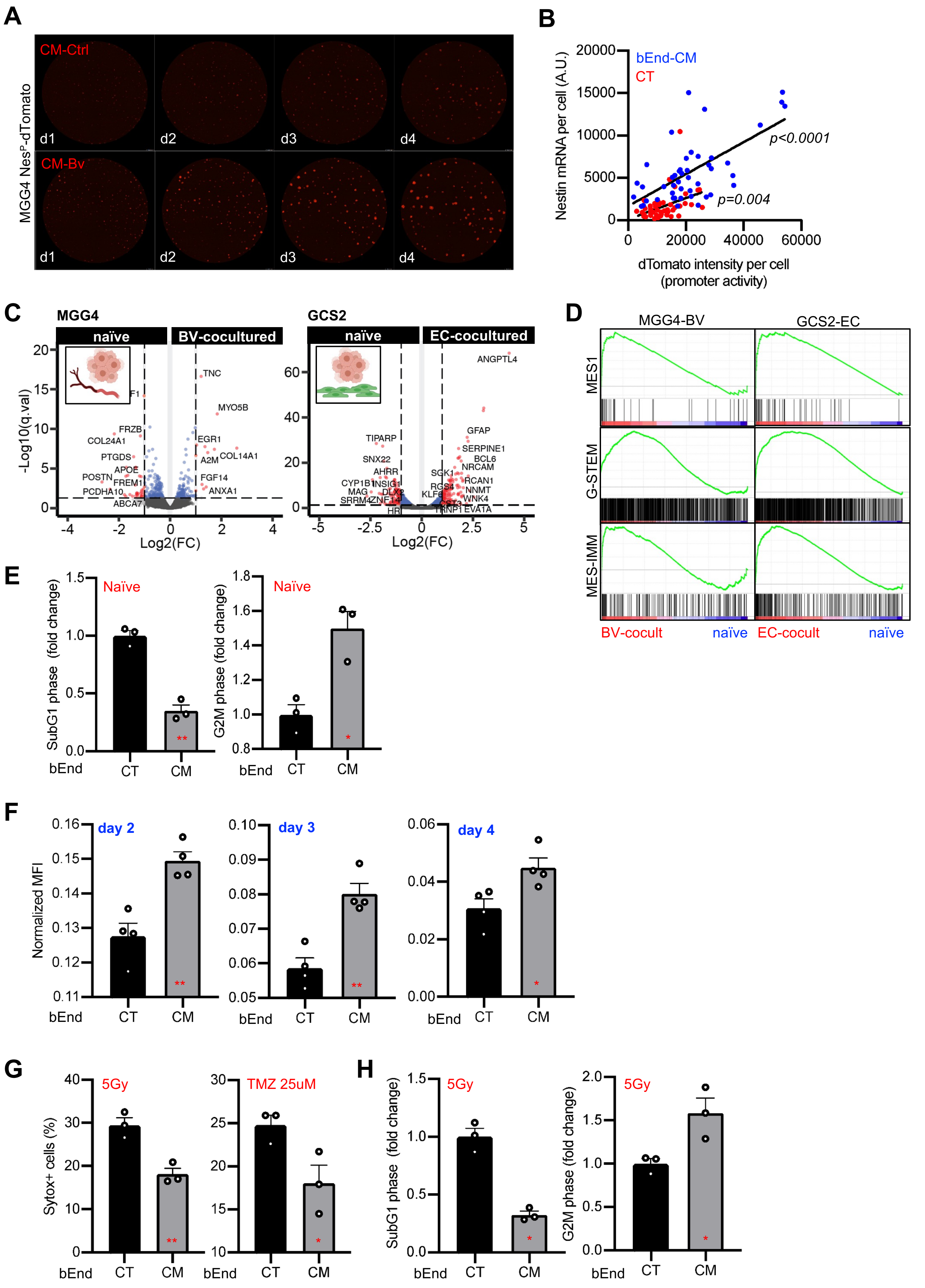

Supplementary Figure 13 – **Blood vessel co-culture induces the VC-Resist cell state**

**A,** Representative images showing the reprogramming of MGG4 cells in presence of conditioned media from blood vessels over time using incucyte live-cell imaging system. **B,** Pearson correlation analysis between the quantity of Nestin mRNA per cell and the dTomato intensity per cell in $Nestin^{P}$-dTomato MGG4 cells in the presence of bEnd.3 conditioned media (bEnd-CM) or control media (CT). **C,** Volcano plot of the differential expression analysis between (**Left**) MGG4 naïve and co-cultured with mouse blood vessels and (**Right**) GSC2 co-cultured with endothelial cells. Horizontal dotted bar represents a significance level of p=0.05. Vertically, the bar is set at a fold change of 2. **D,** GSEA plots of indicated published signatures in co-cultured MGG4 and GSC2 cells. The NES and q.value are indicated. **E,** Bar plots showing decrease in SubG1 and increase in G2M cell cycle phases for $Nestin^{P}$-dTomato MGG4 cells preconditioned with bEnd.3-CM or control (CT); Data are means±SEM (n= 3; *p<0,05; **p<0.01; unpaired two-sided t-test). **F,** FACS analysis of $CellTrace^{TM}$ dye dilution during cell division in in $Nestin^{P}$-dTomato MGG4 cultivated in CM-bEnd (CM) or control (CT) media. The normalized mean fluorescent intensity of $CellTrace^{TM}$ dye is higher in CM compared to CT conditions at day 2, 3 and 4. Data are means±SEM (n=4, *p<0.05, **p<0.01). **G,** Cell death (percentage of Sytox+ cells) in $Nestin^{P}$-dTomato MGG4 cells pre-conditioned with bEnd.3 conditioned media (CM) vs control (CT) in response to irradiation (5Gy) and TMZ (25 µM); Data are means±SEM (n=3; **p<0.01; ***p<0.001; unpaired two-sided t-test). **H,** Decrease in SubG1 and increase in G2M cell cycle phases in $Nestin^{P}$-dTomato MGG4 cells pretreated with bEnd.3 conditioned media (CM) vs control (CT) in response to irradiation (5Gy). Data are means±SEM (n=3; *p<0,05; unpaired two-sided t-test).

# Supplementary Figure 14

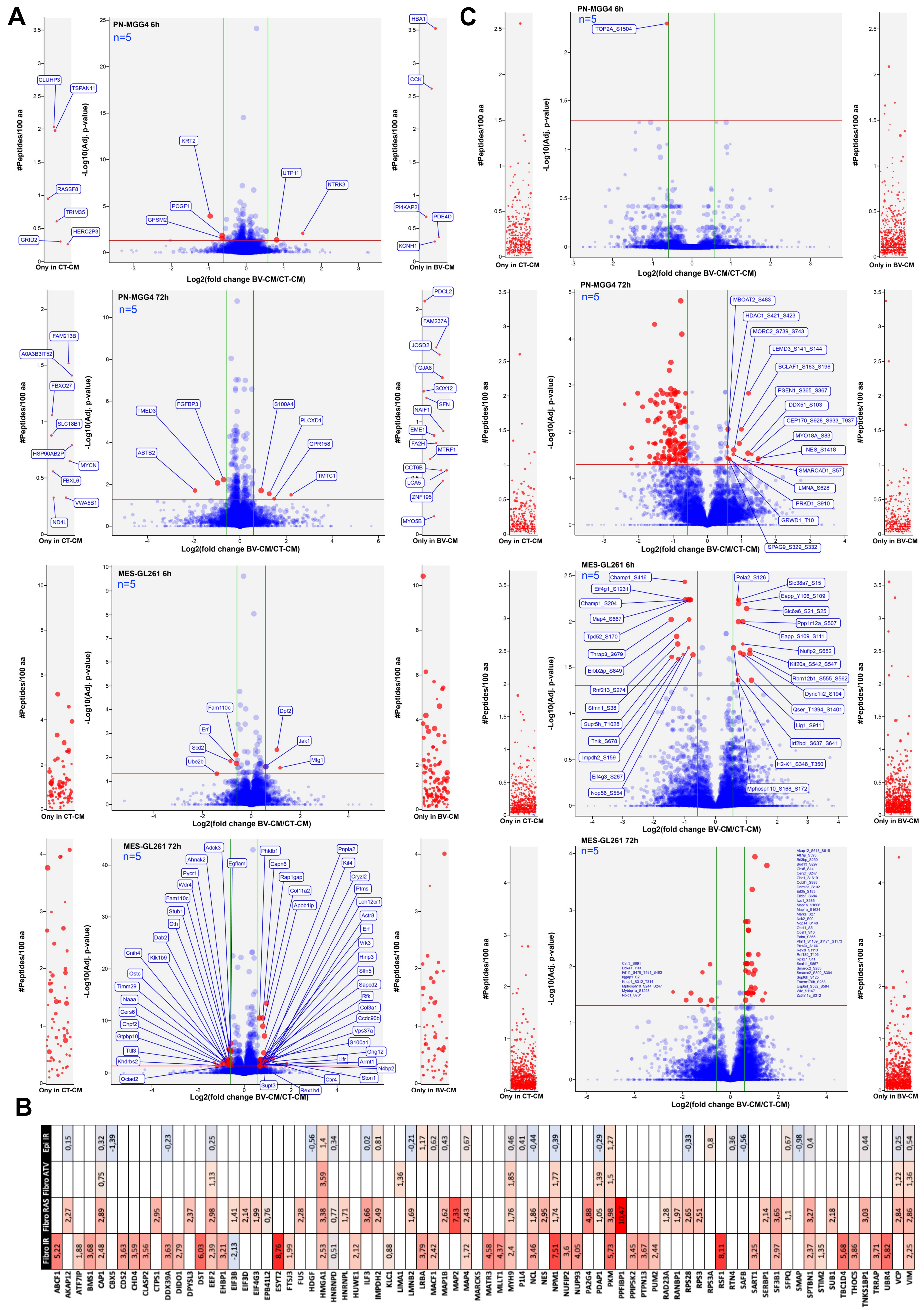

Supplementary Figure 14 – **Proteome and phosphoproteome analysis of BV-CM treated PN-MGG4 and MES-GL261 GB cells.**

**A,** Volcano plot for proteome analysis between the patient-derived PN-MGG4 or the mouse MES-GL261 treated with control (CT-CM) or blood vessel condition media (BV-CM) for 3 days. Horizontal dotted bar represents a significance level of p=0.05. Vertically the bar is set at a fold change of 2. Proteins specific to one of the two groups compared were assigned a fold change of infinity ("only in CT-CM" or "only in BV-CM" sections). Only proteins with at least 3 total peptides in all replicates (n=3) over 5 biological replicates were considered significantly enriched in sample comparisons. **B,** SASP Atals analysis (http://www.saspatlas.com) of the commonly upregulated proteins from Fig. 7K. Only the proteins present in the SASP Atlas are shown. X-ray irradiation, fibroblast or epithelial cells (genotoxic stress-induced; Fibro IR and Epi IR); RAS overexpression (oncogene-induced; Fibro RAS); Atazanivir treatment (treatment-induced; Fibro ATV). **C,** Volcano plot for phosphoproteome analysis between the patient-derived PN-MGG4 or the mouse MES-GL261 treated with control (CT-CM) or blood vessel condition media (BV-CM) for 3 days. Horizontal dotted bar represents a significance level of p=0.05. Vertically the bar is set at a fold change of 2. Proteins specific to one of the two groups compared were assigned a fold change of infinity ("only in CT-CM" or "only in BV-CM" sections). Only proteins with at least 3 total peptides in all replicates (n=3) over 5 biological replicates were considered significantly enriched in sample comparisons.

# Supplementary Figure 15

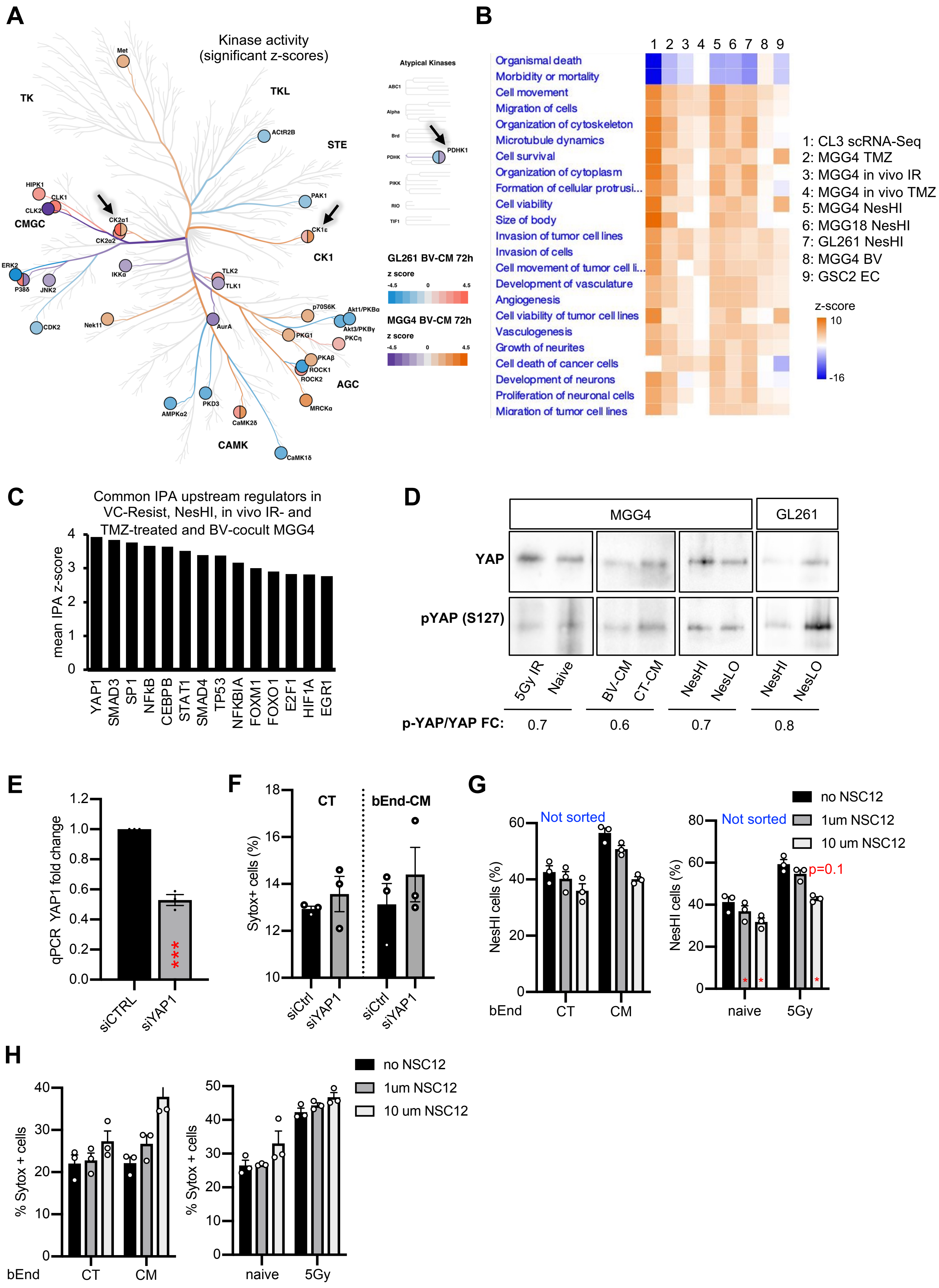

Supplementary Figure 15 – **YAP activation in cell state transitions**

**A,** Phylogenic tree for kinase activity enrichment of the phosphoproteome in PN-MGG4 and MES-GL261 after 72h with BV-CM. Created with the CORAL web-based platform (http://phanstiel-lab.med.unc.edu/CORAL/). **B,** Biological functions predicted by Ingenuity Pathway Analysis (IPA) by using the differentially expressed genes in all datasets and conditions studied in the paper. Z-score indicates the predicted level of activation (if positive) or inhibition (if negative) of the function. All functions shown are statistically significant. **C,** Common IPA upstream transcriptional factors that induces the VC-RESIST cell state transition. Mean of the IPA z-scores in all conditions investigated for MGG4 cells (VC-Resist dataset, NesHI MGG4, in vivo IR and TMZ-treated MGG4 and BV-cocult MGG4 cells) are shown. Positive z-scores mean a predicted activation of the specific upstream regulator. **D,** Representative immunoblots showing YAP and S127-phosphorylation of YAP in NesHI MGG4 cells, in IR and in CM-treated MGG4 cells. Data are representative results of three independent experiments and quantified in Fig. 8B. **E,** Quantitative RT-PCR analysis of Yap1 expression in siCTRL and siYAP1 MGG4 cells. Data are means±SEM (n=3, ***p<0.001; paired two-sided t-test, technical replicates). **F,** Cell death percentage (Sytox positive cells) in control (CT) and b.End3 condition media (CM) MGG4 cells transfected with scramble siRNA (siCTRL) or against YAP1 (siYAP1). Data are means±SEM (n=3) **G,** Proportion of NesHI cells in MGG4 unsorted cells in control (CT) vs condition media (CM) or naïve vs irradiated treated with escalating doses of the FGF-trap NSC12. Data are means±SEM (n=3, *p<0.05 vs no NSC12; two-way ANOVA, Tukey's multiple comparisons test) **H,** NesHI vs NesLO cell death (Sytox+ cells) in Nestin$^{P}$-dTomato MGG4 cells treated or not with NSC12. Data are means±SEM.

# Supplementary Figure 16

A

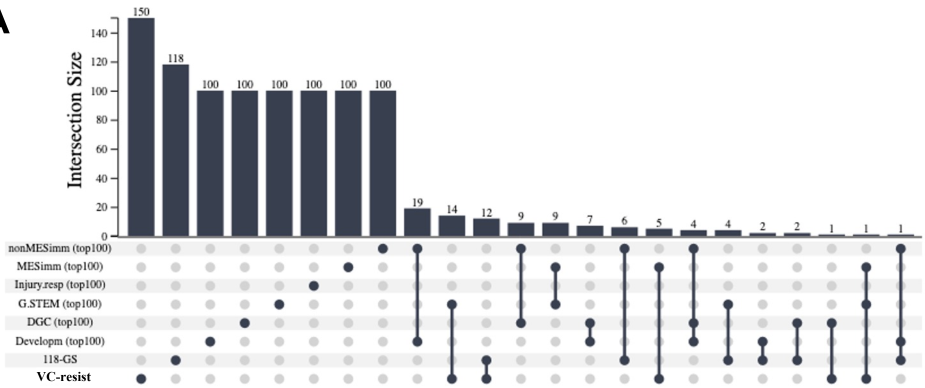


B

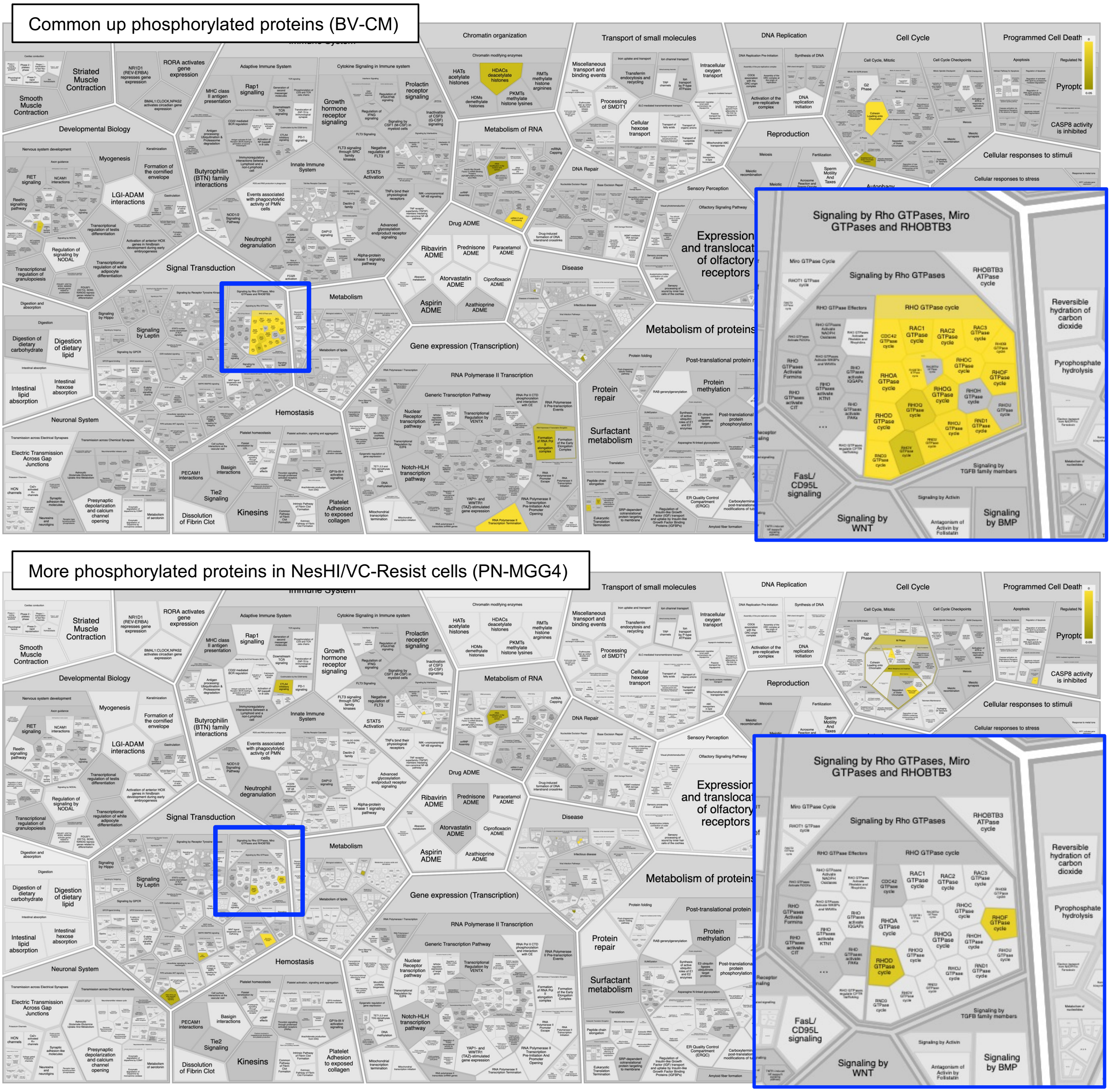

Supplementary Figure 16 – **Reactome of the common phosphorylated proteins**

**A,** UpSet plot showing the lack of significant overlap of the VC-Resist geneset with the previously published scRNA-Seq signatures. **B,** Reactome over-representation analysis of the common proteins more phosphorylated upon blood vessel treatment in both MGG4 and GL261 (top) and the more phosphorylated in NesHI MGG4 cells (botton). Inset: magnification of the Rho pathway signaling, commonly enriched in both datasets. The whole RNA-Seq result was used to perform the PADOG analysis (i.e., a weighted geneset analysis method that down-weighs genes that are present in many pathways).

# Supplementary Figure 17

## A

### NesHI cells gating strategy

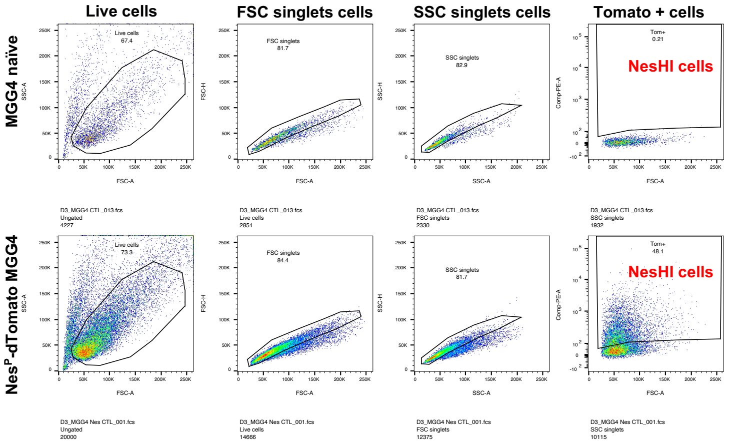


## B

### Cell death cells gating strategy

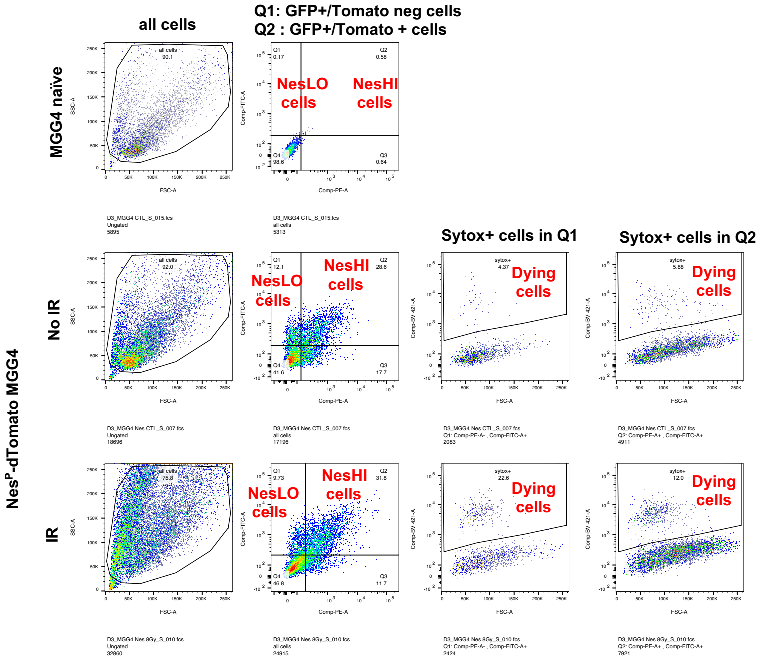


## C

### YAP activation gating strategy

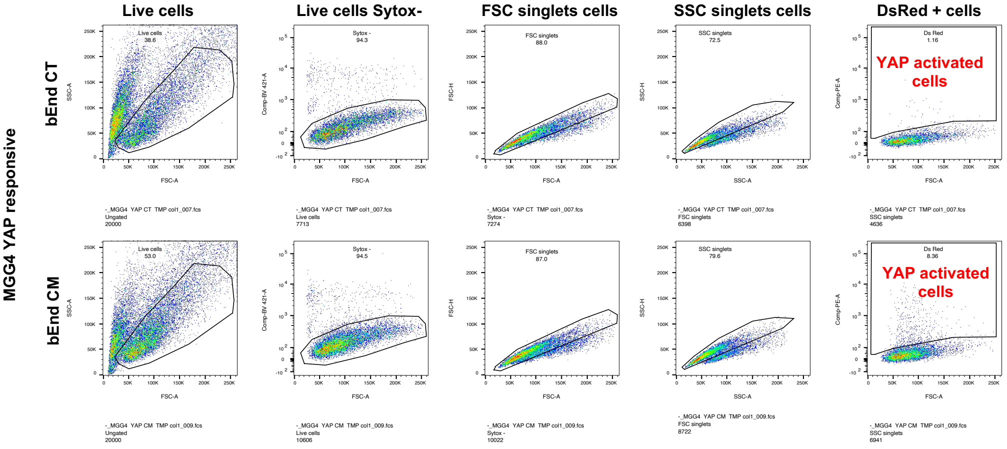

Supplementary Figure 17 – **FACS gating strategy**

**A,** Nestin High gating strategy. Viable cells were first gated based on their size and granularity on FSC-A/SSC-A parameters. Doublets were excluded using both FSC-A/FSC-H and SSC-A/SSC-H parameters. Finally, cells were plotted for dTomato against FSC-A parameters to identify NesHI cells. This gating strategy was used for figures: 1J-O; 2D,G-I; 4M; 6B,F; 7K-L; S2C-E; S3C,F; S15G. **B,** Cell death gating strategy. Viable cells were first gated based on their size and granularity on FSC-A/SSC-A parameters. Doublets were excluded using both FSC-A/FSC-H and SSC-A/SSC-H parameters. Cells were plotted for their FITC and dTomato parameters to identify NesLO and NesHI cells. Sytox+ cells were then identified using BV-421 parameter. This gating strategy was used for figures: 4I-J; 6J; 7G; S3C-F; S9A-C; S13G; S15F-H. **C,** YAP activation gating strategy. Viable cells were first gated based on their size and granularity on FSC-A/SSC-A parameters. Dead cells were excluded by gating on Sytox negative cells using BV421 parameter. Doublets were excluded using both FSC-A/FSC-H and SSC-A/SSC-H parameters. Cells were plotted for dsRed (PE channel) against FSC-A parameters to identify YAP activated cells.

# Supplementary Figure 18

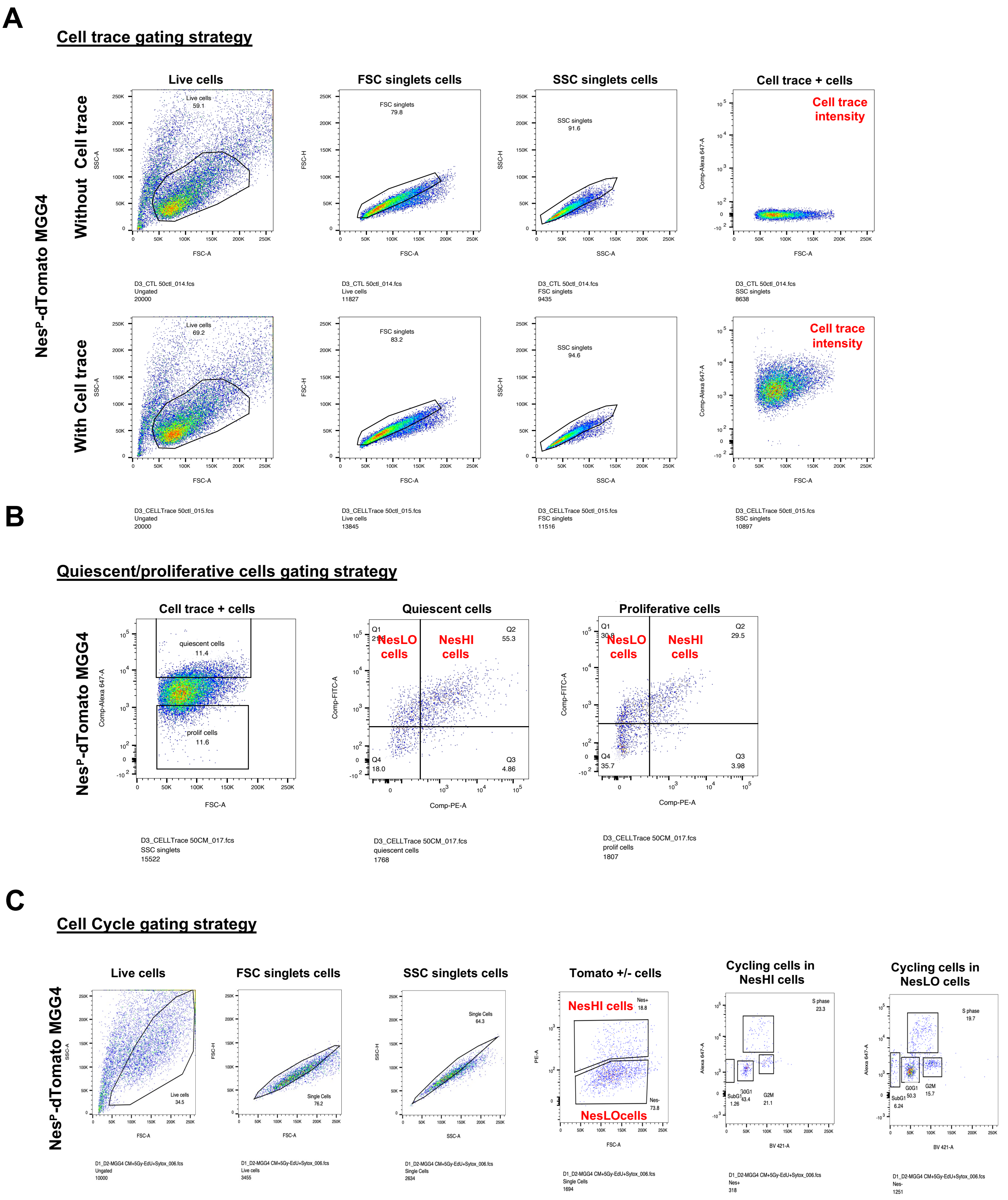

Supplementary Figure 18 – **FACS gating strategy**

**A, Cell trace gating strategy**. Viable cells were first gated based on their size and granularity on FSC-A/SSC-A parameters. Doublets were excluded using both FSC-A/FSC-H and SSC-A/SSC-H parameters. Cells were plotted for Alexa 647 (cell trace) against FSC-A parameters to determine Cell trace intensity in the all population. This gating strategy was used for figures: 6I; S10A-B; S13F. **B, Quiescent/proliferative cells gating strategy**. Cells were plotted for Alexa 647 (cell trace) against FSC-A parameters to determine quiescent and proliferative cells subpopulations. NesLO and NesHI cells were then gated using the FITC and dTomato parameters in each subpopulation. This gating strategy was used for figure 4K. **C, Cell cycle gating strategy.** Viable cells were first gated based on their size and granularity on FSC-A/SSC-A parameters. Doublets were excluded using both FSC-A/FSC-H and SSC-A/SSC-H parameters. Cells were plotted for their FITC and dTomato parameters to identify NesLO and NesHI cells. Cycling cells were then identified using Alexa 647-A (EDU) and BV421-A (Sytox) parameters in each subpopulation. This gating strategy was used for figures: 4L; S10C; S13E-H.
