# Supplementary material for: VC-resist glioblastoma cell state: vessel co-option as a key driver of chemoradiation resistance
Source: Nat Commun. 2024 Apr 29;15:3602. doi: 10.1038/s41467-024-47985-z (PMC11058782; doi:10.1038/s41467-024-47985-z)
Supplement: Supplementary file 2 — Reporting Summary [file 41467_2024_47985_MOESM2_ESM.pdf]

Corresponding author(s): Giorgio Seano, PhD

Last updated by author(s): 2024/03/30

Reporting Summary

Nature Portfolio wishes to improve the reproducibility of the work that we publish. This form provides structure for consistency and transparency in reporting. For further information on Nature Portfolio policies, see our Editorial Policies and the Editorial Policy Checklist.

Statistics

For all statistical analyses, confirm that the following items are present in the figure legend, table legend, main text, or Methods section.

n/a	Confirmed	
☐	☒	The exact sample size (*n*) for each experimental group/condition, given as a discrete number and unit of measurement
☐	☒	A statement on whether measurements were taken from distinct samples or whether the same sample was measured repeatedly
☐	☒	The statistical test(s) used AND whether they are one- or two-sided<br>*Only common tests should be described solely by name; describe more complex techniques in the Methods section.*
☒	☐	A description of all covariates tested
☐	☒	A description of any assumptions or corrections, such as tests of normality and adjustment for multiple comparisons
☐	☒	A full description of the statistical parameters including central tendency (e.g. means) or other basic estimates (e.g. regression coefficient) AND variation (e.g. standard deviation) or associated estimates of uncertainty (e.g. confidence intervals)
☐	☒	For null hypothesis testing, the test statistic (e.g. *F*, *t*, *r*) with confidence intervals, effect sizes, degrees of freedom and *P* value noted<br>*Give P values as exact values whenever suitable.*
☒	☐	For Bayesian analysis, information on the choice of priors and Markov chain Monte Carlo settings
☒	☐	For hierarchical and complex designs, identification of the appropriate level for tests and full reporting of outcomes
☐	☒	Estimates of effect sizes (e.g. Cohen's *d*, Pearson's *r*), indicating how they were calculated

Our web collection on statistics for biologists contains articles on many of the points above.

Software and code

Policy information about availability of computer code

Data collection

Data was collected on R-4.3.2, Microsoft Excel and Prism 8 (GraphPad Software Inc.).

Data analysis

Bulk RNA-Seq: The reads obtained were processed using the Curie Institute Nextflow RNA-seq analysis pipeline v3.1.8 (in-vivo MGG4, MGG18, GL261, MGG4 TMZ), v3.1.7 (BV co-culture), v3.1.5 (MGG4 Tom high). Briefly, reads quality was assessed using FastQC and mapped to the reference genome (hg19/GRCh37 or mm10/GRCm38 for GL261) using STAR software. Finally, raw reads count tables were generated using STAR. Software versions and full pipelines are available at https://github.com/bioinfo-pf-curie/RNA-seq/

scRNA-Seq: Sequencing output reads were converted to FASTQ files using bcl2fastq (v2.20) and aligned to the hg38 reference genome with Kallisto aligner (v0.46.2) 77. The resulting bus files were corrected, sorted then raw count matrix was generated with bustools (v.0.40.0) programs 78. The raw matrix was filtered with the function emptyDrops from the DropletUtils package, using the method 'cellranger' 79. Filtered raw counts data was imported into Seurat R package (v4.0.3) 80 for further processing and analysis. Raw transcript counts of gene-cell matrices were filtered to remove cells with total UMI counts lower than 4000 and higher than 11000; and cells with more than 20% mitochondrial genes. The UMI counts matrices were then normalized with Satija's lab SCTransform method. Cell cycle scores were calculated with Seurat and used to regress out the cell cycle signal during normalization. Finally, the different datasets were integrated with Seurat's anchors method using 3000 features.
Dimension reduction. Linear dimension reduction (principal component analysis) was applied on the 3000 genes with the highest variance identified by SCTransform and the number of principal components used in downstream analyses, 30 was chosen considering Seurat's PCHeatmap and Elbowplot. Seurat's implementation of Uniform manifold approximation and projection (UMAP) was applied on the reduced data for visualization in two-dimensional space.
Cluster analysis. Clusters were identified with IKAP algorithm 25. IKAP uses Seurat graph-based unsupervised clustering. It generates various candidate clusterings by tuning Seurat's algorithm parameters, then computes a gap statistics for each clustering. The clustering with the

highest gap increase is then selected. CerebroApp's getMarkerGenes, which internally calls Seurat's FindAllMarkers, was used to identify cluster-specific markers. In order to select widely and significantly overexpressed genes, the minimal logFC was set to 0,5 and the minimum percentage of cells to 0.75. For each cluster, FindMarkers function was used to calculate DE genes between treated and untreated cells.
Molecular 4-states cell classification. Cells were classified according to Suva's lab method and the gene signatures they generated 10. For each set of genes (Gj), a score was attributed to each cell. This score was calculated as the difference between the average relative expression of the genes in (Gj) and the average relative expression in a control gene set, i.e. Score(G,) = av(Er(G(j,i)) – av(Er(Gj control, i). The control gene set was defined as first binning all analyzed genes into 30 bins of aggregate expression levels and then, for each gene in the gene-set (Gj), randomly selecting 100 genes from the same expression bin. The cell was then attributed the state with the highest score between APC-like, NPC-like, AC-like, MES-like. For 2D representation, the y coordinate was calculated by the formula y = max(SCopc,SCnpc) – max(SCac,Scmes). The sign of the y coordinate allowed to separate cells into OPC/NPC (y>0) versus AC/MES (y<0). The x coordinate was defined for OPC/NC cells as x = log2(|SCopc – SCnpc|+1) and for AC/MES cells as x = log2(|SCac – SCmes|+1).
RNA velocity. RNA velocity was analyzed using the aligner velocyto (v0.17.17) and the scvelo toolkit (v0,2,3) 81,82. Count matrices of pre-mature (unspliced) and mature (spliced) RNAs were obtained with velocyto. Scvelo functions were used with defaults parameters to filter and normalize the data. Future cell state was computed using a likelihood-based dynamical model (funtion velocity, diff_kinetics=True). For results representation, Seurat's Umap representations were imported and scvelo functions were used to project velocities into Umap's low dimension space.
Data Visualization. For most steps of the analysis, plots were generated either with Seurat's visualization functions or with R package ggplot2 or CerebroApp visualization and export functions. For RNA velocity, plots were generated with scvelo's visualization functions.

Proteomics: Data analysis. For identification, the data were searched against the Homo Sapiens (UP000005640_9606) UniProt database for MGG4 and PN-MGG4 samples and against the Mus Musculus (UP000000589 database downloaded 03/2020) for GL261 samples using Sequest HT through Proteome Discoverer (PD version 2.4). Enzyme specificity was set to trypsin and a maximum of two missed cleavage sites were allowed. Oxidized methionine, N-terminal acetylation, methionine loss and methionine acetylation loss were set as variable modifications. Phospho serine, threonine and tyrosines were also set as variable modifications in phosphoproteome analyses. Maximum allowed mass deviation was set to 10ppm for monoisotopic precursor ions and 0.02 Da for MS/MS peaks from the Orbitrap Exploris 480 instrument and 0.6 Da for MS/MS peaks from the Orbitrap Eclipse Tribrid instrument. The resulting files were further processed using myProMS95 https://github.com/bioinfo-pf-curie/myproms v.3.9.3. False-discovery rate (FDR) was calculated using Percolator 96 and was set to 1% at the peptide level for the whole study. Label-free quantification was performed using peptide extracted ion chromatograms (XICs), computed with MassChroQ 97 v.2.2.21. For protein quantification, XICs from proteotypic peptides shared between compared conditions (TopN matching for proteome setting and simple ratios for phosphoproteome) with missed cleavages were used. Median and scale normalization at peptide level was applied on the total signal to correct the XICs for each biological replicate (N=5). The phosphosite localization accuracy was estimated by using the PtmRS node in PD, in PhosphoRS mode only. Phosphosites with a localization site probability greater than 75% were quantified at the peptide level. To estimate the significance of the change in protein abundance, a linear model (adjusted on peptides and biological replicates) was performed, and p-values were adjusted using the Benjamini–Hochberg FDR procedure. Proteins with at least 3 total peptides in all replicates (n=5) and an adjusted p-value ≤ 0.05 were considered significantly enriched in sample comparisons. Unique proteins were considered with at least three total peptides in all replicates. Kinase Enrichment Analysis (KEA) was performed using the web-based platform KEA3 (https://maayanlab.cloud/kea3/)98 with a selected list of proteins (more than 0.8 of log2 fold change and shared between MGG4 and GL261 datasets) or KSEAapp (https://github.com/casecpb/KSEAapp/)99. For KSEAapp, KEA was performed with a p-value threshold at 0.01 and a minimum of 5 substrates per kinase.

For manuscripts utilizing custom algorithms or software that are central to the research but not yet described in published literature, software must be made available to editors and reviewers. We strongly encourage code deposition in a community repository (e.g. GitHub). See the Nature Portfolio guidelines for submitting code & software for further information.

Data

Policy information about availability of data

All manuscripts must include a data availability statement. This statement should provide the following information, where applicable:

- Accession codes, unique identifiers, or web links for publicly available datasets
- A description of any restrictions on data availability
- For clinical datasets or third party data, please ensure that the statement adheres to our policy

The data that support the findings of this study are available from the corresponding author (giorgio.seano@curie.fr) upon reasonable request. RNA-seq data have been deposited in NCBI's GEO and are accessible through GEO Series accession number GSE218860. The mass spectrometry proteomics data have been deposited to the ProteomeXchange Consortium via the PRIDE partner repository with the dataset identifier PXD042606.

Research involving human participants, their data, or biological material

Policy information about studies with human participants or human data. See also policy information about sex, gender (identity/presentation), and sexual orientation and race, ethnicity and racism.

Reporting on sex and gender	N/A
Reporting on race, ethnicity, or other socially relevant groupings	N/A
Population characteristics	N/A
Recruitment	N/A
Ethics oversight	*Identify the organization(s) that approved the study protocol.*

Note that full information on the approval of the study protocol must also be provided in the manuscript.

Field-specific reporting

Please select the one below that is the best fit for your research. If you are not sure, read the appropriate sections before making your selection.

☒ Life sciences ☐ Behavioural & social sciences ☐ Ecological, evolutionary & environmental sciences

For a reference copy of the document with all sections, see nature.com/documents/nr-reporting-summary-flat.pdf

Life sciences study design

All studies must disclose on these points even when the disclosure is negative.

Sample size	No a priori sample size calculation.
Data exclusions	No data excluded.
Replication	All results shown in the manuscript are the outcomes of at least three biological replicates (different batches of cells) and independent experiments.
Randomization	N/A
Blinding	The investigators were blinded to group allocation during data collection. Blinding during image analysis was used for all the in vivo experiments.

Reporting for specific materials, systems and methods

We require information from authors about some types of materials, experimental systems and methods used in many studies. Here, indicate whether each material, system or method listed is relevant to your study. If you are not sure if a list item applies to your research, read the appropriate section before selecting a response.

Materials & experimental systems

n/a	Involved in the study	
☐	☒	Antibodies
☐	☒	Eukaryotic cell lines
☒	☐	Palaeontology and archaeology
☐	☒	Animals and other organisms
☒	☐	Clinical data
☒	☐	Dual use research of concern
☒	☐	Plants

Methods

n/a	Involved in the study	
☒	☐	ChIP-seq
☐	☒	Flow cytometry
☒	☐	MRI-based neuroimaging

Antibodies

Antibodies used	Tissue IHC: anti-Nestin (10C2; 1:200; Ebiosciences or #PAS-82905, 1:1000, ThermoScientific), anti-hMito (113-1; 1:200 or 1:50; Millipore), anti-CD31 PECAM (#AF-3628, 1:100, R&D Systems) and anti-CD34 (EPS73Y; 1:500; Abcam).<br>IF on in vitro cells: YAP (D8H1X, XP® Rabbit mAb #14074S)
Validation	Tissue IHC:<br>- anti-Nestin (10C2; 1:200; Ebiosciences): Positive control = WB: U251 cells. ICC: U251 cells. Human brain tissue.<br>- anti-hMito (113-1; 1:200; Millipore): Positive control = WB: HeLa cell lysate. ICC/IF: HeLa and U87 cells. IHC-P: Human breast cancer tissue. Flow Cyt: HepG2 cells.<br>- anti-CD31 PECAM (#AF-3628, 1:100, R&D Systems): Positive control = lysates of bEnd.3 mouse endothelioma cell line.<br>- anti-CD34 (EPS73Y; 1:500; Abcam): Positive control = Flow Cyt (intra): TF-1 cells. WB: TF-1 cell lysate. IHC-P: Human kidney tissue; Mouse normal brain, prostate and kidney tissues; Rat kidney tissue. ICC/IF: HUVEC cells; Human embryonic stem cell-derived endothelial cells. IHC-Fr: Mouse and rat lung tissue. Rat kidney. IP: TF-1 cell lysate.<br><br>IF on in vitro cells:<br>YAP (D8H1X, XP® Rabbit mAb #14074S): Positive control = Flow Cyt (intra): TF-1 cells. WB: TF-1 cell lysate. IHC-P: Human kidney tissue; Mouse normal brain, prostate and kidney tissues; Rat kidney tissue. ICC/IF: HUVEC cells; Human embryonic stem cell-derived endothelial cells. IHC-Fr: Mouse and rat lung tissue. Rat kidney. IP: TF-1 cell lysate.

Eukaryotic cell lines

Policy information about cell lines and Sex and Gender in Research

Cell line source(s)	The human glioblastoma patient-derived cell lines MGG4 and MGG18 were obtained from Dr. Wakimoto (Dept. of Neurosurgery, Massachusetts General Hospital, USA) 24, the BG5, the BG7 and the NCH421k from Dr. Daubon (IBGC, Bordeaux, France), the BT18, BT27 and ZH305 from Dr. Le Joncour (Faculty of Medicine, University of Helsinki, Finland), the T98 from Dr. Dutreix (Institut Curie, Paris, France) and GSC2 were previously isolated by our team 68. The mouse glioblastoma GL261 cell line was purchased from The Jackson Laboratory (Bar Harbor, USA) and the mGB1 and mGB2 were provided by Dr. Angel (DKFZ, Heidelberg, Germany).
Authentication	Cells were authenticated and cultured for no more than 15 passages.
Mycoplasma contamination	All cell lines were repeatedly tested and were negative for mycoplasma using the Mycoplasma Detection Kit (MB Minerva Biolabs, #117048).
Commonly misidentified lines (See ICLAC register)	No commonly misidentified cell lines were used in the study

Animals and other research organisms

Policy information about studies involving animals; ARRIVE guidelines recommended for reporting animal research, and Sex and Gender in Research

Laboratory animals	7 to 8-week-old female nude mice (Charles River) were used in our studies. These mice were housed in temperature and light controlled facility with maximum five mice per cage. Mice were routinely observed and weighted to ensure that interventions were well tolerated.
Wild animals	No wild animals were used in the study.
Reporting on sex	7 to 8-week-old female nude mice (Charles River) were used in our studies.
Field-collected samples	No field collected samples were used in the study.
Ethics oversight	All animal procedures were conducted in compliance with recommendations of the European Community (2010/63/UE). Animal experimental procedures were specifically approved by the ethics committee of Institut Curie (CEEA-IC #118; 2018-010).

Note that full information on the approval of the study protocol must also be provided in the manuscript.

Plants

Seed stocks	No seed stocks were used in the study.
Novel plant genotypes	No novel plant genotypes were used in the study.
Authentication	N/A

Flow Cytometry

Plots

Confirm that:

- ☐ The axis labels state the marker and fluorochrome used (e.g. CD4-FITC).
- ☒ The axis scales are clearly visible. Include numbers along axes only for bottom left plot of group (a 'group' is an analysis of identical markers).
- ☐ All plots are contour plots with outliers or pseudocolor plots.
- ☒ A numerical value for number of cells or percentage (with statistics) is provided.

Methodology

Sample preparation	Sorting: Neurospheres were collected, centrifuged and resuspended as single cell suspension with accutase. Cells were then washed with PBS, centrifuged and resuspended in neurocult media before filtering in a FACS tube. Cells then loaded into the

BD FACSAriaTMIII sorter (BD Biosciences).

FACS analysis: NestinP-dTomato MGG4, MGG18 or GL261 cells were seeded at the optimal density, treated (IR or TMZ) or not and cultured for several days according to the experiment. On the day of FACS analysis, neurospheres of each condition were collected, centrifuged and resuspended with accutase as single cell suspension. Cells were then washed with PBS, centrifuged and resuspended in MACS buffer and divided in 2 FACS tubes: one for the dTomato analysis, the other one for cell death analysis. For reprogramming analysis, cells were loaded into the BD LSRFortessaTM Cell Analyzer (BD Bioscience).

Instrument	Cells then loaded into the BD FACSAriaTMIII sorter or the BD LSRFortessaTM Cell Analyzer (BD Biosciences).
Software	Analysis of all the recorded FACS data were then performed using FlowJo v10.7.2 software (BD Biosciences).
Cell population abundance	N/A
Gating strategy	Sorting: Viable cells were first gated based on their size and granularity on FSC-A/SSC-A parameters. Doublets were excluded using both FSC-A/FSC-H and SSC-A/SSC-H parameters. Finally, cells were plotted for their FITC and dTomato parameters.<br><br>FACS analysis: Live cells were first gated and doublets were then excluded as explained above. NestinP-dTomato cells were detected using the PE channel. MGG4, MGG18 or GL261 naïve cells were used as control. For cell death analysis, Sytox (0.5 µM; Thermo Fisher, #S34857) was added in the flow cytometry tube containing cells and incubated for few minutes. Cells were then loaded into the FACS analyzer. Debris were discarded based on FSC-A/SSC-A parameters and Sytox positive cells were then detected in the BV421 channel. Cells without Sytox of each and all conditions were used as control for proper identification of Sytox positive cells.

☒ Tick this box to confirm that a figure exemplifying the gating strategy is provided in the Supplementary Information.
