# Supplementary material for: VC-resist glioblastoma cell state: vessel co-option as a key driver of chemoradiation resistance
Source: Nat Commun. 2024 Apr 29;15:3602. doi: 10.1038/s41467-024-47985-z (PMC11058782; doi:10.1038/s41467-024-47985-z)
Supplement: Supplementary file 12 — Description of Additional Supplementary Files [file 41467_2024_47985_MOESM12_ESM.pdf]

Supplementary Movie 1 –
Reprogramming and vessel co-option in the organotypic blood vessel co-culture model. Representative time-lapse imaging of the cell state transition of a NesLO cell to NesHI when close to blood vessel.

Supplementary Movie 2 –
Reprogramming and vessel co-option in brain slice. Representative time-lapse confocal video showing the reprogramming of NesLO to NesHI cells when close to blood vessels (lectin, grey) and tumor cells (MGG4-Nes-GFP cell) over time in brain slice organotypic model. Scale bar, 20µm.

Supplementary Data file 1 –
Neurospheres cell lines characterization
- Sheet 1: Dominant states

Supplementary Data file 2 –
CL3 and NesHI gene signatures
- Sheet 1: CL3 DEGs
- Sheet 2: NesHI MGG4 DEGs
- Sheet 3: NesHI MGG18 DEGs
- Sheet 4: NesHI GL261 DEGs
- Sheet 5: Sum-up of the signatures
- Sheet 6: NesHI MGG4 kinase activity analysis

Supplementary Data file 3 –
Multivariate analysis for NesHI/CL3 geneset in TCGA GBM
- Sheet 1: Progression-free survival
- Sheet 2: Overall survival

Supplementary Data file 4 –
BV- and EC-cocult gene and protein signatures
- Sheet 1: BV-cocult MGG4 DEGs
- Sheet 2: EC-cocult GSC2 DEGs
- Sheet 3: BV-CM treated GB cells' common DEPs
- Sheet 4: BV-CM GB cells' kinase activity analysis

Supplementary Data file 5 –
Proteome and phosphoproteome upregulated by BV-CM
- Sheet 1: Proteome
- Sheet 2: Phosphoproteome

Supplementary Data file 6 –
Ingenuity Pathway Analysis
- Sheet 1: IPA upstream transcription factors
